# Response adaptive intervention allocation in stepped‐wedge cluster randomized trials

**DOI:** 10.1002/sim.9317

**Published:** 2022-01-21

**Authors:** Michael J. Grayling, James M. S. Wason, Sofía S. Villar

**Affiliations:** ^1^ Population Health Sciences Institute Newcastle University Newcastle upon Tyne UK; ^2^ MRC Biostatistics Unit, School of Clinical Medicine University of Cambridge Cambridge UK

**Keywords:** adaptive design, clinical trial, interim analysis, multi‐stage, sequential allocation

## Abstract

**Background:**

Stepped‐wedge cluster randomized trial (SW‐CRT) designs are often used when there is a desire to provide an intervention to all enrolled clusters, because of a belief that it will be effective. However, given there should be equipoise at trial commencement, there has been discussion around whether a pre‐trial decision to provide the intervention to all clusters is appropriate. In pharmaceutical drug development, a solution to a similar desire to provide more patients with an effective treatment is to use a response adaptive (RA) design.

**Methods:**

We introduce a way in which RA design could be incorporated in an SW‐CRT, permitting modification of the intervention allocation during the trial. The proposed framework explicitly permits a balance to be sought between power and patient benefit considerations. A simulation study evaluates the methodology.

**Results:**

In one scenario, for one particular RA design, the proportion of cluster‐periods spent in the intervention condition was observed to increase from 32.2% to 67.9% as the intervention effect was increased. A cost of this was a 6.2% power drop compared to a design that maximized power by fixing the proportion of time in the intervention condition at 45.0%, regardless of the intervention effect.

**Conclusions:**

An RA approach may be most applicable to settings for which the intervention has substantial individual or societal benefit considerations, potentially in combination with notable safety concerns. In such a setting, the proposed methodology may routinely provide the desired adaptability of the roll‐out speed, with only a small cost to the study's power.

## 1 INTRODUCTION

Stepped‐wedge cluster randomized trials (SW‐CRTs) roll an intervention out over several time periods, with all clusters typically ending the trial in the intervention condition.^1^ SW‐CRTs have been favored for several reasons, including that sequential roll‐out may assist with logistical constraints. However, SW‐CRTs have not been without criticism. In particular, there has been much discussion of another reason commonly given for using an SW‐CRT: a strong belief that the intervention will do more good than harm, which implies its allocation to all clusters is advantageous. Kotz et al^2^ argued this makes SW‐CRTs troubling, because a decision to provide the intervention to all clusters should not be made when its effectiveness remains unproven.

It has been pointed out, however, that the design has typically been used when the intervention has been “shown to be effective in more controlled…settings.”^3^ This raises a further important issue though around whether there can be equipoise in an SW‐CRT if there is a strong belief, perhaps emboldened by previous studies, that the intervention will be effective. Given that it has been argued “genuine uncertainty…about the preferred treatment” is a prerequisite for conducting a randomized trial,^4^ this calls in to question when an SW‐CRT could be conducted.

Prost et al^5^ suggested a constructive solution to this question is to consider whether the evidence in favor of the intervention is sufficient to suggest equipoise is truly disturbed. While there may be a consensus that the intervention will be beneficial, there may still be true uncertainty about its effectiveness in a given context. Thus equipoise may still apply. Ultimately, it has been argued SW‐CRTs in which equipoise is disturbed should not be undertaken.^6^ Given equipoise, though, we return to the scenario above where there may then be concern around a decision to provide the intervention to all clusters. This could be particularly true of closed‐cohort SW‐CRT designs, where all participants would then receive the intervention, or when the intervention is associated with substantial safety considerations.

In drug development, response adaptive (RA) design has been suggested as way to address deviations from equipoise that could arise from data collected during a trial. To introduce RA design, consider a parallel two‐arm individually randomized trial. With RA design, the trial incorporates interim analyses at which the allocation ratio can be modified, with the standard being to increase allocation to the best performing treatment. The number of patients expected to receive the best treatment is then increased. If the endpoint used to evaluate the treatments is related to patient benefit, then on average this provides an advantage to patients enrolled on the trial compared to fixed 1:1 randomization. Importantly, any decision to increase allocation to a particular treatment is made using concurrent study data; unlike in an SW‐CRT, which makes this decision pre‐trial. For an overview of RA trial design, see one of several recent monographs^7^, ^8^, ^9^ or the recent review by Robertson et al.^10^

It is interesting therefore to ask whether and/or how a conventional SW‐CRT could be modified to incorporate RA intervention allocation, enabling an intervention to be provided to more participants when it is effective, but its roll‐out slowed, or stopped, when ineffective. In this article, we describe a flexible framework for modifying an SW‐CRT allocation matrix at a series of interim analyses. To evaluate the framework, we present the results of an extensive simulation study. To conclude, we describe several practical issues associated with utilizing an RA SW‐CRT design and discuss when it may be useful.

## 2 METHODS

### 2.1 Design setting

We suppose an SW‐CRT will be used to compare an intervention to a control; aiming to contrast an RA SW‐CRT with its conventional fixed‐sample analog. We suppose this fixed‐sample SW‐CRT has been designed, omitting discussion on how this can be achieved as it is has been covered elsewhere.^11^, ^12^, ^13^, ^14^ Thus, we assume the number of clusters C>1, time periods P>1, and measurements m>1 per cluster‐period, have been specified. We consider designs where the *m* measurements from each cluster‐period are from the same (closed‐cohort design) or from different (cross‐sectional design) participants. We comment on application to open‐cohort designs in Section 4. We also suppose a treatment allocation matrix has been nominated, $X=\{X_{ij}\}$, i=1,…,C, j=1,…,P, with $X_{ij}=1$ implying cluster *i* receives the intervention in time period *j*, and $X_{ij}=0$ otherwise. We refer to this as the initially planned allocation matrix.

We denote the responses to be accrued up to time period *p*, 1≤p≤P, by $Y_{p}$. Specifically, suppose measurement k=1,…,m from cluster i=1,…,C in period j=1,…,P is denoted by $Y_{ijk}$. Then

$$\begin{array}{ll} Y_{p} & =(Y_{111},\ldots ,Y_{11m},Y_{121},\ldots ,Y_{12m},\ldots ,Y_{1p1},\ldots ,Y_{1pm},\ldots ,\;\\ & \;Y_{C11},\ldots ,Y_{C1m},Y_{C21},\ldots ,Y_{C2m},\ldots ,Y_{Cp1},\ldots ,Y_{Cpm}{)}^{⊤}.\; \end{array}$$

We suppose that at the design stage a particular linear mixed model has been designated for data analysis, and thus it has been assumed $Y_{p}∼N(D_{p|X}\theta ,\sum_{p|X})$, for known nonsingular covariance matrix $\sum_{p|X}$, design matrix $D_{p|X}$, and fixed effects $\theta ={\left(\theta_{1},\ldots ,\theta_{q}\right)}^{⊤}$. As we see in our simulation study later, θ would typically be expected to include an intercept term, factors to adjust for time effects, and an effect for the intervention relative to the control. Furthermore, we note that a large number of possible analysis models have been proposed for SW‐CRTs; see Li et al^15^ for an overview of many of these. To emphasize, our designations above allow for any of these that work within a linear mixed model framework, including those assuming a decaying correlation structure. Finally, we note that we explicitly state the dependence of $\sum_{p|X}$ and $D_{p|X}$ upon *X* since *X* will later be treated as a variable.

We assume the goal is to make inference on the intervention's effect relative to the control. We suppose this is estimated through $\theta_{q}$ and refer to this from here as θ for brevity. We assume that the one‐sided hypothesis $H_{0}:\theta \leq 0$ will be tested, with a type‐I error‐rate of α∈(0,1) desired when θ=0. Later, we also compare designs in terms of their power when θ=δ, for specified δ>0, with the target to achieve power of 1−β∈(0,1).

Note the generalized least squares estimate of θ after time period *p* is ${\hat{\theta }}_{p|X}={\left(D_{p|X}^{⊤}\sum_{p|X}^{-1}D_{p|X}\right)}^{-1}D_{p|X}^{⊤}\sum_{p|X}^{-1}Y_{p}$. Extracting the last element, ${\hat{\theta }}_{p|X}$, the following Wald test statistic can be calculated

$$Z_{p|X}=\frac{{\hat{\theta }}_{p|X}}{{\left\{\mathrm{Var}({\hat{\theta }}_{p|X})\right\}}^{1/2}}=\frac{{\hat{\theta }}_{p|X}}{{\left[{\left\{{\left(D_{p|X}^{⊤}\sum_{p|X}^{-1}D_{p|X}\right)}^{-1}\right\}}_{qq}\right]}^{1/2}}:={\hat{\theta }}_{p|X}I_{p|X}^{1/2}.$$

A conventional SW‐CRT would proceed by enrolling *C* clusters, accruing *m* measurements per cluster in time periods 1,…,P, and allocating treatments according to *X*. Its final analysis could be conducted by assessing whether $Z_{P|X}>\Phi^{-1}(1-\alpha )$. Our aim, as discussed, is to describe methodology through which *X* may be altered mid‐trial. Note that it is only *X* we modify; to provide a fairer comparison to the corresponding conventional fixed‐sample SW‐CRT we assume the initial values of *m*, *C*, and *P* are not altered at the interim analyses.

### 2.2 Response adaptive stepped‐wedge cluster randomized trials

First, a set of integers $\left\{p_{1},\ldots ,p_{L}\right\}$, with $1\leq p_{l_{1}}<p_{l_{2}}\leq P-1$ for $1\leq l_{1}<l_{2}\leq L$, are specified. Then, *L* interim analyses at which the allocation matrix may be altered are conducted; after time periods $p\in \{p_{1},\ldots ,p_{L}\}$. Accordingly, we denote by $X_{p}=\{X_{pij}\}$, 1≤p≤P, the matrix containing the allocations used in time periods 1,…,p and those planned for time periods p+1,…,P. We set $X_{1}=\cdots =X_{p_{1}}=X$, using the initially planned allocation matrix *X*.

Next, sets $X_{X_{p}}$ are specified, giving the possible allocation matrices to be chosen from at the analysis following time period *p*, dependent on the value of $X_{p}$. That is, $X_{p+1}{\in X}_{X_{p}}$. Arbitrary restrictions can be placed on the $X_{X_{p}}$ as are desired. In all instances though, $X_{X_{p}}$ must consist of C×P binary matrices whose elements in columns 1,…,p match those from $X_{p}$ (as past allocations cannot be changed), and whose elements are such that if $X_{pip}=1$ then $X_{pip+1}=\cdots =X_{piP}=1$ (as clusters cannot switch back to the control). Thus, formally, we must always have that

$$\begin{array}{ll} & X_{X_{p}}{\subseteq M}_{X_{p}}=\left[M=\{M_{ij}\}\in {𝕄}_{CP}(\{0,1\}):\forall (i,j)\in \{1,\ldots ,C\}\times \{1,\ldots ,p\}\;M_{ij}=X_{pij},\right.\\ & \;\left.\;\forall i\in \{1,\ldots ,C\}\;X_{pip}=1\Rightarrow M_{ip+1}=\cdots =M_{iP}=1\right]. \end{array}$$

Note that $X_{p}{\in M}_{X_{p}}$, so it is always possible to ensure $X_{X_{p}}\neq \emptyset$.

To illustrate the possible specification of $X_{X_{p}}$ more clearly, consider an example with C=P=4 and an interim analysis conducted after time period 2. Suppose that

$$X_{1}=X_{2}=\left(\begin{array}{cccc} 1 & 1 & 1 & 1\\ 0 & 1 & 1 & 1\\ 0 & 0 & 1 & 1\\ 0 & 0 & 0 & 1 \end{array}\right).$$

Placing no restrictions on $X_{X_{2}}$ beyond those which are always required (ie, $X_{X_{2}}{=M}_{X_{2}}$)

$$\begin{array}{ll} X_{X_{2}}=\left\{\left(\begin{array}{cccc} 1 & 1 & 1 & 1\\ 0 & 1 & 1 & 1\\ 0 & 0 & 1 & 1\\ 0 & 0 & 1 & 1 \end{array}\right),\left(\begin{array}{cccc} 1 & 1 & 1 & 1\\ 0 & 1 & 1 & 1\\ 0 & 0 & 1 & 1\\ 0 & 0 & 0 & 1 \end{array}\right),\left(\begin{array}{cccc} 1 & 1 & 1 & 1\\ 0 & 1 & 1 & 1\\ 0 & 0 & 1 & 1\\ 0 & 0 & 0 & 0 \end{array}\right),\left(\begin{array}{cccc} 1 & 1 & 1 & 1\\ 0 & 1 & 1 & 1\\ 0 & 0 & 0 & 1\\ 0 & 0 & 0 & 1 \end{array}\right),\left(\begin{array}{cccc} 1 & 1 & 1 & 1\\ 0 & 1 & 1 & 1\\ 0 & 0 & 0 & 1\\ 0 & 0 & 0 & 0 \end{array}\right),\left(\begin{array}{cccc} 1 & 1 & 1 & 1\\ 0 & 1 & 1 & 1\\ 0 & 0 & 0 & 0\\ 0 & 0 & 0 & 0 \end{array}\right)\right\}. & \end{array}$$

If we wished to ensure that all clusters receive the intervention by the trial's completion, we would modify the above to

$$\begin{array}{ll} X_{X_{2}}=\left\{\left(\begin{array}{cccc} 1 & 1 & 1 & 1\\ 0 & 1 & 1 & 1\\ 0 & 0 & 1 & 1\\ 0 & 0 & 1 & 1 \end{array}\right),\left(\begin{array}{cccc} 1 & 1 & 1 & 1\\ 0 & 1 & 1 & 1\\ 0 & 0 & 1 & 1\\ 0 & 0 & 0 & 1 \end{array}\right),\left(\begin{array}{cccc} 1 & 1 & 1 & 1\\ 0 & 1 & 1 & 1\\ 0 & 0 & 0 & 1\\ 0 & 0 & 0 & 1 \end{array}\right)\right\}. & \end{array}$$

Note that we order the sequences in the allocation matrices such that a nonincreasing proportion of time is spent in the intervention condition. This removes any degeneracy in the choice of possible allocation matrices.

The remaining component required is a function s(·) such that $s(X_{p+1})$ provides a *score* associated with a choice of $X_{p+1}{\in X}_{X_{p}}$. Our approach is then to set

$$X_{p+1}=\underset{X{\in X}_{X_{p}}}{\text{argmax}}s(X).$$

In practice, s(·) could be defined in any way that reasonably evaluates the suitability of $X_{p+1}$. Our approach is to specify s(·) to permit a balance to be sought between desires to (i) maximize allocation to the most effective arm and (ii) maximize power. We set

$$s(X_{p+1})=w\frac{I_{P|X_{p+1}}}{\max_{X^{\prime }{\in X}_{X_{p}}}I_{P|X^{\prime }}}+(1-w)\frac{b(X_{p+1})}{\max_{X^{\prime \prime }{\in X}_{X_{p}}}b(X^{\prime \prime })},\;w\in [0,1].$$

Here, b(·) assesses the performance of $X_{p+1}$ in terms of whether it allocates clusters to the most effective arm (ie, it monitors *patient benefit* considerations). Note the term involving the information levels $I_{P|X_{p+1}}$ evaluates $X_{p+1}$ in terms of the power it likely provides. Thus, *w* is an explicit weight balancing (i) and (ii) above. Note the two factors are rescaled because they exist on different scales. Furthermore, w∈{0,1} should usually be avoided as a means to breaking ties between designs with identical values for $I_{P|X_{p+1}}$ or $b(X_{p+1})$.

The above formulation has been used previously in RA design, for example, for sequence specification in individually randomized crossover trials.^16^ Nonetheless, specifying b(·) is complex for SW‐CRTs because allocation is to be adapted for clusters already in the trial. In practice, there may be good reason to make b(·) a complex function that, for example, incorporates penalties for the speed or cost of the intervention roll‐out if its availability is limited. Here, we consider a function of arguably more general utility, using only current evidence of effectiveness to guide allocation.

It is logical to insist that as $Z_{p|X_{p}}$ increases, b(·) should score designs switching a larger number of clusters to the intervention more highly. It is thus desirable to ensure that when $Z_{p|X_{p}}\to \infty$ the allocation matrix that switches all clusters to the intervention immediately is recommended. Similarly, as $Z_{p|X_{p}}\to -\infty$, the matrix that switches no additional clusters to the intervention should be recommended. Many functions will have these properties. In the Supplementary Material, we describe a form for b(·) that could be useful if the desire is to only alter the design for extreme intervention effects. To more clearly describe the benefits of RA SW‐CRTs, we focus here on a probabilistic form for b(·) that can recommend a broader range of designs, taking

$$\begin{array}{ll} b(X_{p+1}) & =\mathbb{P}\left(S=\overset{C}{\underset{i=1}{\sum }}𝕀(X_{pip}=0)\overset{P}{\underset{j=p+1}{\sum }}X_{p+1ij}\right),\;\\ S & ∼\mathrm{Bin}\left[(P-p)\left(C-\overset{C}{\underset{i=1}{\sum }}X_{pip}\right),\Phi \left\{\frac{Z_{p|X_{p}}-\eta }{\gamma (1-p/P)}\right\}\right].\; \end{array}$$

To understand this formulae, note that $C-\sum_{i=1}^{C}X_{pip}$ is the number of clusters in the control condition after time period *p*. Thus $\left(P-p)(C-\sum_{i=1}^{C}X_{pip}\right)$ is the number of cluster‐periods for which the roll‐out could be modified. Similarly, $\sum_{i=1}^{C}𝕀(X_{pip}=0)\sum_{j=p+1}^{P}X_{p+1ij}$ is the number of the modifiable cluster‐periods matrix $X_{p+1}$ spends in the intervention condition. The form for the success probability, $\Phi [(Z_{p|X_{p}}-\eta )/\{\gamma (1-p/P)\}]$, is chosen to provide the sought after qualities of the function b(·) and to provide flexibility such that a search can be conducted for an RA design that has desirable operating characteristics. First, Φ(·) is used to map the continuous Wald test statistic to [0,1], enabling its value to serve as a probability that controls the speed of the roll‐out conditional on the interim effectiveness. In addition, η∈ℝ is a parameter that can be chosen to influence the value of $b(X_{p+1})$; larger values of η result in smaller values of Φ(·), favoring designs slowing the roll‐out of the intervention. Similarly, parameter γ>0 influences how extreme the values of Φ(·) are, with larger γ shifting the success probability toward 0.5, which should translate to a more balanced intervention roll‐out. Finally, the denominator includes the factor 1−p/P to scale the success probabilities, allowing them to be more extreme for larger *p* (ie, when more information is available to base the decision upon). This form for b(·) is also discussed further in the Supplementary Material.

The above fully describes the proposed framework for incorporating RA intervention allocation in an SW‐CRT, with the final analysis conducted here analogously to a conventional SW‐CRT by assessing whether $Z_{P|X_{P}}>\Phi^{-1}(1-\alpha )$. We comment in the discussion on potential alternatives to this rejection rule that may be useful in practice. An algorithm on the conduct of an RA SW‐CRT is provided in the Supplementary Material.

### 2.3 Simulation study

We assess the performance of the proposed framework through an extensive simulation study that considers three trial design scenarios (TDSs). Each TDS assumes the following model for data generation and analysis^14^, ^17^, ^18^

$$Y_{ijk}=\beta_{j}+\theta X_{ij}+c_{i}+\pi_{ij}+s_{ik}+\epsilon_{ijk}.$$

Here, $Y_{ijk}$ is the response from individual k=1,…,m, in cluster i=1,…,C, in period j=1,…,P, $\beta_{j}$ is a fixed effect for time period *j*, $c_{i}∼N(0,\sigma_{c}^{2})$ is a random cluster effect, $\pi_{ij}∼N(0,\sigma_{\pi }^{2})$ is a random cluster‐period effect, $s_{ik}∼N(0,\sigma_{s}^{2})$ is a random individual effect, and $\epsilon_{ijk}∼N(0,\sigma_{\epsilon }^{2})$ is the residual error. Thus, in this case, $\theta =(\beta_{1},\ldots ,\beta_{P},\theta )$. Primary results for TDS1 are presented here, where TDS2 is also used to provide a simple illustration of the method's use. Additional findings for TDS1 are given in the Supplementary Material, where the results for TDS2 and TDS3 are also presented.

TDS1 is a cross‐sectional SW‐CRT ($\sigma_{s}^{2}=0$) that has been considered previously.^19^, ^20^, ^21^ It is based on the average characteristics of SW‐CRTs according to Grayling et al,^22^ setting C=20 and P=9. In *X*, three clusters switch to the intervention in each of time periods 2 to 5, and two clusters switch in each of time periods 6 to 9. To give a larger value for the intra‐cluster correlation than TDS2, it has $\sigma_{c}^{2}=1/9$ and $\sigma_{e}^{2}=1$. Additionally, α=0.05, β=0.2, and δ=0.24. Using the sample size calculation method from Hussey and Hughes^11^ (ie, $\sigma_{\pi }^{2}=0$), m=7 is chosen. For the RA designs, we consider conducting a single interim analyses after time period {3}, {4}, or {5}, and conducting two interim analyses after time periods {3,6}.

TDS2 is a cross‐sectional SW‐CRT ($\sigma_{s}^{2}=0$) based upon the trial presented in Bashour et al;^23^ a study assessing the effect of training doctors in communication skills on women's satisfaction with doctor‐woman relationship during labor and delivery. In this case, C=4 and P=5, with *X* switching one cluster to the intervention in each of time periods 2 to 5. The final analysis estimated that $\sigma_{c}^{2}=0.02$ and $\sigma_{e}^{2}=0.51$. We use these values in all simulations. Following the approach of Hussey and Hughes^11^ ($\sigma_{\pi }^{2}=0$), for these variance components the trial would have required 70 patients per cluster‐period for its desired type‐I error‐rate of 5% and its desired type‐II error‐rate of 10% when θ=0.2. Thus, we fix m=70, α=0.05, β=0.1, and δ=0.2. We consider conducting interim analyses after time periods {3} and {2,3,4}.

TDS3 is a closed‐cohort SW‐CRT scenario, based on the “Girls on the Go!” program to improve self‐esteem in young women in Australia,^24^ following the calculation in Hooper et al.^14^ Thus, we consider a case where C=12 and P=4, with *X* switching four clusters to the intervention in time periods 2 to 4. Measurements from m=10 individuals are assumed to be collected in each cluster and the primary outcome measure (Rosenberg Self‐esteem Scale) is assumed to have $\sigma_{c}^{2}=7.425$, $\sigma_{\pi }^{2}=0.825$, $\sigma_{s}^{2}=11.725$, and $\sigma_{\epsilon }^{2}=5.025$. The conventional design achieves β=0.2 for δ=2 with α=0.025. We consider conducting interim analyses after time periods {2} and {2,3}.

In all three TDSs, we consider performance when θ∈{−δ,−0.5δ,…,2δ}, w∈{1/1000,1/4,1/3,1/2,2/3,3/4,999/1000}, η∈{−1,0,1,2,3,4}, and γ∈{1,2.5,5}. These values were chosen by factoring in what was computationally feasible and through an initial grid search to identify a range for the parameters beyond which the operating characteristics did not appear to vary substantially. In all cases, we place no restrictions on the $X_{X_{p}}$ beyond those required (ie, we always set $X_{X_{p}}{=M}_{X_{p}}$).

For each combination of design parameters, 100 000 replicate simulations are used to estimate several key quantities. These are

- The empirical rejection probability (ERP) for $H_{0}$, with the values for θ=0 and θ=δ referred to as the empirical type‐I error‐rate and power.
- The empirical average, standard deviation, and probability mass function of the proportion of cluster‐periods spent in the intervention condition. We refer to the average and standard deviations of this quantity for brevity as the EACP and ESDCP, respectively. The EACP and ESDCP together evaluate patient benefit, for example, larger (smaller) values of the EACP are desired for larger (smaller) treatment effects, while we would likely always prefer small ESDCP. Note that when evaluating these quantities, one must account for the fact that the choice of $X_{p_{1}}$ imparts particular minimal and maximal values for the time spendable in the intervention condition; these will be indicated on all relevant plots.
- The empirical average value of $X_{P}$, denoted ${\bar{X}}_{P}$.
- The empirical bias (EB) and root‐mean‐square error (ERMSE) of the final point estimate of θ, ${\hat{\theta }}_{p|X}$. Previous work for individually randomized trials has explored the negative impacts of RA design on point estimation when it is performed in a manner that does not take in to account the interim analyses.^25^

Code to reproduce our results is available from https://github.com/mjg211/article_code.

## 3 RESULTS

### 3.1 Illustrative description: Trial design scenario 2

To make the proposed methodology more tangible, we illustrate its application to TDS2, where the low number of clusters (C=4) and time periods (P=5) makes the possible allocation matrices limited. As discussed, Bashour et al^23^ utilized the following allocation matrix

$$X=\left(\begin{array}{ccccc} 0 & 1 & 1 & 1 & 1\\ 0 & 0 & 1 & 1 & 1\\ 0 & 0 & 0 & 1 & 1\\ 0 & 0 & 0 & 0 & 1 \end{array}\right).$$

Suppose that there was concern around use of this allocation matrix, such that RA design was to be utilized. In practice, this could happen for one of numerous reasons, though principally it may often be because investigators wish to provide a larger number of participants with the intervention if it is effective (this is often especially true for disease settings in which the condition under investigation can be particularly harmful), or because downsides (eg, cost or harm/safety concerns) mean that they would want to limit roll‐out if the intervention was ineffective. As discussed, the first step is then to specify the time periods after which interim analyses will be conducted. As a basic example, we suppose that this is after period {3}, such that $X_{1}=X_{2}=X_{3}=X$.

Thus, the RA trial would proceed by conducting periods 1 to 3 and then computing $Z_{3|X_{3}}$ using the interim data. Placing no constraints on $X_{X_{3}}$ beyond those required, we would have

$$\begin{array}{ll} X_{4} & =X_{5}{\in X}_{X_{3}}=\{M_{1},M_{2},\ldots ,M_{6}\},\;\\ M_{1} & =\left(\begin{array}{ccccc} 0 & 1 & 1 & 1 & 1\\ 0 & 0 & 1 & 1 & 1\\ 0 & 0 & 0 & 0 & 0\\ 0 & 0 & 0 & 0 & 0 \end{array}\right),\;M_{2}=\left(\begin{array}{ccccc} 0 & 1 & 1 & 1 & 1\\ 0 & 0 & 1 & 1 & 1\\ 0 & 0 & 0 & 0 & 1\\ 0 & 0 & 0 & 0 & 0 \end{array}\right),\;M_{3}=\left(\begin{array}{ccccc} 0 & 1 & 1 & 1 & 1\\ 0 & 0 & 1 & 1 & 1\\ 0 & 0 & 0 & 0 & 1\\ 0 & 0 & 0 & 0 & 1 \end{array}\right),\;\\ M_{4} & =\left(\begin{array}{ccccc} 0 & 1 & 1 & 1 & 1\\ 0 & 0 & 1 & 1 & 1\\ 0 & 0 & 0 & 1 & 1\\ 0 & 0 & 0 & 0 & 0 \end{array}\right),\;M_{5}=\left(\begin{array}{ccccc} 0 & 1 & 1 & 1 & 1\\ 0 & 0 & 1 & 1 & 1\\ 0 & 0 & 0 & 1 & 1\\ 0 & 0 & 0 & 0 & 1 \end{array}\right),\;M_{6}=\left(\begin{array}{ccccc} 0 & 1 & 1 & 1 & 1\\ 0 & 0 & 1 & 1 & 1\\ 0 & 0 & 0 & 1 & 1\\ 0 & 0 & 0 & 1 & 1 \end{array}\right).\; \end{array}$$

For the assumed Hussey and Hughes model and variance parameters ($\sigma_{s}^{2}=0$, $\sigma_{\pi }^{2}=0$, $\sigma_{c}^{2}=0.02$, $\sigma_{e}^{2}=0.51$), it can be shown that

$$I_{5|M_{1}}\approx 188.5,\;I_{5|M_{2}}\approx 224.5,\;I_{5|M_{3}}\approx 204.7,\;I_{5|M_{4}}\approx 222.2,\;I_{5|M_{5}}\approx 215.2,\;I_{5|M_{6}}\approx 169.8.$$

To determine the choice of the interim specified allocation matrix, $X_{4}$, we then must also calculate the values of the b(·). Suppose that η=0 and γ=2.5, and as an example assume $Z_{3|X_{3}}=1$. Using our definition of *S*, we have

$$S∼\mathrm{Bin}\left[(5-3)\left(4-\overset{4}{\underset{i=1}{\sum }}X_{3i3}\right),\Phi \left\{\frac{1-0}{2.5(1-3/5)}\right\}\right].$$

That is, S∼Bin{4,Φ(1)}. Using our definition of b(·), this gives

$$\begin{array}{ll} b(M_{1}) & =\mathbb{P}(S=0)\approx 0.001,\;b(M_{2})=\mathbb{P}(S=1)\approx 0.013,\;b(M_{3})=\mathbb{P}(S=2)\approx 0.107,\;\\ b(M_{4}) & =\mathbb{P}(S=2)\approx 0.107,\;b(M_{5})=\mathbb{P}(S=3)\approx 0.378,\;b(M_{6})=\mathbb{P}(S=4)\approx 0.501.\; \end{array}$$

Finally, supposing that w=0.5, we can use the above to show that

$$s(M_{1})\approx 0.420,\;s(M_{2})\approx 0.513,\;s(M_{3})\approx 0.563,\;s(M_{4})\approx 0.601,\;s(M_{5})\approx 0.856,\;s(M_{6})\approx 0.878.$$

Thus, $M_{6}$ is the matrix that maximizes s(·), and so we set $X_{4}=X_{5}=M_{6}$ and conduct periods 4 to 5 of the trial using its roll‐out. At the end of the study, we have that the proportion of cluster‐periods spent in the intervention condition is 55%, while the value of $Z_{5|X_{5}}$ determines whether $H_{0}$ is rejected.

This is of course description of one possible realization of carrying out an RA trial. Our key concerns revolve around what the expected performance of this approach would look like, in terms of our metrics the ERP, EACP, ESDCP, EB, and ERMSE. We present these evaluations in the Supplementary Materials, where we also consider conducting interim analyses after time periods {2,3,4}.

### 3.2 Trial design scenario 1

Switching to TDS1, we commence our investigation of the expected performance of RA procedures. Note that additional results for TDS1 are given in the Supplementary Materials.

#### 3.2.1 Operating characteristics for η=0 and γ=2.5

**FIGURE 1:**
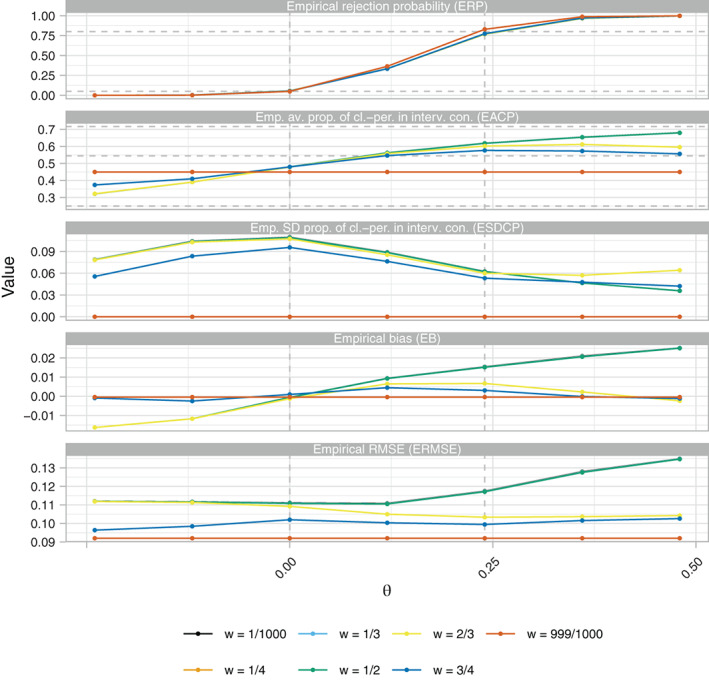
The empirical rejection probability (ERP) and empirical average proportion of cluster‐periods spent in the intervention condition (EACP), as functions of *w* and θ, of the response adaptive (RA) stepped‐wedge cluster randomized trial (SW‐CRT) designs with η=0, γ=2.5, and $\left\{p_{1},\ldots ,p_{L}\}=\{3,6\right\}$, in trial design scenario 1 (TDS1). The dashed lines in the ERP plot indicate the desired type‐I and type‐II error‐rates. In the EACP plot they indicate the minimal, initially planned, and maximal values of the EACP based on $X=X_{p_{1}}$

Figure 1 displays the ERP, EACP, ESDCP, EB, and ERMSE of several RA SW‐CRT designs as a function of *w* and θ when $\left\{p_{1},\ldots ,p_{L}\}=\{3,6\right\}$. As an example, results for η=0 and γ=2.5 are displayed. Increasing the value of *w* results in increased power as would be expected, though the difference between the power curves for w≠999/1000 is small. For w=999/1000 the priority given to maximizing power results in an empirical power of 83.0%; above the desired level. The EB is observed to be small, relative to the value of θ, regardless of the value of *w*. However, only for w=999/1000 is the final point estimate unbiased. A slightly larger impact on the ERMSE is seen for w≠999/1000 compared to the impact on the EB, though arguably performance is surprisingly strong considering w=999/1000 results in the design that minimizes the ERMSE.

For w∈{1/1000,1/4,1/3,1/2} the EACP is almost identical and increases monotonically in θ. For w=999/1000 the EACP is constant, indicative of the same design being chosen to maximize power no matter the value of θ. For w∈{2/3,3/4}, the EACP initially increases in θ, but the competing factors in s(·) eventually result in decreases for larger θ. The ESDCP is maximized for each *w* when θ=0. The precise values of the ESDCP are arguably small when considered in unison with the EACP. For example, for w=1/2, the ESDCP for θ=δ together with the corresponding EACP indicates that in the majority of cases we would expect the roll‐out to be sped up, as would be desired.

**FIGURE 2:**
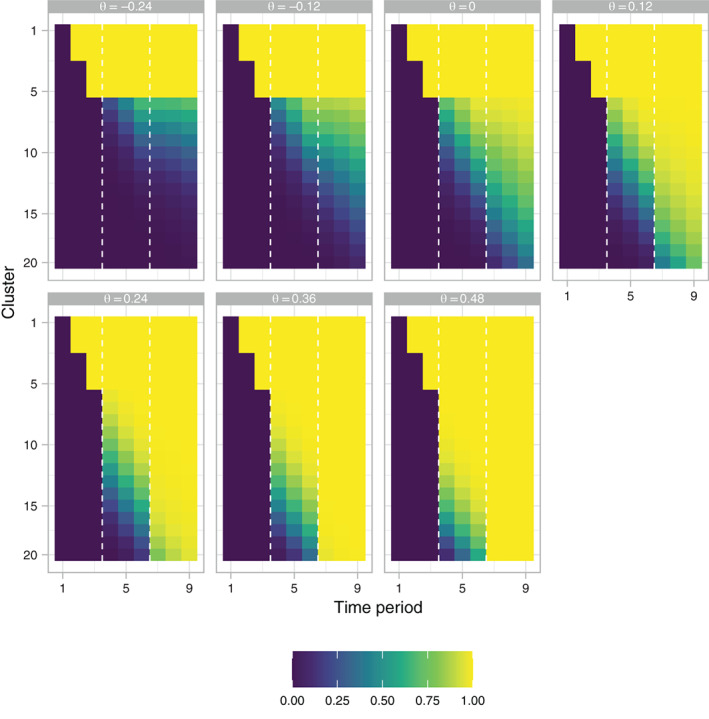
The empirical average final allocation matrix (${\bar{X}}_{P}$), as a function of θ, of the response adaptive (RA) stepped‐wedge cluster randomized trial (SW‐CRT) design with η=0, γ=2.5, w=1/2, and $\left\{p_{1},\ldots ,p_{L}\}=\{3,6\right\}$, in trial design scenario 1 (TDS1). The dashed lines indicate the timing of the interim analyses

**FIGURE 3:**
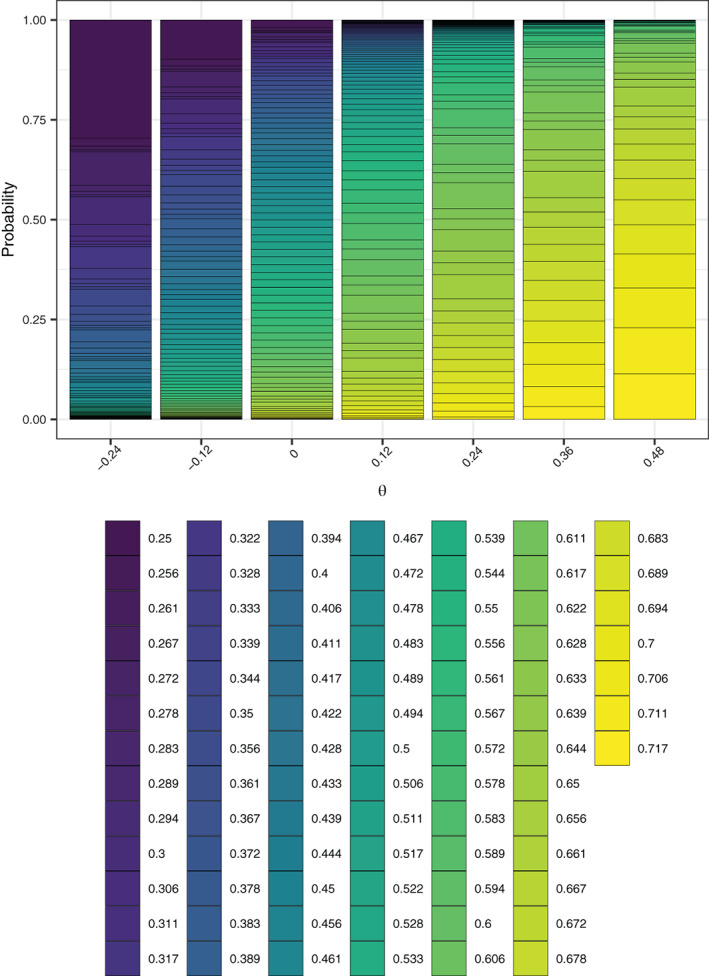
The empirical probability mass function of the proportion of cluster‐periods spent in the intervention condition, as a function of θ, of the response adaptive (RA) stepped‐wedge cluster randomized trial (SW‐CRT) design with η=0, γ=2.5, w=1/2, and $\left\{p_{1},\ldots ,p_{L}\}=\{3,6\right\}$, in trial design scenario 1 (TDS1)

For w=1/2, the EACP ranges from 32.2% when θ=−δ to 67.9% when θ=2δ. Under the null and alternative hypotheses the corresponding figures are 48.0% and 61.8%, respectively. This contrasts to 54.4% for the fixed (initially‐planned) design and 45.0% for w=999/1000. Figure 2 displays this pictorially, giving the average value of $X_{P}$ when w=1/2. Similarly, Figure 3 presents the probability mass function of the proportion of time spent in the intervention condition. The probability of making an “incorrect” decision (eg, decreasing the roll‐out speed for a large true intervention effect) is evidently small when the absolute value of θ is large. A potential downside of RA design is observed for, for example, θ=0, where the precise variation in the final proportion of participants who received the intervention is evident, when in this case we may prefer some (fixed) value close to 50%. The empirical type‐I error‐rate and power are 5.6% and 76.8%, respectively, in this case.

#### 3.2.2 Operating characteristics as a function of η and γ

**FIGURE 4:**
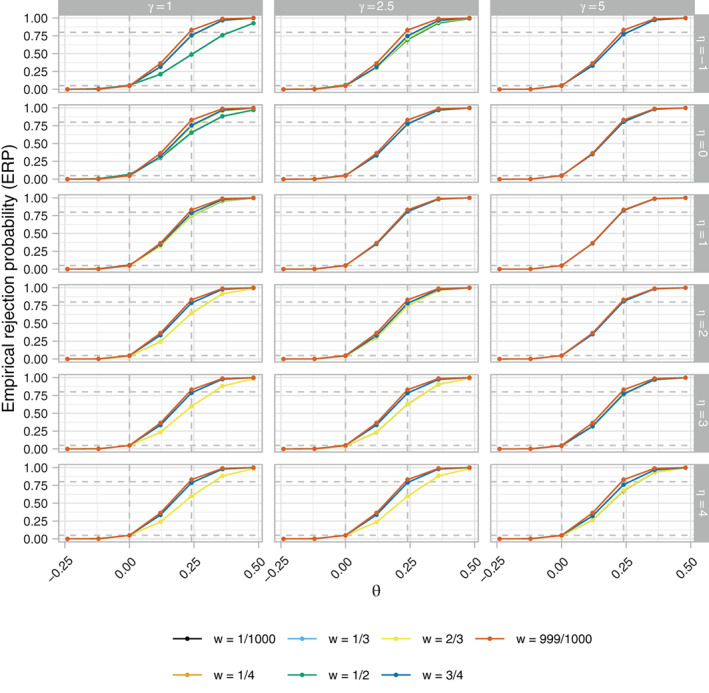
The empirical rejection probability (ERP), as a function of *w* and θ, of the response adaptive (RA) stepped‐wedge cluster randomized trial (SW‐CRT) designs with $\left\{p_{1},\ldots ,p_{L}\}=\{3,6\right\}$ for different combinations of η and γ, in trial design scenario 1 (TDS1). The dashed lines indicate the desired type‐I and type‐II error‐rates

**FIGURE 5:**
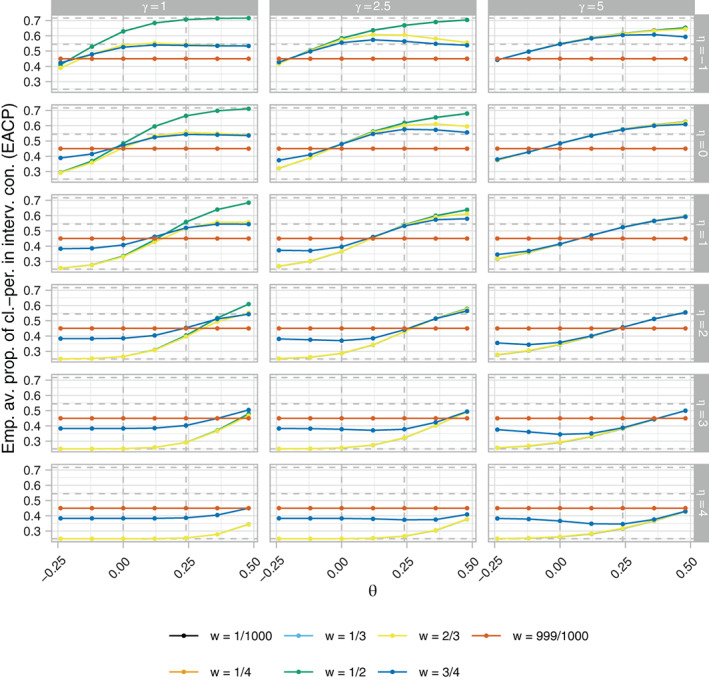
The empirical average proportion of cluster‐periods spent in the intervention condition (EACP), as a function of *w* and θ, of the response adaptive (RA) stepped‐wedge cluster randomized trial (SW‐CRT) designs with $\left\{p_{1},\ldots ,p_{L}\}=\{3,6\right\}$ for different combinations of η and γ, in trial design scenario 1 (TDS1). The dashed lines indicate the minimal, initially planned, and maximal values of the EACP based on $X=X_{p_{1}}$

**FIGURE 6:**
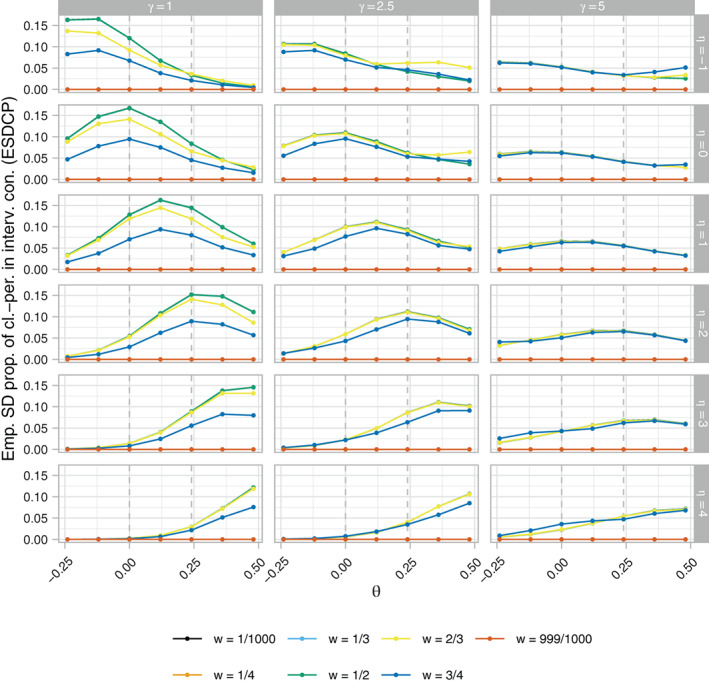
The empirical standard deviation of the proportion of cluster‐periods spent in the intervention condition (ESDCP), as a function of *w* and θ, of the response adaptive (RA) stepped‐wedge cluster randomized trial (SW‐CRT) designs with $\left\{p_{1},\ldots ,p_{L}\}=\{3,6\right\}$ for different combinations of η and γ, in trial design scenario 1 (TDS1)

Figures 4, 5, 6, respectively, present the ERP, EACP, and ESDCP of the RA SW‐CRT designs, as functions of *w* and θ, for different combinations of η and γ. Corresponding presentations for the EB and ERMSE are given in the Supplementary Materials. For several combinations of η and γ the power curves are similar across θ for multiple values of *w*, attaining approximately the desired type‐I error‐rate and power. Larger differences are observed in some instances, however, typically for more extreme values of η and γ. For fixed η, increasing γ generally results in an increase in power. This should be anticipated as larger γ promotes a more steady roll‐out, which will often correspond to allocation matrices with power closer to the desired level. Similarly, for fixed γ, increasing η initially results in power gains, but in many cases eventually leads to power loss as the procedure recommends those designs that terminate the roll‐out.

These comments match the plots in Figure 5, with for example those designs with η=4 having very low values for the EACP. Furthermore, it can be seen that for w=0.5, for example, increasing γ generally results in a flattening of the EACP curve as a function of θ, as the more extreme roll‐outs attain lower values for b(·).

Qualitatively different findings are observed in Figure 6, however. For γ=5, the ESDCP is similar for all w≠999/10 000 and varies little as a function of θ or η. This is a consequence of large γ placing a high preference on approximately 50% of cluster‐periods being spent in the intervention condition. For γ=2.5, the ESDCP again varies little across values of w≠999/1000, but now varies substantially as a function of θ and η. The maximal values of the ESDCP for γ=2.5 can often be considered low when viewed in combination with the corresponding EACP. This is not always the case for γ=1, though, where for certain *w* (eg, w=1/2) the ESDCP indicates variation in the roll‐out speed such that performance may often be considered poor (eg, an increase in roll‐out from that initially planned when θ<0).

#### 3.2.3 Operating characteristics as a function of $\left\{p_{1},\ldots ,p_{L}\right\}$

**FIGURE 7:**
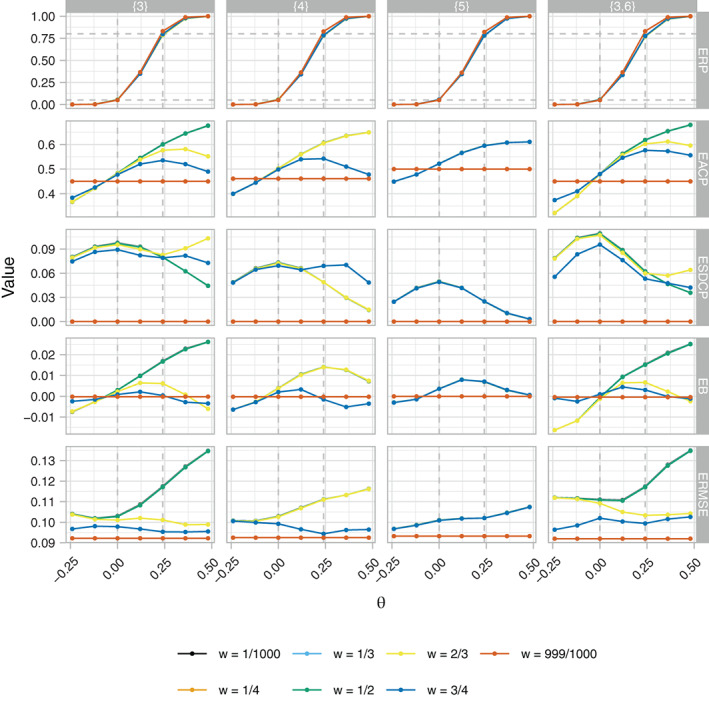
The empirical rejection probability (ERP), average proportion of cluster‐periods spent in the intervention condition (EACP), standard deviation of cluster‐periods spent in the intervention condition (EACP), bias (EB), and root‐mean‐square error (RMSE), as functions of *w* and θ, of the response adaptive (RA) stepped‐wedge cluster randomized trial (SW‐CRT) designs with η=0 and γ=2.5, for different values of $\left\{p_{1},\ldots ,p_{L}\right\}$, in trial design scenario 1 (TDS1). The dashed lines in the ERP plot indicate the desired type‐I and type‐II error‐rates. In the EACP plot they indicate the minimal, initially planned, and maximal values of the EACP based on $X=X_{p_{1}}$

Figure 7 presents the ERP, EACP, ESDCP, EB, and ERMSE of the RA SW‐CRT designs as functions of $\left\{p_{1},\ldots ,p_{L}\right\}$ for η=0 and γ=2.5. It can be seen that there is little evidence changing the timing of a single‐interim analysis from {3} to {5} carries a cost to the ERP. However, delaying the timing of a single‐interim analysis inhibits the ability of the RA designs to offer a wider range of values for the EACP. In addition, the EACP curves are similar for an increasing number of values of *w* the later the timing of the interim analysis; this is a consequence of both the decrease in possible allocation matrices that can be chosen from and increasing precision in the value of the $Z_{p|X_{p}}$. Another consequence of this is that the ESDCP is smaller when the first interim analysis is conducted later in the trial, though the difference is only pronounced when {3} is contrasted with {5}.

There is a larger cost to the EB for certain *w* when an interim analysis is conducted earlier in the trial (ie, for {3} and {3,6}). However, the actual cost remains small relative to the value of θ. Similar statements are true for the ERMSE.

Compared to the designs with $\left\{p_{1},\ldots ,p_{L}\}=\{3\right\}$, those with $\left\{p_{1},\ldots ,p_{L}\}=\{3,6\right\}$ incur a small cost to their empirical power. However, this is counterbalanced by them achieving a wider range of values for the EACP when w≠999/1000.

## 4 DISCUSSION

Concerns have been expressed over the pre‐trial decision of SW‐CRTs to provide the intervention to all clusters. It may therefore be advantageous to allow the intervention roll‐out to be sped‐up or slowed‐down according to information accrued during the trial. Accordingly, we have presented methodology through which this could be achieved. Our presented framework is flexible, allowing the design to be constructed to balance considerations on power and ethical allocation. Furthermore, while we focused on data analysis via a linear mixed model, the framework is dependent only on the availability of an interim estimate of effectiveness. It could therefore be readily modified, for example, for a generalized estimating equation analysis of noncontinuous data (see, eg, Li et al^26^ or Ford and Westgate^27^ for relevant methodology in the nonadaptive setting).

To examine the performance of the framework, we conducted a large simulation study. From this, several important observations can be made. Principally, it should not be assumed that any choice of values for η and γ will provide desirable operating characteristics. However, in all three TDSs it was possible to find combinations that provided monotonically increasing values for the EACP without major inflation of the type‐I or type‐II error‐rate (eg, in TDS1 w=0.5, η=0, and γ=2.5 provided such performance). Our recommendation would be therefore that these should be chosen carefully in practice, via a comprehensive simulation study. Nonetheless, it was clear that some small impact to the error‐rates may be unavoidable if one is to attain a design with large variation in the EACP as a function of the intervention effect. The small power loss may be resolved in practice through a small increase to the sample size computed for the corresponding fixed sample design.

Addressing the observed type‐I error‐rate inflation poses an interesting question as to whether methodology developed to help attain a desired test size in small fixed‐sample CRTs could find additional utility in adaptive design scenarios. Such methodology has been a topic of much recent interest. For example, Leyrat et al^28^ considered the performance of numerous analysis methods (eg, weighted and unweighted cluster‐level analyses, mixed‐effects models with different degree‐of‐freedom corrections, GEEs with and without a small‐sample correction) for parallel‐group CRTs with a low number of clusters and a continuous outcome. Scott et al^29^ and Ford and Westgate^27^ examined possible correction methods for GEE analyses of SW‐CRTs. Thompson et al^30^ recently provided an extensive comparison of such small‐sample correction methods and degrees‐of‐freedom corrections for GEE analyses of binary data in SW‐CRTs, with Ren et al^31^ previously conducting similar work in a continuous outcome setting. While the type‐I error‐rate inflation observed in our RA SW‐CRTs was often small, if addressing such inflation was a priority then it is likely such methodology would offer a potential, albeit heuristic, solution. We note though that simulation would be required to ascertain which approach may be most appropriate, as there is no guarantee results in a fixed‐sample setting would be directly transferable to RA design.

The advantageous performance of the RA designs is particularly noteworthy since only designs with a small number of interim analyses were evaluated. One may have anticipated that more interim analyses may have been required to realize benefits of RA randomization. A small number of interim analyses may be important in practice to reduce their logistical burden. It is also more computationally feasible to evaluate performance in this setting and it may be anticipated to be associated with smaller inflation of the type‐I error‐rate as the data is assessed less frequently.

The findings should perhaps not be surprising, given the large number of alternatives to the initially planned allocation matrix that will have similar power means there are often other choices available that can at least slightly alter the intervention's allocation without compromising on power. Furthermore, the timing of the first interim analysis provides a natural and effective means of protecting a degree of data accrual in the intervention and control conditions; this is similar to the typical use of a burn‐in period for RA designs in individually randomized trials. The timing of the first interim analysis can also be seen to be crucial to enabling a wider range of EACP values to be possible; the one‐directional switching of SW‐CRTs means that RA design can offer far less later in a trial as the number of possible allocation schemes decreases. However, we note that even when only small changes in the EACP are achieved this can have a substantial impact on the number of patients who receive the intervention, depending on the value of the total trial sample size. Finally, there was substantial degeneracy in the operating characteristics for different values of *w*, particularly in those designs where the first interim analysis was timed later in the trial. In practice, only a small number of values for *w* may need to be considered, and in many instances the choice of w=1/2 worked well.

It is important to acknowledge some limitations to our work. First, while our investigations reveal limited impact on the bias in the final point estimate from utilizing an RA design, we have not addressed potentially important characteristics of the asymptotic properties of the estimator (eg, consistency) or provided a way to remove any bias. We leave extending bias removal methodology for individually randomized RA trials^25^ to this SW‐CRT setting for future work. Nor have we examined the potential implications of model mis‐specification on the utility of the proposed RA procedure. Recent work has, as discussed, highlighted a range of possible analysis methods that make, for example, differing assumptions on the correlation between the outcome measurements.^15^ It is possible that model mis‐specification may impact RA design more starkly than it does a fixed‐sample SW‐CRT. While there is potential in an adaptive setting to adaptively update the chosen analysis model, which could help overcome such a problem, we have not addressed this here and no work to date is available to indicate whether this may be a fruitful approach. Each of these considerations may, in particular, impact the applicability of the proposed methodology in a regulated trial setting.

In addition, while we have provided examples on cross‐sectional and closed‐cohort designs, we have not directly addressed RA design of an open‐cohort SW‐CRT. Our methods could be applied to an open‐cohort SW‐CRT under the assumption of some particular sampling scheme.^32^ However, the degree to which the assumed sampling scheme is “correct” would then likely influence the usefulness of RA design. Consequently, the approach to RA design for an open‐cohort trial should arguably also attempt to re‐estimate the “true” sampling scheme at the time of the interim analyses, which we have not presented methodology for here. Regardless of the approach used, thorough investigation of the utility of RA design for open‐cohort designs would then require simulations to be performed under a variety of open‐cohort sampling schemes, with exploration of the impact of these being correctly or incorrectly specified.

The practical considerations in relation to utilizing an RA SW‐CRT design should also be recognized. Many of these are similar to those described in Grayling et al^22^ within the context of early termination in SW‐CRTs. In particular, while the time period structure of SW‐CRTs may appear to lend itself naturally to sequential methodology, the interim analyses would be highly dependent upon the efficient collection, storage, and processing of data. Arguably the largest issue for RA intervention allocation, though, is whether logistical or practical constraints may inhibit the ability to modify the roll‐out. While a roll‐out could likely often be slowed down, it may be challenging to speed it up. Furthermore, allowing slow‐down could be argued to disincentivize cluster participation.

Limitations above aside, our results indicate RA allocation of the intervention could potentially provide notable advantages. It is important to discuss therefore when such a design may be useful. In practice, RA design could be deemed useful in a wide variety of settings, where this conclusion may not be immediately apparent; a number of SW‐CRTs have now incorporated interim evaluations of efficacy/futility,^33^, ^34^, ^35^, ^36^, ^37^, ^38^, ^39^ and it is not always clear from published information why such adaptations were included. However, we note that RA could be particularly helpful when either the intervention itself or its evaluation is highly expensive, such that investigators would not wish to complete the roll‐out unless it was effective. Most likely though, in our opinion, it may be helpful when there are substantial patient benefit considerations associated with the intervention, potentially in combination with notable safety concerns. This could be true, for example, of vaccine development during an epidemic.

Following the Ebola outbreak of 2014 to 2015, many authors discussed the applicability of SW‐CRTs to evaluating vaccine effectiveness.^40^, ^41^, ^42^, ^43^, ^44^, ^45^, ^46^, ^47^, ^48^, ^49^, ^50^, ^51^, ^52^, ^53^, ^54^, ^55^, ^56^ Importantly, this setting was one in which a short time was expected between intervention delivery and outcome accrual,^52^ which is important for RA design. Furthermore, there was little data available about the safety or immunogenicity of the vaccine candidates.^44^ Consequently, proposals to use SW‐CRT designs were not based on preliminary data that the vaccine may do more good than harm and the safety considerations arguably amplify the need to prevent roll‐out if a vaccine was ineffective. Indeed, van der Tweel and van der Graaf^56^ noted their concerns that many clusters could end up being exposed to an inferior treatment, while Doussau and Grady^44^ went as far as to state that interim analyses may be needed. It also seems reasonable to assume such a setting would be one in which resources would be made available to carry out interim adaptations efficiently, owing to the degree of the public health emergency.

The main limitation to utilizing an RA SW‐CRT design of the type considered here would be the aforementioned resource availability to speed up a vaccines roll‐out. It would be important to ensure that at the epidemic's onset manufacturing processes were put in place to scale up the development of any vaccine for which preliminary evidence of effectiveness was obtained. The other principal limitation, discussed extensively by Bellan et al,^40^ is that SW‐CRTs are not well equipped to handling spatiotemporal variation in a virus outbreak; much power can often be gained from prioritizing where to administer a vaccine. This issue cannot be handled by the type of RA SW‐CRT proposed here. However, it indicates that an adaptive incomplete‐block CRT may be worth considering in future studies of the efficient evaluation of a vaccine. Such a design could add new clusters during the course of the study, constraining the randomization to prioritize the speed of its delivery to specific hot‐spots. We note it may also be important to consider incorporating other types of adaptation in to this type of design, including stopping rules^19^, ^22^ or sample size re‐estimation,^21^ in order to identify the most suitable CRT design.

In conclusion, when it is feasible to modify an intervention's allocation in an SW‐CRT, RA design theory could help improve the trial's patient benefit characteristics. This may be particularly relevant to settings in which the intervention is expensive or could be associated with significant harm.

## Supporting information


**Data S1** Supplementary material

## Data Availability

Code to reproduce our results is available from https://github.com/mjg211/article_code.
