# Supplementary material for: Response adaptive intervention allocation in stepped‐wedge cluster randomized trials
Source: Stat Med. 2022 Jan 21;41(6):1081–99. doi: 10.1002/sim.9317 (PMC7612601; doi:10.1002/sim.9317)
Supplement: Supplementary file 1 — Data S1 Supplementary material [file SIM-41-1081-s001.pdf]

Supplementary Materials to 'Response adaptive intervention allocation in stepped-wedge cluster randomised trials'

Alternative choice of $b(\cdot)$

To choose only between terminating, immediately completing, or continuing with the currently planned roll-out, the following function could be used

$$b(X_{p+1}) = \mathbb{I}(Z_{p|X_p} > u)\prod_{i=1}^{C}\prod_{j=p+1}^{P}\mathbb{I}(X_{p+1ij} = 1) + \mathbb{I}(Z_{p|X_p} \leq l)\prod_{i=1}^{C}\prod_{j=p+1}^{P}\mathbb{I}(X_{pip} = 0)\mathbb{I}(X_{p+1ij} = 0)$$
$$+\mathbb{I}(l < Z_{p|X_p} \leq u)\prod_{i=1}^{C}\prod_{j=p+1}^{P}\mathbb{I}(X_{p+1ij} = X_{pij}) \ .$$

This is the formal approach to stating that X_{p+1} will be rewarded a score of either zero or one. A score of one is rewarded if (i) $Z_{p|X_p} > u$ and X_{p+1} is such that every cluster receives the intervention in the remaining time periods, (ii) $Z_{p|X_p} \leq l$ and X_{p+1} is such that no additional clusters receive the intervention in the remaining time periods, or (iii) if $l < Z_{p|X_p} \leq u$ and $X_{p+1} = X_p$. The score is zero otherwise. Such an approach is similar in its underlying decision making to incorporating early stopping for efficacy and futility, without actual early termination of the study.

Choice of $b(\cdot)$ in the main manuscript

To expand further on the intuition behind the choice of $b(\cdot)$ in the main manuscript, consider it again

$$b(X_{p+1}) = \mathbb{P}\left(S = \sum_{i=1}^{C}\mathbb{I}(X_{pip} = 0)\sum_{j=p+1}^{P}X_{p+1ij}\right),$$
$$S \sim Bin\left[(P-p)\left(C - \sum_{i=1}^{C}X_{pip}\right), \Phi\left\{\frac{Z_{p|X_p} - \eta}{\gamma(1-p/P)}\right\}\right].$$

Thus this function advises on the number of modifiable cluster-periods that should be spent in the intervention condition through the densities of an appropriate binomial distribution. Specifically, the number of trials of this binomial variable is the number of modifiable cluster-periods, $(P-p)(C - \sum_{i=1}^{C}X_{pip})$. The probability any given cluster-period should be spent in the intervention condition is $\Phi[(Z_{p|X_p} - \eta)/\{\gamma(1-p/P)\}]$, which utilises the current evidence of effectiveness ($Z_{p|X_p}$) in a flexible way (through the inclusion of parameters η and γ). For any X_{p+1}, its number of modifiable cluster-periods spent in the intervention condition, $\sum_{i=1}^{C}\mathbb{I}(X_{pip} = 0)\sum_{j=p+1}^{P}X_{p+1ij}$, is computed. Then, e.g., if $\Phi[(Z_{p|X_p} - \eta)/\{\gamma(1-p/P)\}]$ is large and $\sum_{i=1}^{C}\mathbb{I}(X_{pip} = 0)\sum_{j=p+1}^{P}X_{p+1ij}$ is small relative to $(P -$

$p)\left(C-\sum_{i=1}^{C}X_{pip}\right)$, X_{p+1} would receive a low score owing to the low value of the computed mass of the given binomial distribution, $\mathbb{P}\left(S=\sum_{i=1}^{C}\mathbb{I}(X_{pip}=0)\sum_{j=p+1}^{P}X_{p+1ij}\right)$. In this way, matrices that spend an appropriate amount of time in the intervention condition according to the interim evidence of effectiveness will be recommended by $b(\cdot)$.

Note that the use of $Z_{p_1|X_{p_1}}$ in the RA rule means it is important at least one cluster receives the intervention in time period p_1 ($\sum_{i=1}^{C}X_{p_1ip_1}>0$), such that θ can be estimated at the first interim analysis. In all considered simulation scenarios we therefore choose values of X_{p_1} such that this is the case.

Algorithm on response adaptive stepped-wedge cluster randomised trial conduct

Algorithmically, the conduct of a RA SW-CRT using the proposed framework is

1. Choose values for C, T, m, X, and $\{p_1,\dots,p_L\}$.
2. Set $p=1$.
3. Conduct period p of the trial, allocating the intervention according to the values X_{pip} for $1\le i\le C$, and accruing m measurements per cluster.
4. Then
   - If $p\in\{p_1,\dots,p_L\}$, set $X_{p+1}=\text{argmax}_{X\in\mathscr{X}_{X_p}}\,s(X)$, $p=p+1$, and return to 3. That is, update the allocation matrix.
   - Else if $p=P$, conclude the trial, rejecting H_0 if $Z_{P|X_P}>\Phi^{-1}(1-\alpha)$. That is, conduct the trial's final analysis.
   - Else set $X_{p+1}=X_p$, $p=p+1$, and return to 3. That is, continue with the same allocation matrix.

Trial Design Scenario 1: Additional results

$\{p_1,\dots,p_L\}=\{3,6\}$

Figures S1-S2 present the EB and ERMSE of the RA designs, as discussed in the main manuscript. Larger values of γ and η result in a trend for reduced bias and RMSE for $w\in\{1/1000,1/4,1/3,1/2,2/3\}$. Results for $w=3/4$ are little influenced by the values of γ and η, while these parameters have no effect for $w=999/1000$. Figure S3 extends Figure 3 by providing results for multiple values of w. Through this, the pattern in which roll-out speeds are increased as w is decreased is evident.

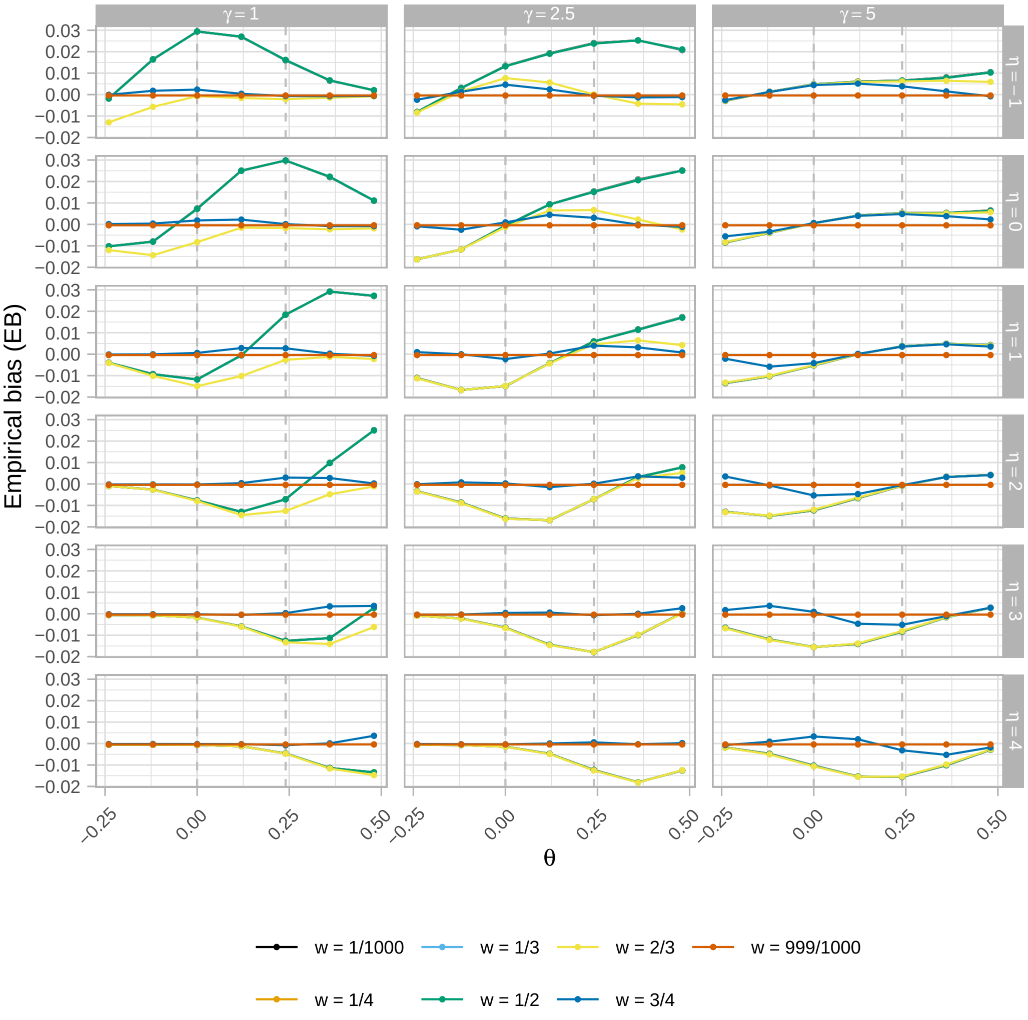

Figure S1: The empirical bias (EB), as a function of w and θ, of the response adaptive (RA) stepped-wedge cluster randomised trial (SW-CRT) designs with $\{p_1, \ldots, p_L\} = \{3, 6\}$ for different combinations of η and γ, in Trial Design Scenario 1 (TDS1).

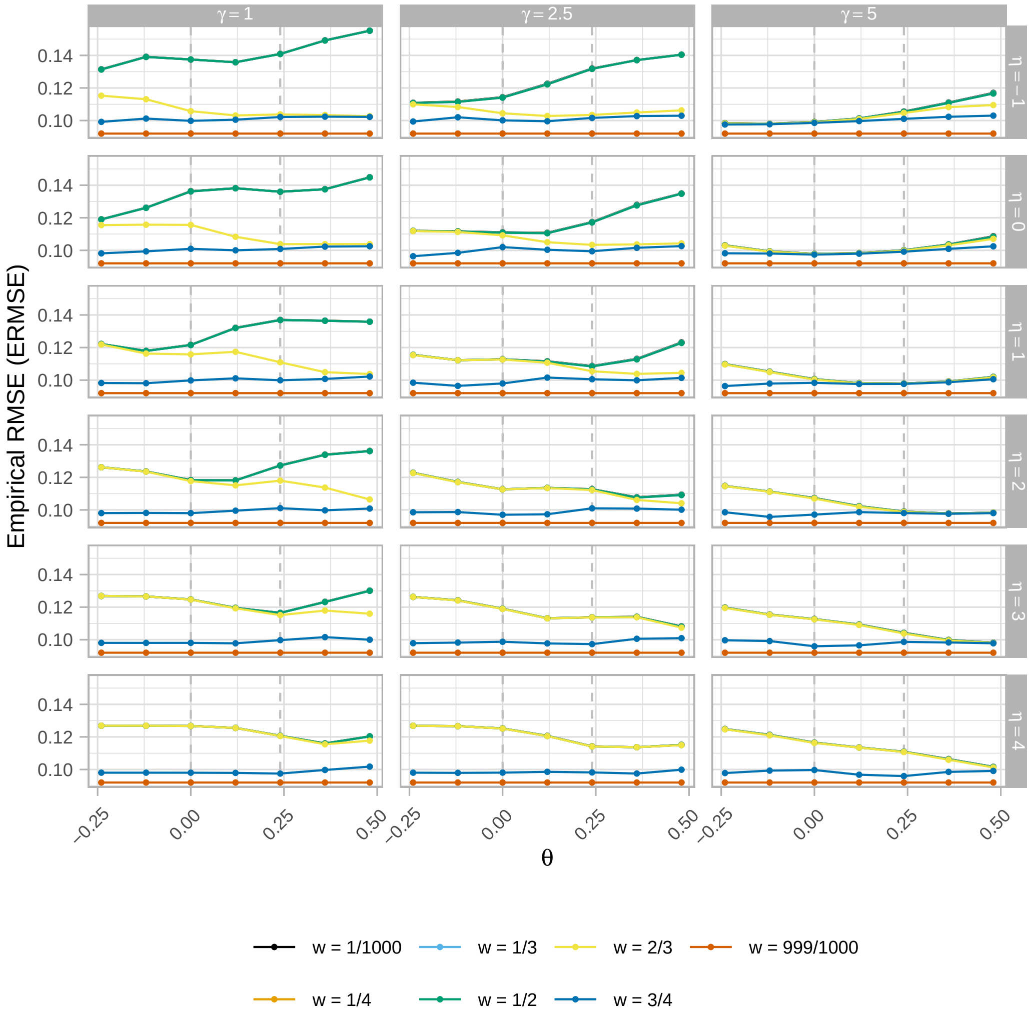

Figure S2: The empirical root-mean-square error (ERMSE), as a function of w and θ, of the response adaptive (RA) stepped-wedge cluster randomised trial (SW-CRT) designs with $\{p_1, \ldots, p_L\} = \{3, 6\}$ for different combinations of η and γ, in Trial Design Scenario 1 (TDS1).

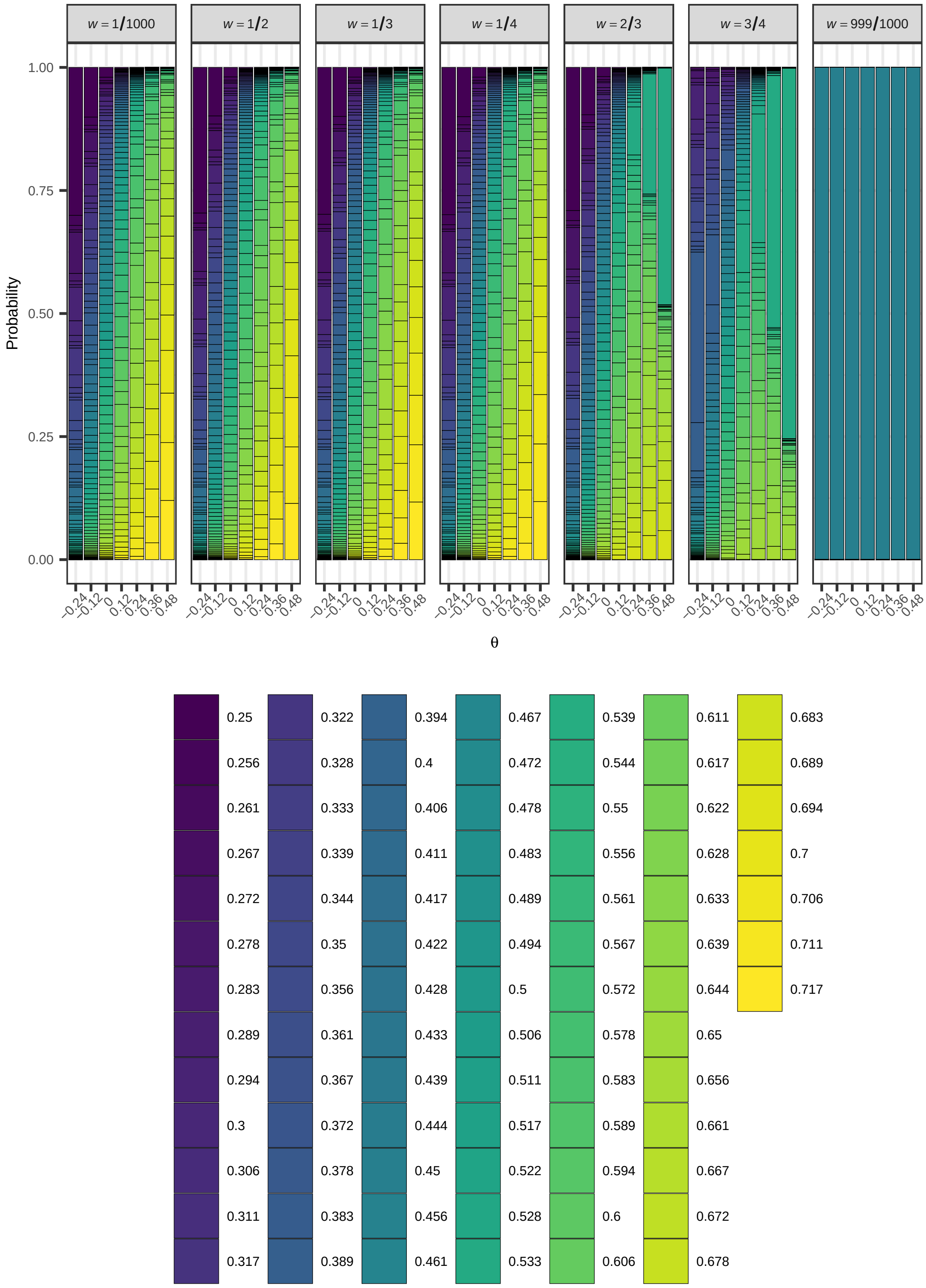

Figure S3: The empirical probability mass function of the proportion of cluster-periods spent in the intervention condition, as a function of θ and w, of the response adaptive (RA) stepped-wedge cluster randomised trial (SW-CRT) design with $\eta = 0$, $\gamma = 2.5$, and $\{p_1, \ldots, p_L\} = \{3, 6\}$, in Trial Design Scenario 1 (TDS1).

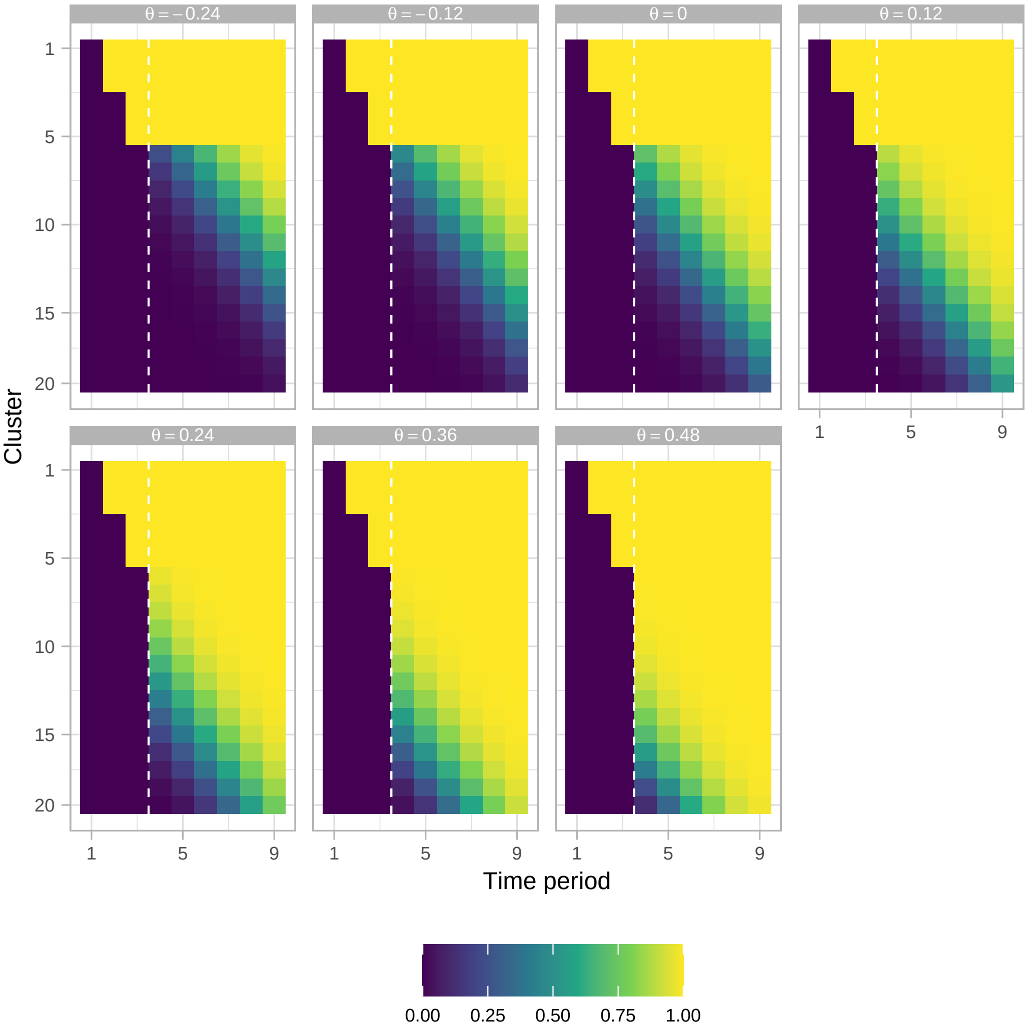

Figure S4: The empirical average final allocation matrix ($\bar{X}_P$), as a function of θ, of the response adaptive (RA) stepped-wedge cluster randomised trial (SW-CRT) design with $\eta = 0$, $\gamma = 2.5$, $w = 1/2$, and $\{p_1, \dots, p_L\} = \{3\}$, in Trial Design Scenario 1 (TDS1). The dashed lines indicate the timing of the interim analyses.

$\{p_1, \dots, p_L\} = \{3\}$

As in the results presented in the main manuscript for $\{p_1, \dots, p_L\} = \{3, 6\}$, those given here for $\{p_1, \dots, p_L\} = \{3\}$ show that there exists combinations of w, η, and γ such that the EACP increases in θ (Figure S6) with minimal impact to the trials error-rates (Figure S5). For example, when $w = 1/2$, $\eta = 0$, and $\gamma = 2.5$, the EACP increases from 36.7% when $\theta = -\delta$ to 67.6% when $\theta = 2\delta$, attaining ERPs when $\theta = 0$ and $\theta = \delta$ of 5.6% and 78.7% respectively.

$\{p_1, \dots, p_L\} = \{4\}$

In timing the single interim analysis after time period 4, Figures S11-S17 show that the key result above of the existence of w, η, and γ that impart desirable EACP properties remains intact. However, later

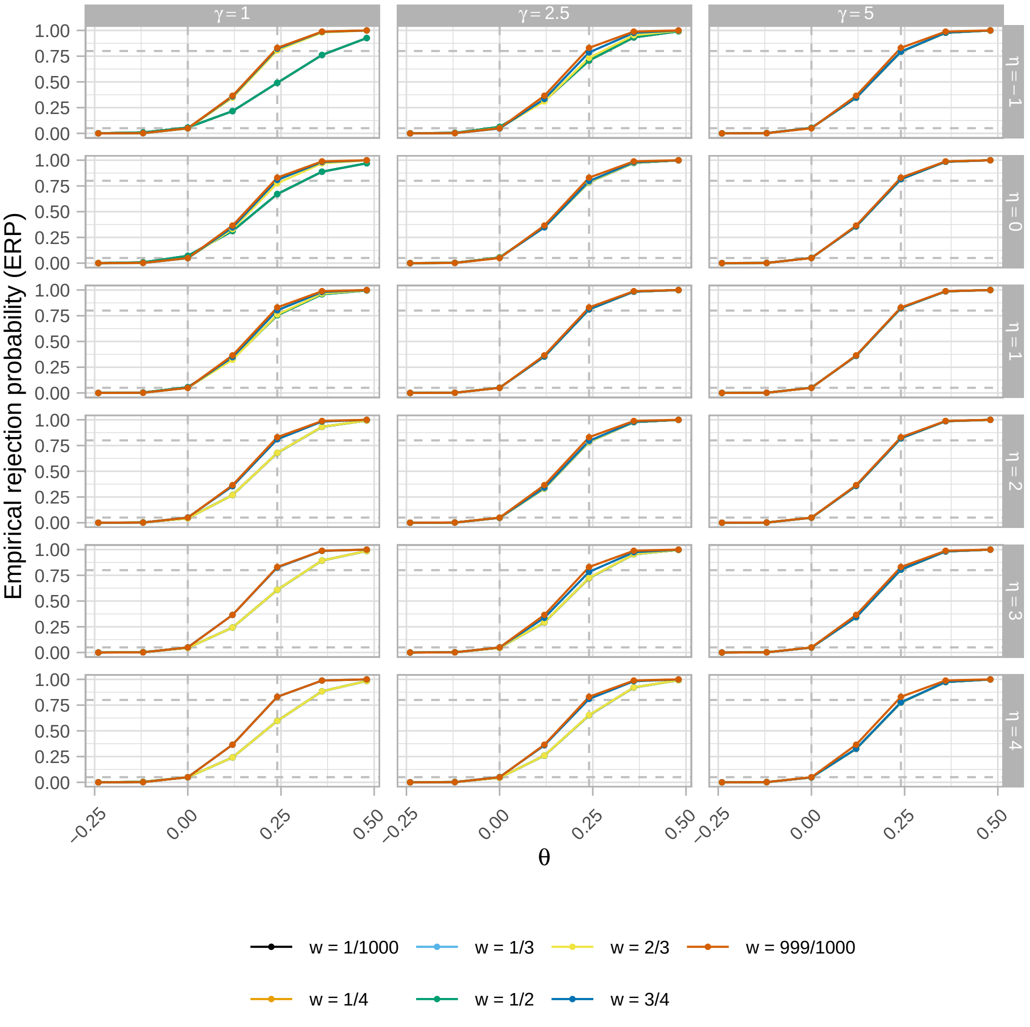

Figure S5: The empirical rejection probability (ERP), as a function of w and θ, of the response adaptive (RA) stepped-wedge cluster randomised trial (SW-CRT) designs with $\{p_1, \dots, p_L\} = \{3\}$ for different combinations of η and γ, in Trial Design Scenario 1 (TDS1). The dashed lines indicate the desired type-I and type-II error-rates.

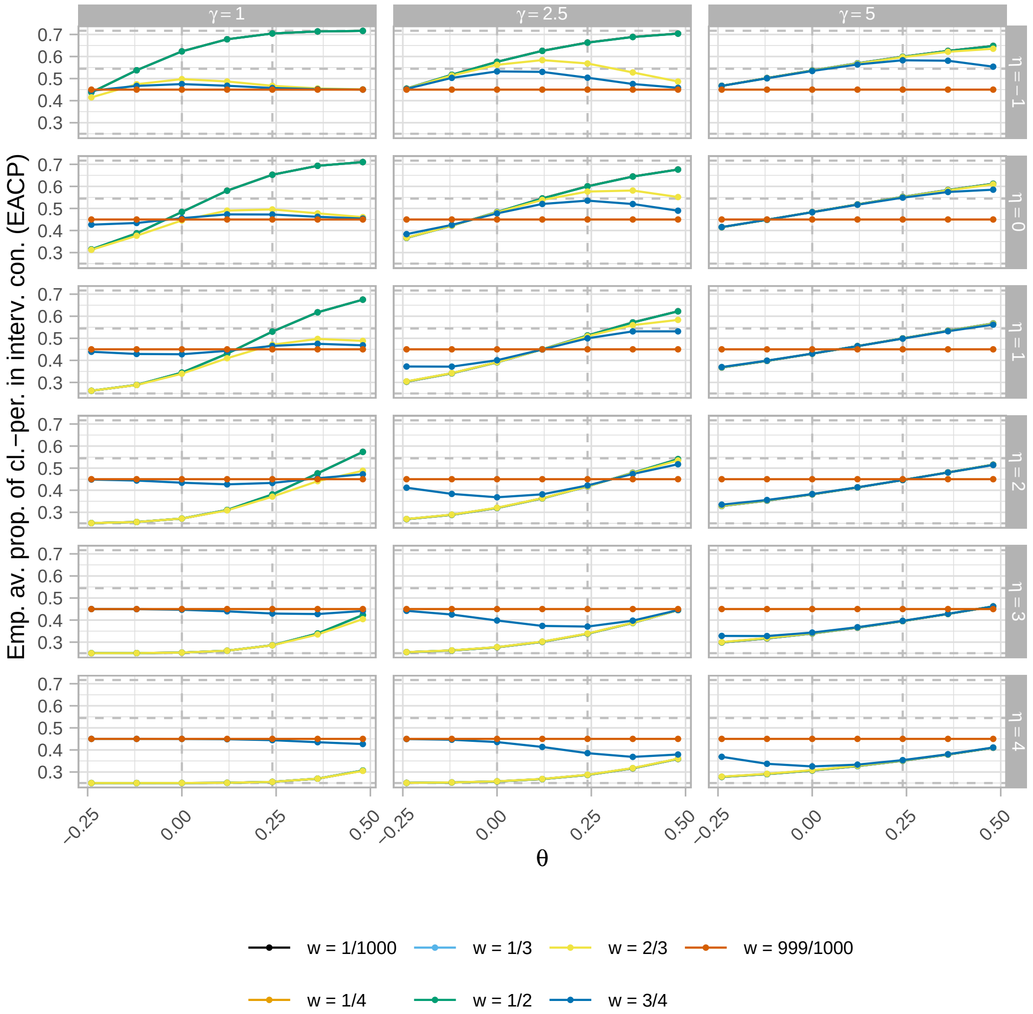

Figure S6: The empirical average proportion of cluster-periods spent in the intervention condition (EACP), as a function of w and θ, of the response adaptive (RA) stepped-wedge cluster randomised trial (SW-CRT) designs with $\{p_1, \ldots, p_L\} = \{3\}$ for different combinations of η and γ, in Trial Design Scenario 1 (TDS1). The dashed lines indicate the minimal, initially planned, and maximal values of the EACP based on $X = X_{p_1}$.

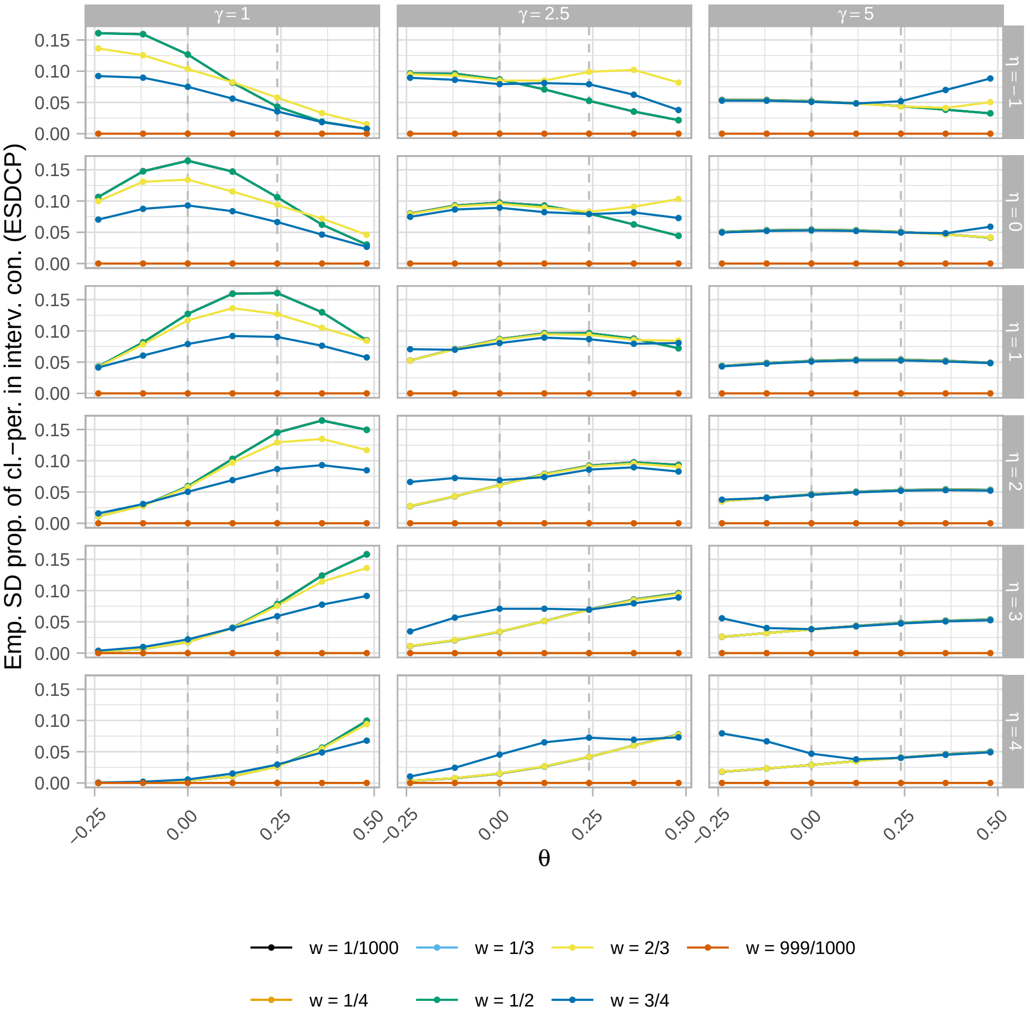

Figure S7: The empirical standard deviation of the proportion of cluster-periods spent in the intervention condition (ESDCP), as a function of w and θ, of the response adaptive (RA) stepped-wedge cluster randomised trial (SW-CRT) designs with $\{p_1, \ldots, p_L\} = \{3\}$ for different combinations of η and γ, in Trial Design Scenario 1 (TDS1).

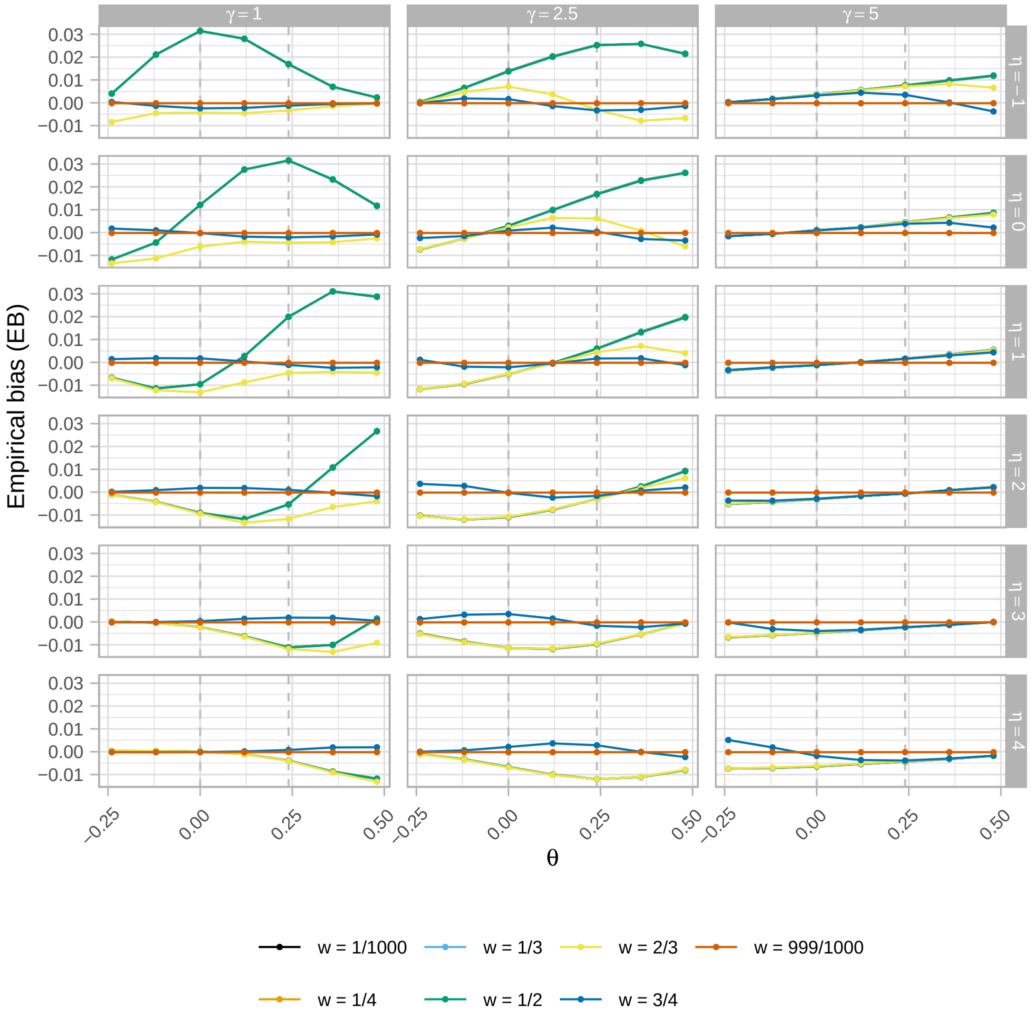

Figure S8: The empirical bias (EB), as a function of w and θ, of the response adaptive (RA) stepped-wedge cluster randomised trial (SW-CRT) designs with $\{p_1, \ldots, p_L\} = \{3\}$ for different combinations of η and γ, in Trial Design Scenario 1 (TDS1).

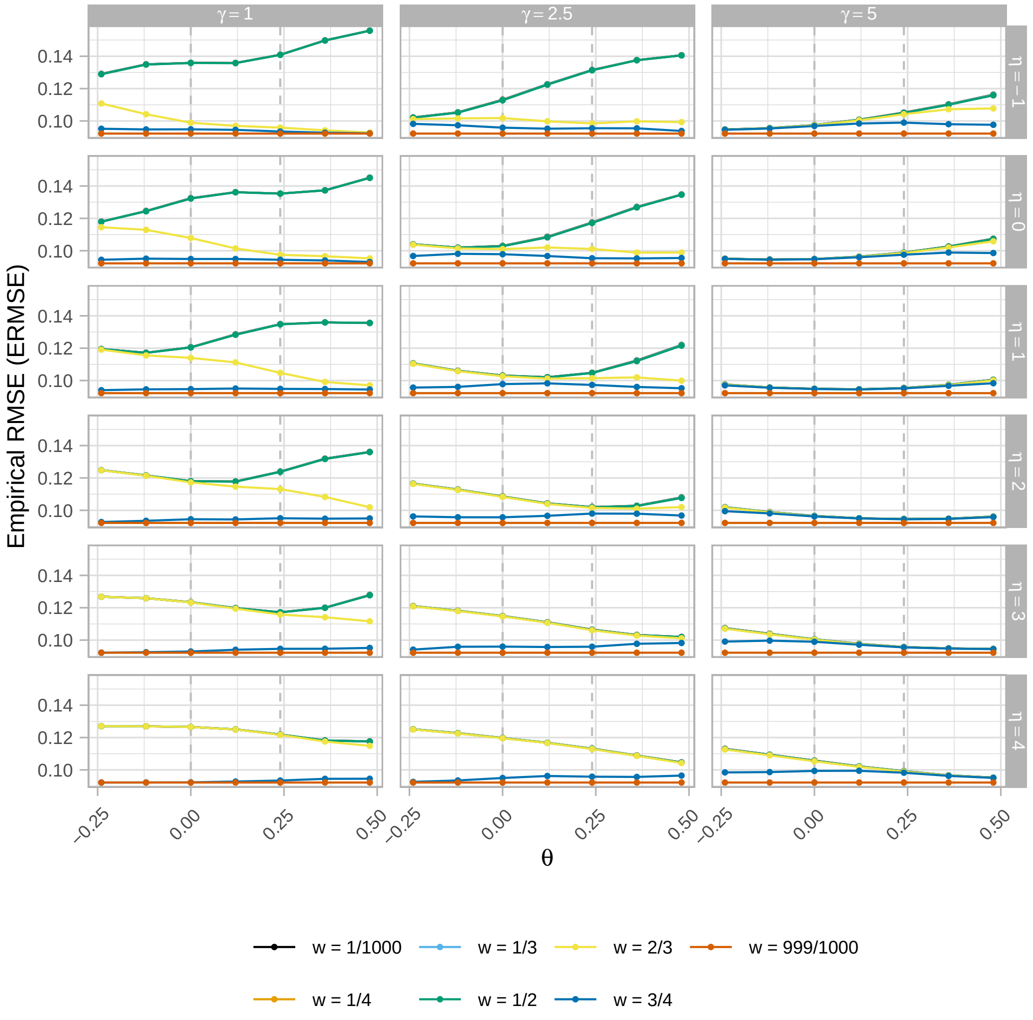

Figure S9: The empirical root-mean-square error (ERMSE), as a function of w and θ, of the response adaptive (RA) stepped-wedge cluster randomised trial (SW-CRT) designs with $\{p_1, \dots, p_L\} = \{3\}$ for different combinations of η and γ, in Trial Design Scenario 1 (TDS1).

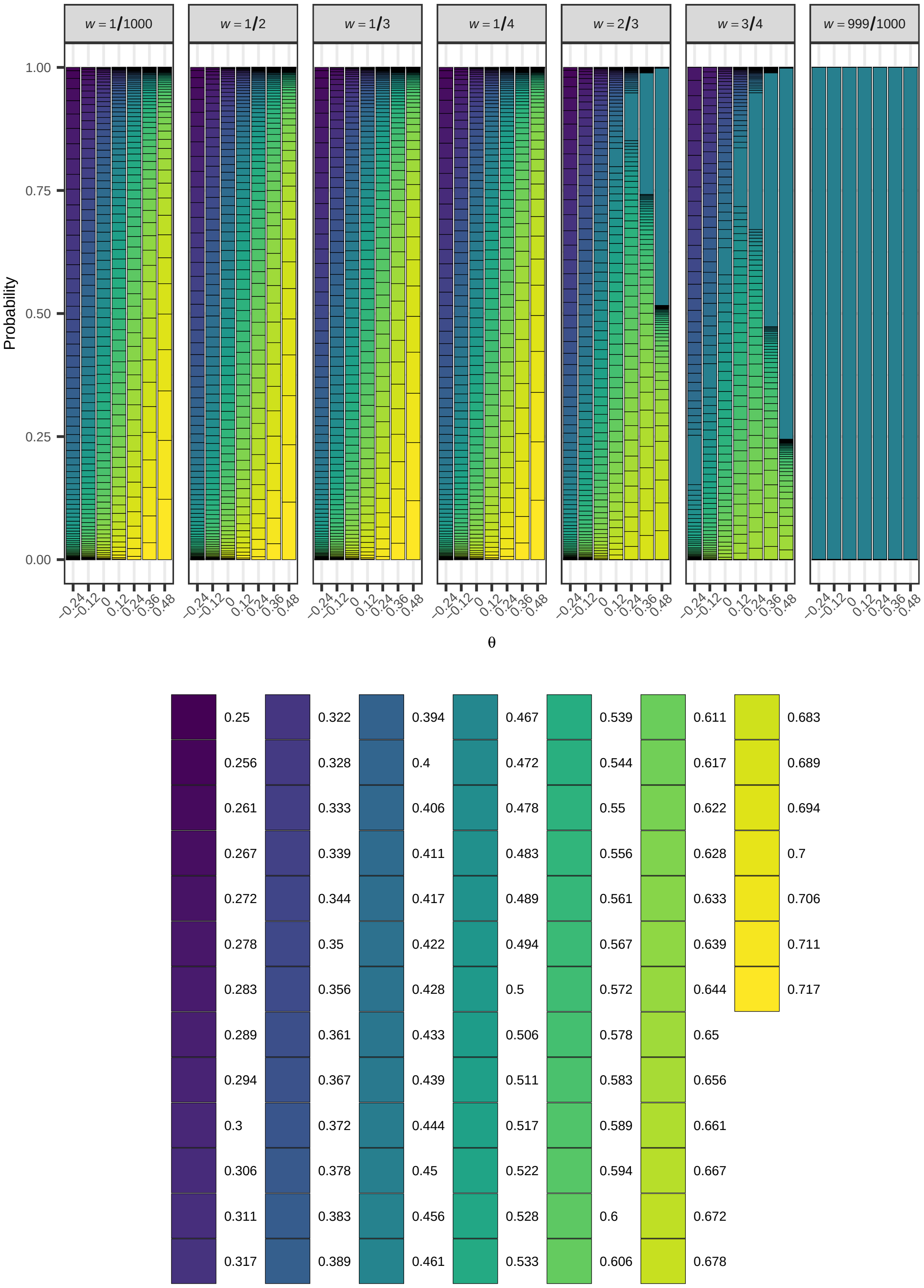

Figure S10: The empirical probability mass function of the proportion of cluster-periods spent in the intervention condition, as a function of θ and w, of the response adaptive (RA) stepped-wedge cluster randomised trial (SW-CRT) design with $\eta = 0$, $\gamma = 2.5$, and $\{p_1, \dots, p_L\} = \{3\}$, in Trial Design Scenario 1 (TDS1).

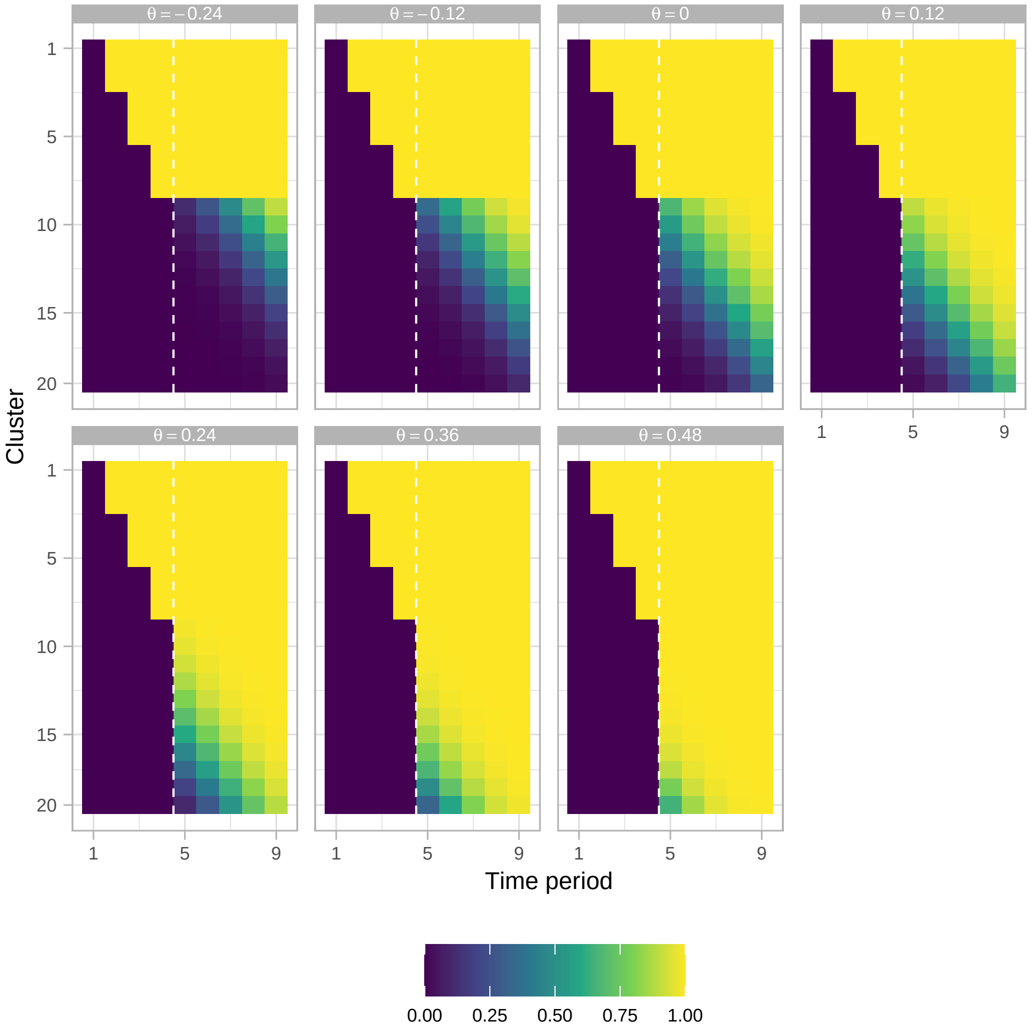

Figure S11: The empirical average final allocation matrix ($\bar{X}_P$), as a function of θ, of the response adaptive (RA) stepped-wedge cluster randomised trial (SW-CRT) design with $\eta = 0$, $\gamma = 2.5$, $w = 1/2$, and $\{p_1, \dots, p_L\} = \{4\}$, in Trial Design Scenario 1 (TDS1). The dashed lines indicate the timing of the interim analyses.

timing of interim analyses reduces the number of possible allocations that can be used in the remaining time period. This results in performance converging for several values of w for given η and γ pairs.

$\{p_1, \dots, p_L\} = \{5\}$

The trend highlighted in the above for $\{p_1, \dots, p_L\} = \{4\}$ becomes more apparent in the case where $\{p_1, \dots, p_L\} = \{5\}$: modifying w typically now results in no change to the performance indicators (Figures S18-S24).

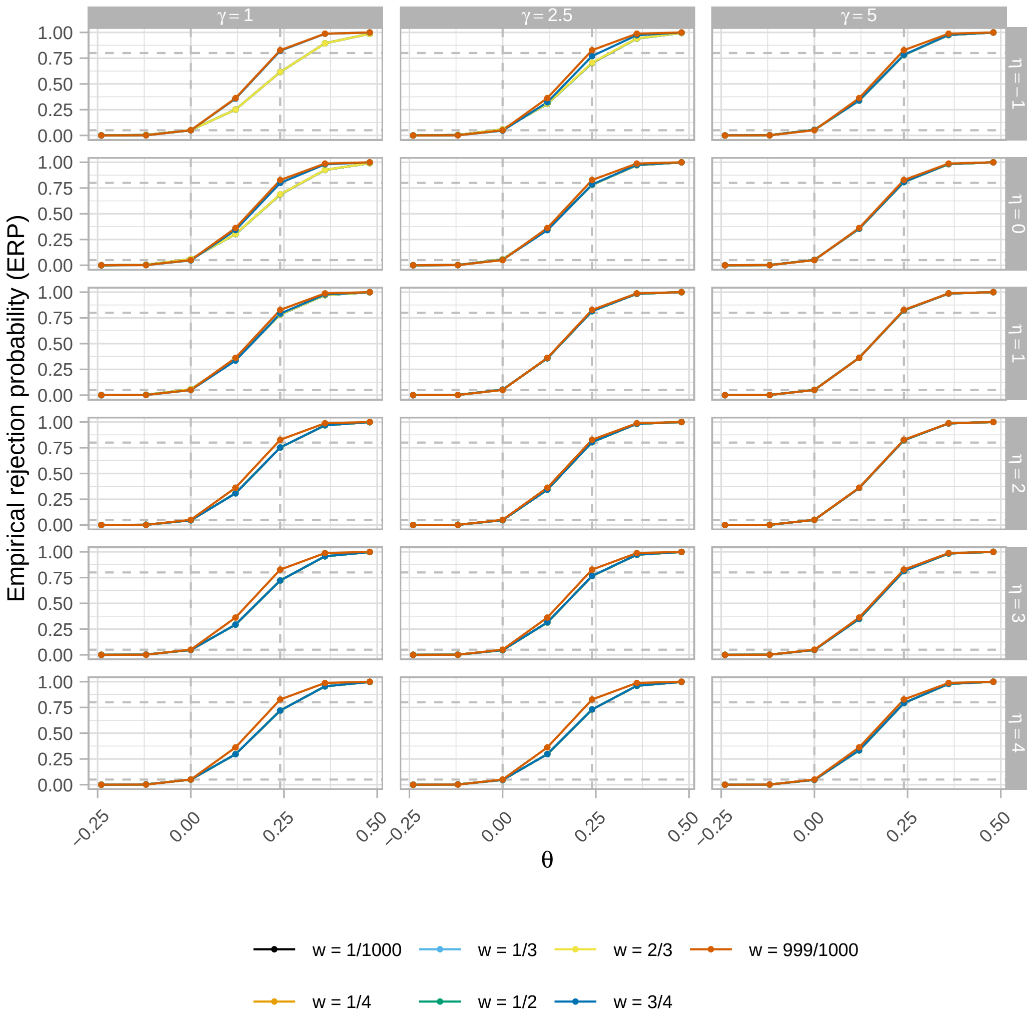

Figure S12: The empirical rejection probability (ERP), as a function of w and θ, of the response adaptive (RA) stepped-wedge cluster randomised trial (SW-CRT) designs with $\{p_1, \dots, p_L\} = \{4\}$ for different combinations of η and γ, in Trial Design Scenario 1 (TDS1). The dashed lines indicate the desired type-I and type-II error-rates.

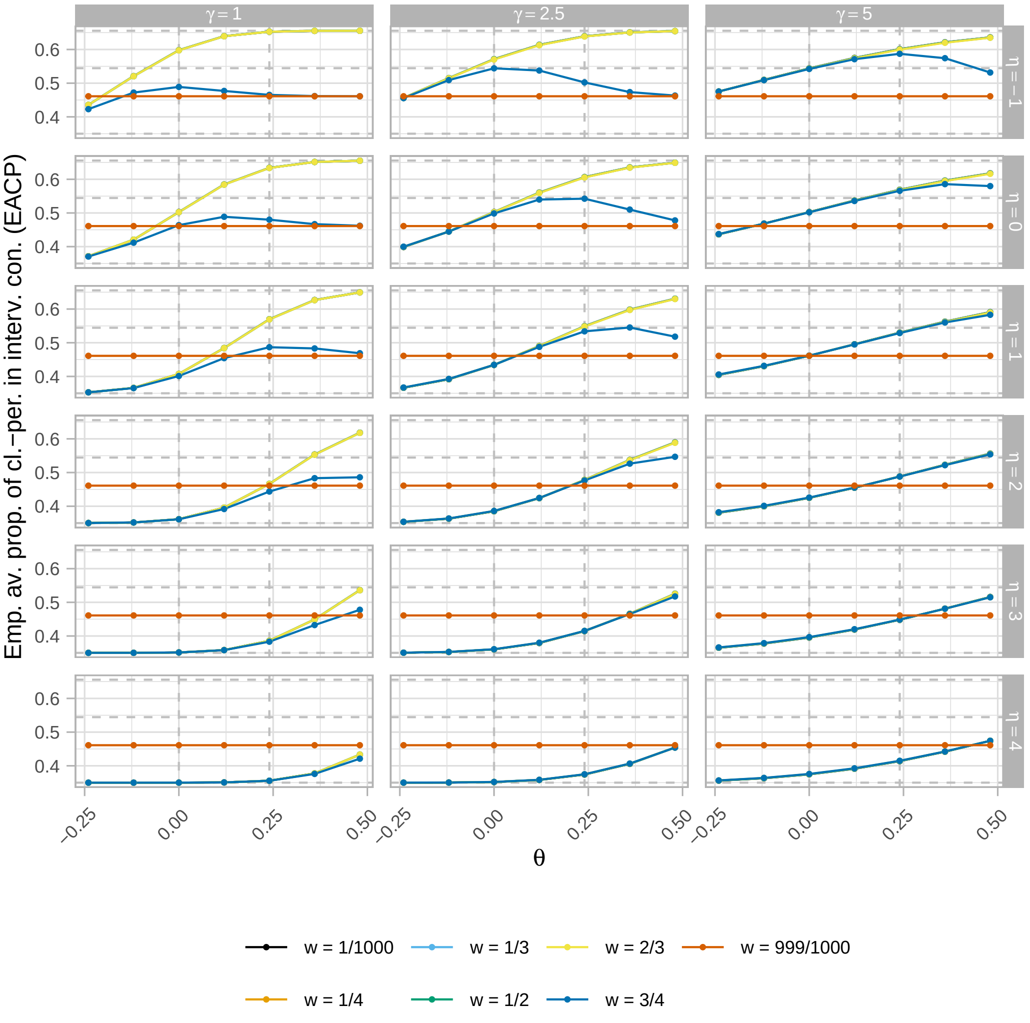

Figure S13: The empirical average proportion of cluster-periods spent in the intervention condition (EACP), as a function of w and θ, of the response adaptive (RA) stepped-wedge cluster randomised trial (SW-CRT) designs with $\{p_1, \ldots, p_L\} = \{4\}$ for different combinations of η and γ, in Trial Design Scenario 1 (TDS1). The dashed lines indicate the minimal, initially planned, and maximal values of the EACP based on $X = X_{p_1}$.

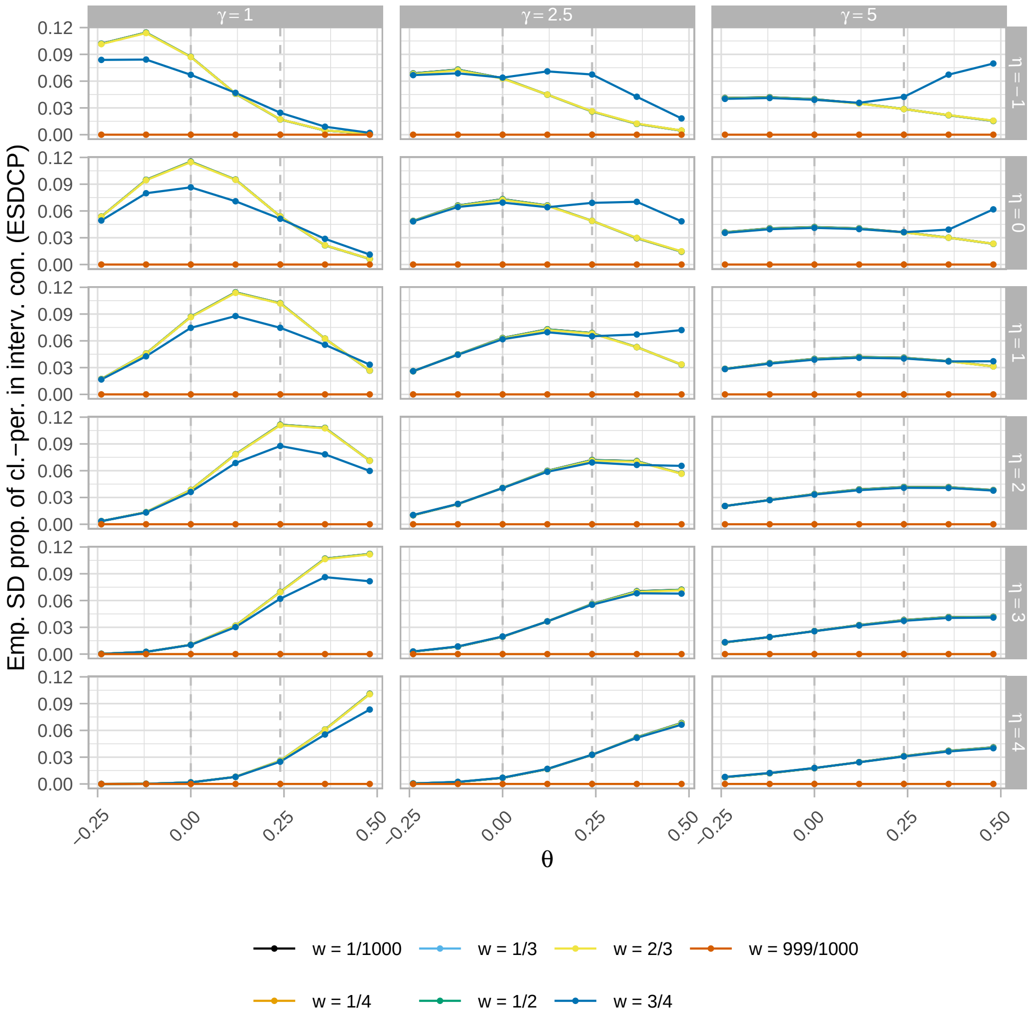

Figure S14: The empirical standard deviation of the proportion of cluster-periods spent in the intervention condition (ESDCP), as a function of w and θ, of the response adaptive (RA) stepped-wedge cluster randomised trial (SW-CRT) designs with $\{p_1, \ldots, p_L\} = \{4\}$ for different combinations of η and γ, in Trial Design Scenario 1 (TDS1).

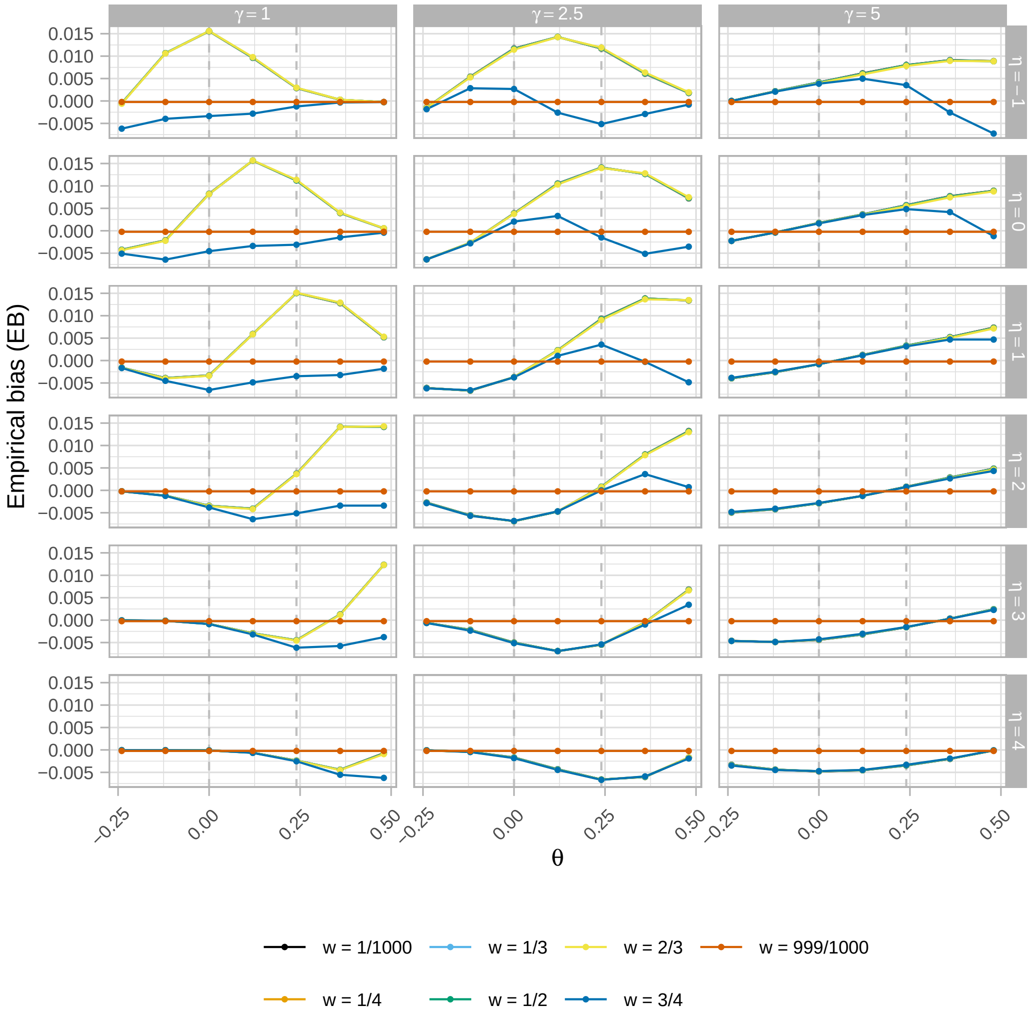

Figure S15: The empirical bias (EB), as a function of w and θ, of the response adaptive (RA) stepped-wedge cluster randomised trial (SW-CRT) designs with $\{p_1, \dots, p_L\} = \{4\}$ for different combinations of η and γ, in Trial Design Scenario 1 (TDS1).

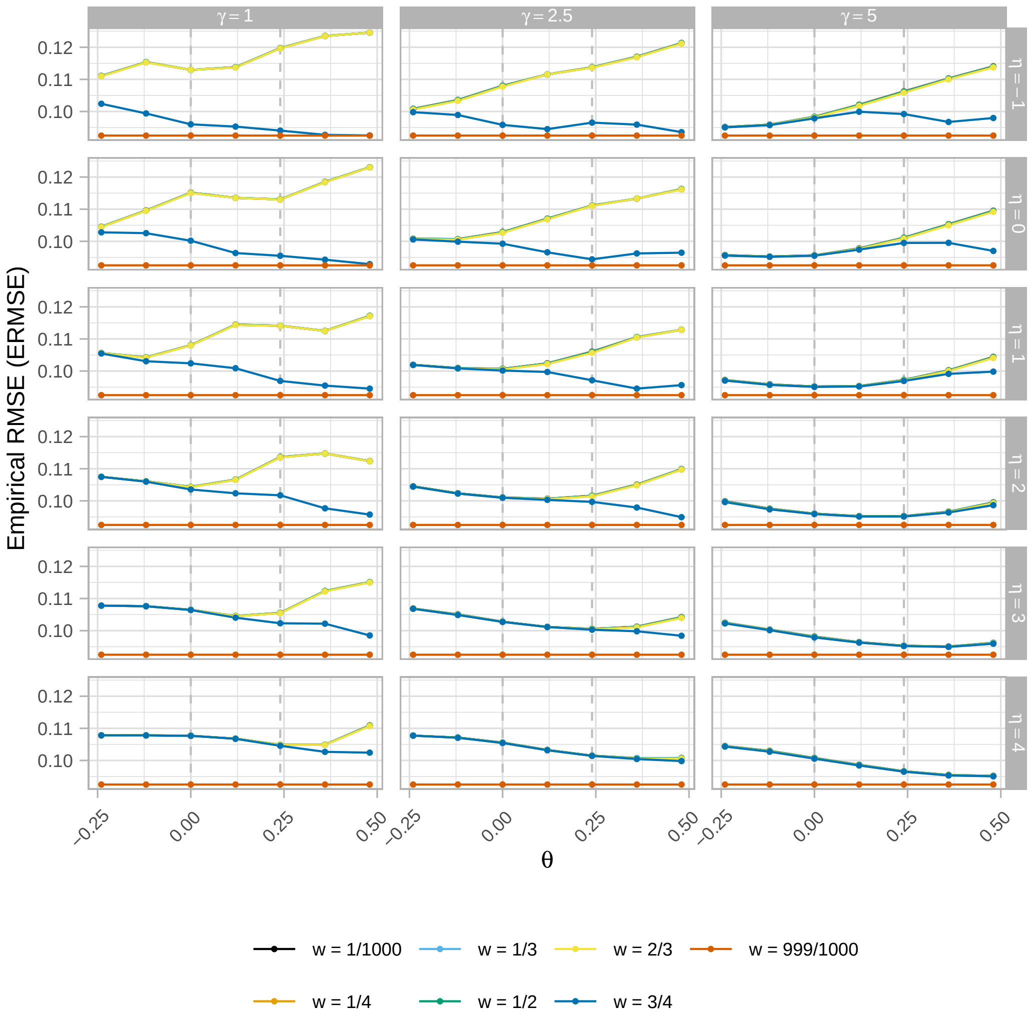

Figure S16: The empirical root-mean-square error (ERMSE), as a function of w and θ, of the response adaptive (RA) stepped-wedge cluster randomised trial (SW-CRT) designs with $\{p_1, \dots, p_L\} = \{4\}$ for different combinations of η and γ, in Trial Design Scenario 1 (TDS1).

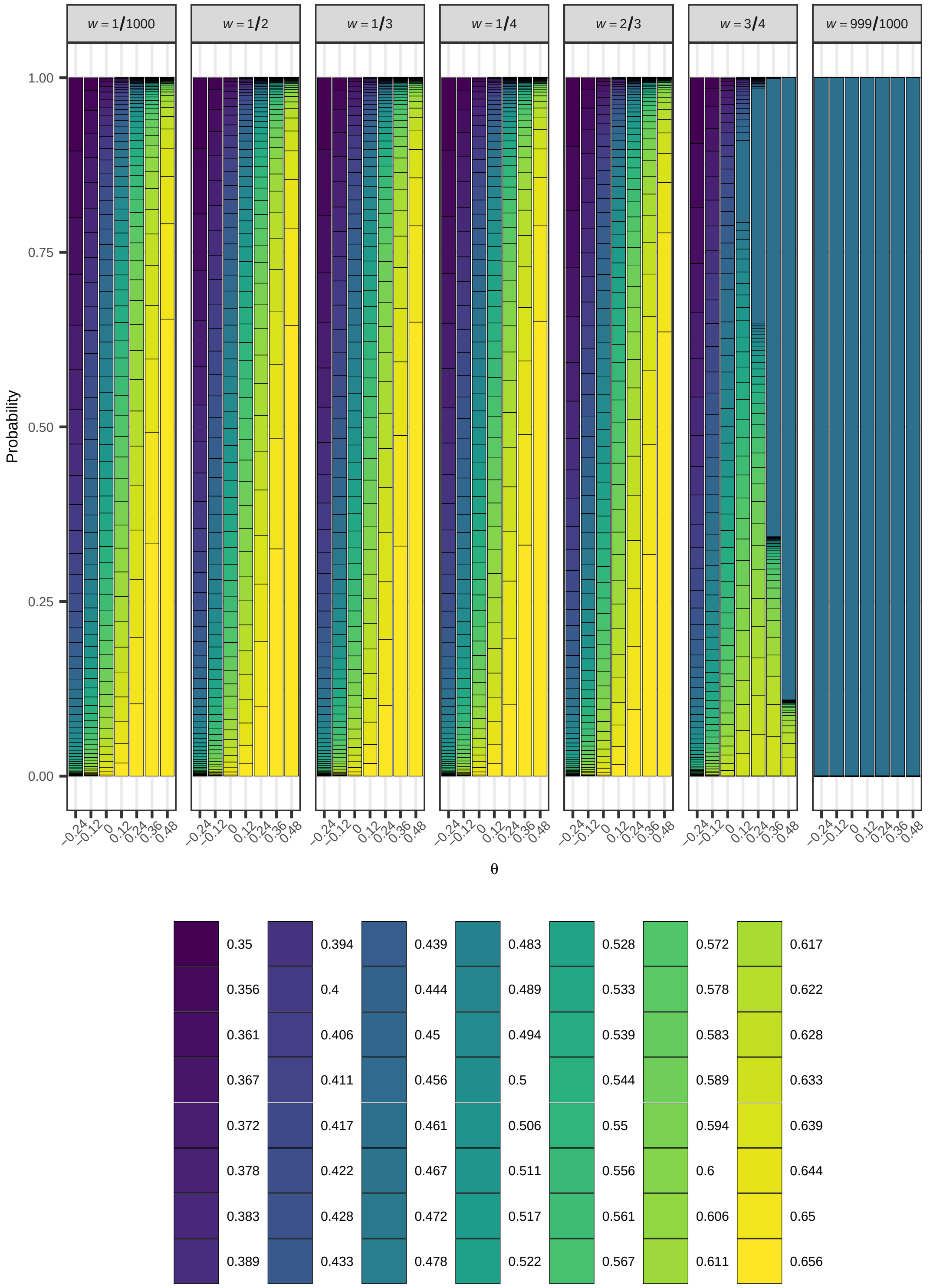

Figure S17: The empirical probability mass function of the proportion of cluster-periods spent in the intervention condition, as a function of θ and w, of the response adaptive (RA) stepped-wedge cluster randomised trial (SW-CRT) design with $\eta = 0$, $\gamma = 2.5$, and $\{p_1, \dots, p_L\} = \{4\}$, in Trial Design Scenario 1 (TDS1).

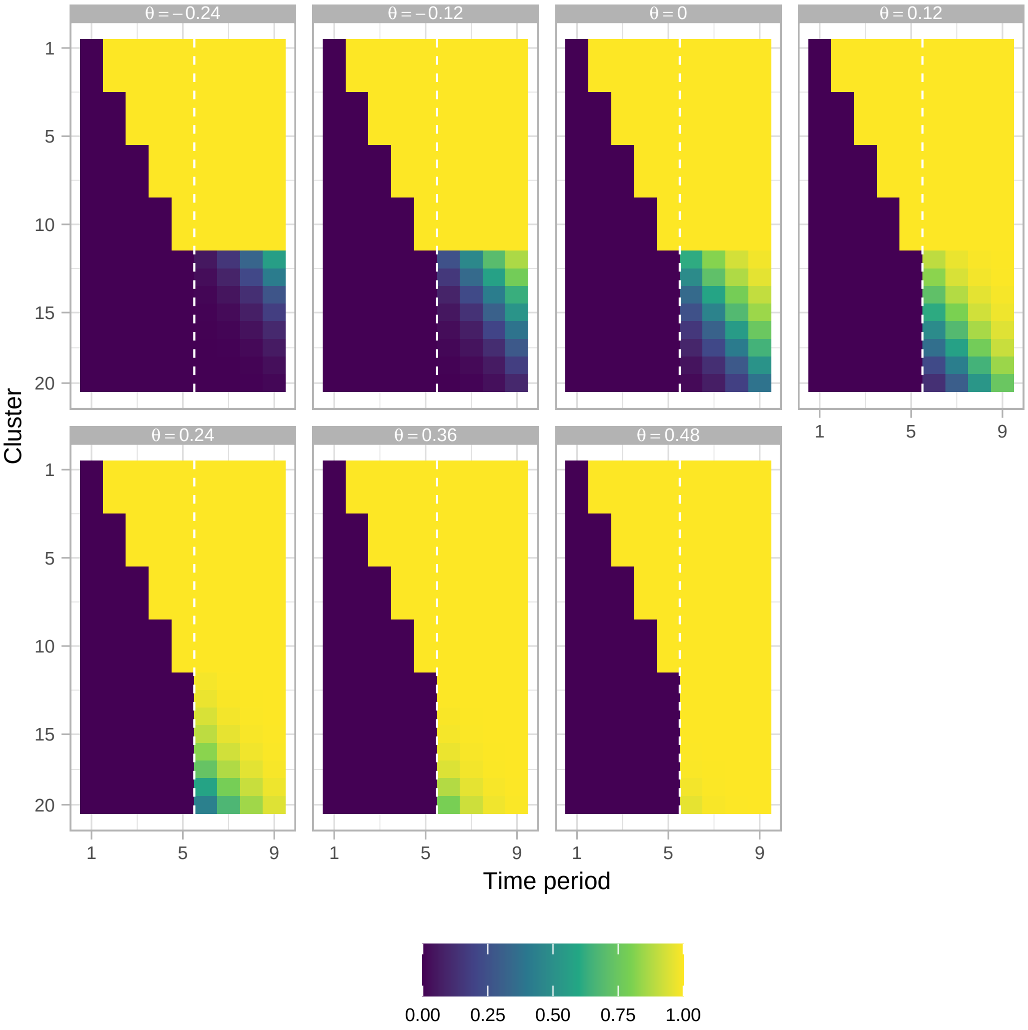

Figure S18: The empirical average final allocation matrix ($\bar{X}_P$), as a function of θ, of the response adaptive (RA) stepped-wedge cluster randomised trial (SW-CRT) design with $\eta = 0$, $\gamma = 2.5$, $w = 1/2$, and $\{p_1, \ldots, p_L\} = \{5\}$, in Trial Design Scenario 1 (TDS1). The dashed lines indicate the timing of the interim analyses.

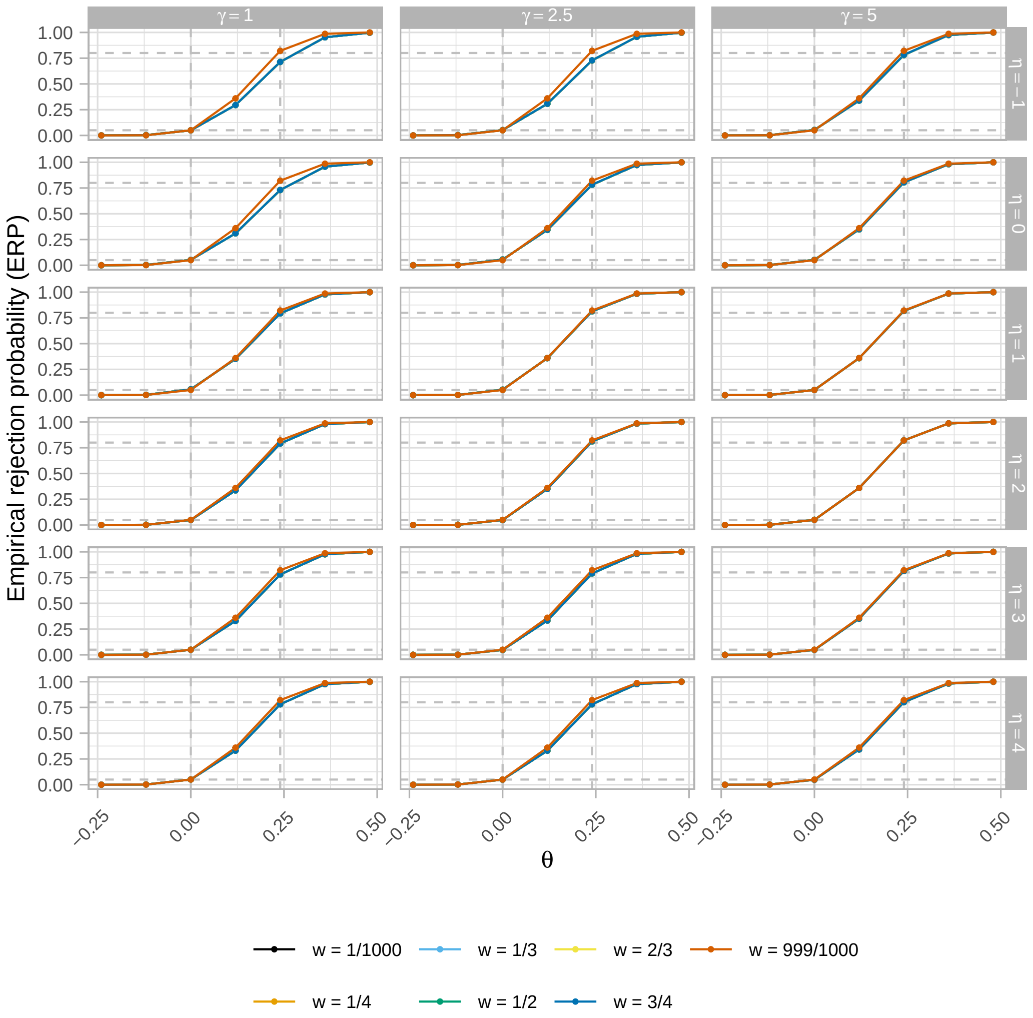

Figure S19: The empirical rejection probability (ERP), as a function of w and θ, of the response adaptive (RA) stepped-wedge cluster randomised trial (SW-CRT) designs with $\{p_1, \dots, p_L\} = \{5\}$ for different combinations of η and γ, in Trial Design Scenario 1 (TDS1). The dashed lines indicate the desired type-I and type-II error-rates.

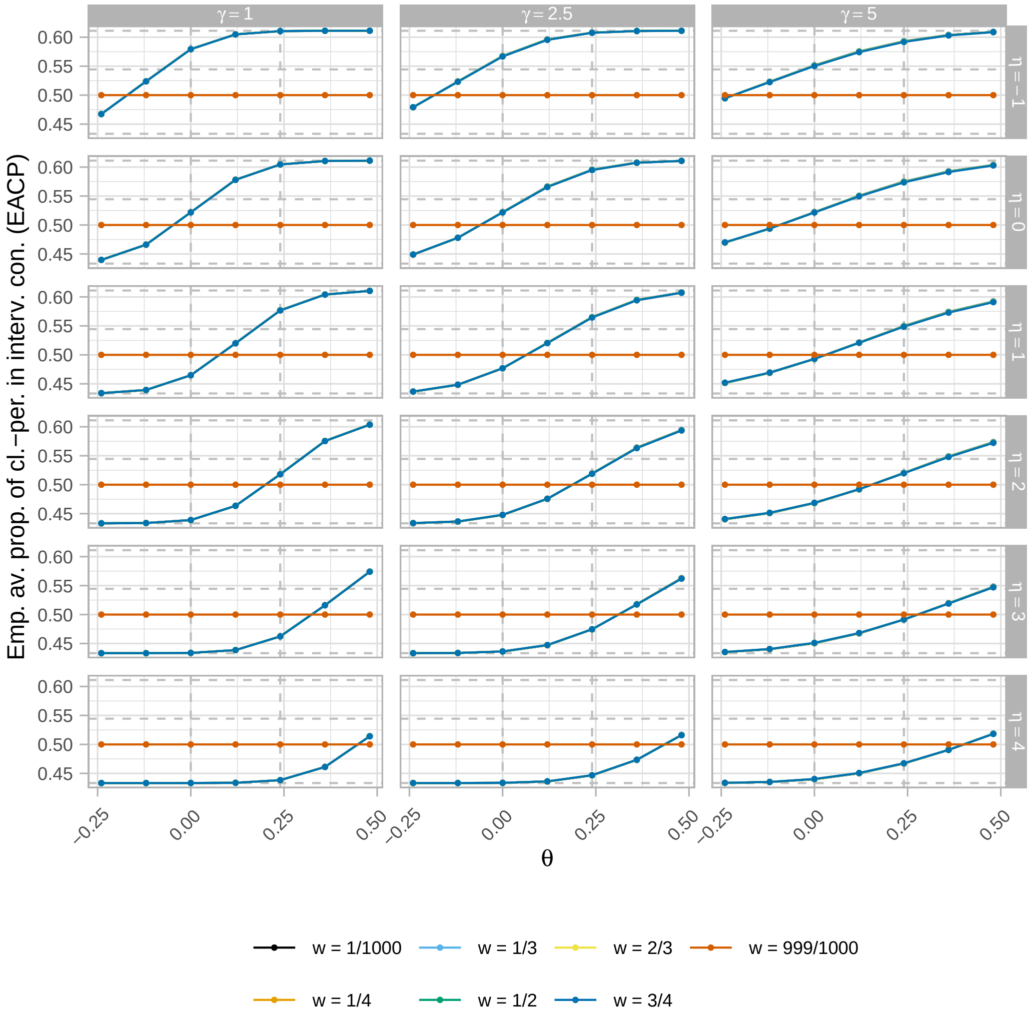

Figure S20: The empirical average proportion of cluster-periods spent in the intervention condition (EACP), as a function of w and θ, of the response adaptive (RA) stepped-wedge cluster randomised trial (SW-CRT) designs with $\{p_1, \dots, p_L\} = \{5\}$ for different combinations of η and γ, in Trial Design Scenario 1 (TDS1). The dashed lines indicate the minimal, initially planned, and maximal values of the EACP based on $X = X_{p_1}$.

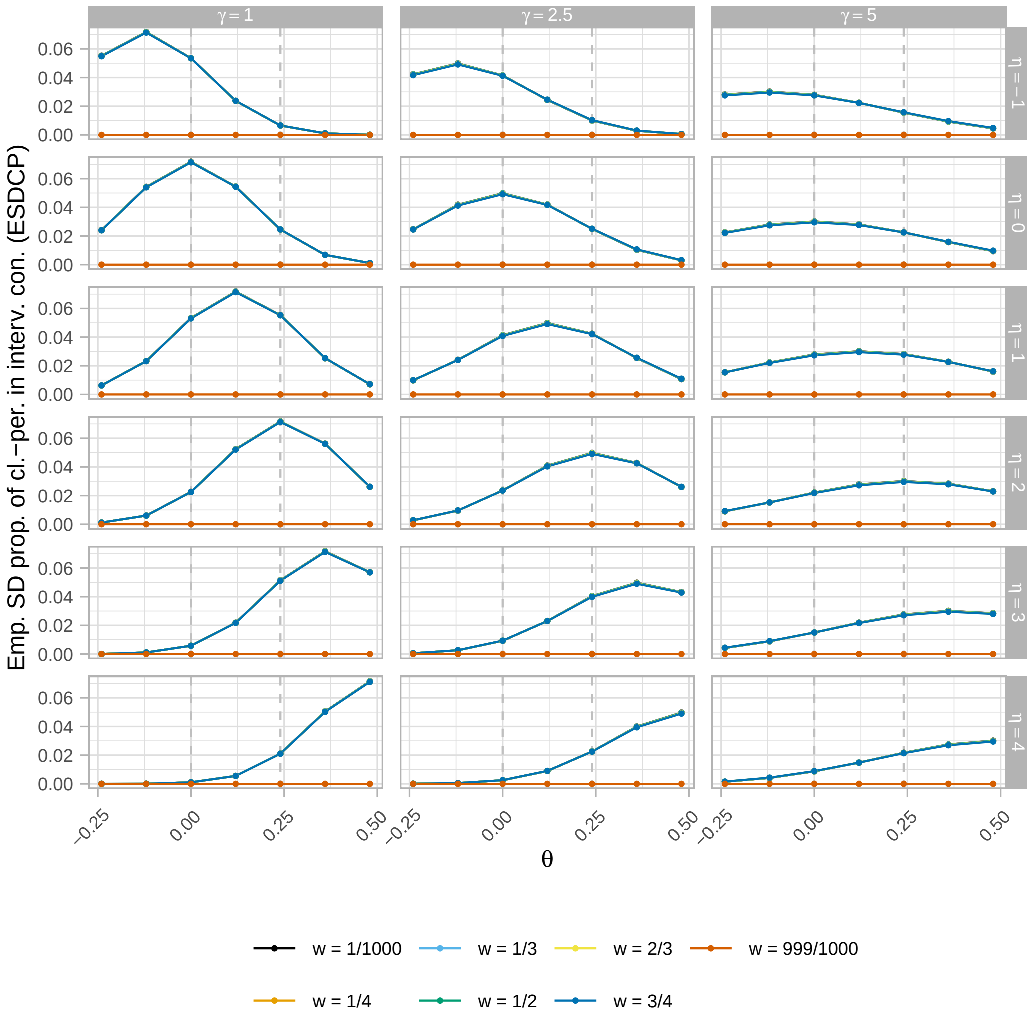

Figure S21: The empirical standard deviation of the proportion of cluster-periods spent in the intervention condition (ESDCP), as a function of w and θ, of the response adaptive (RA) stepped-wedge cluster randomised trial (SW-CRT) designs with $\{p_1, \ldots, p_L\} = \{5\}$ for different combinations of η and γ, in Trial Design Scenario 1 (TDS1).

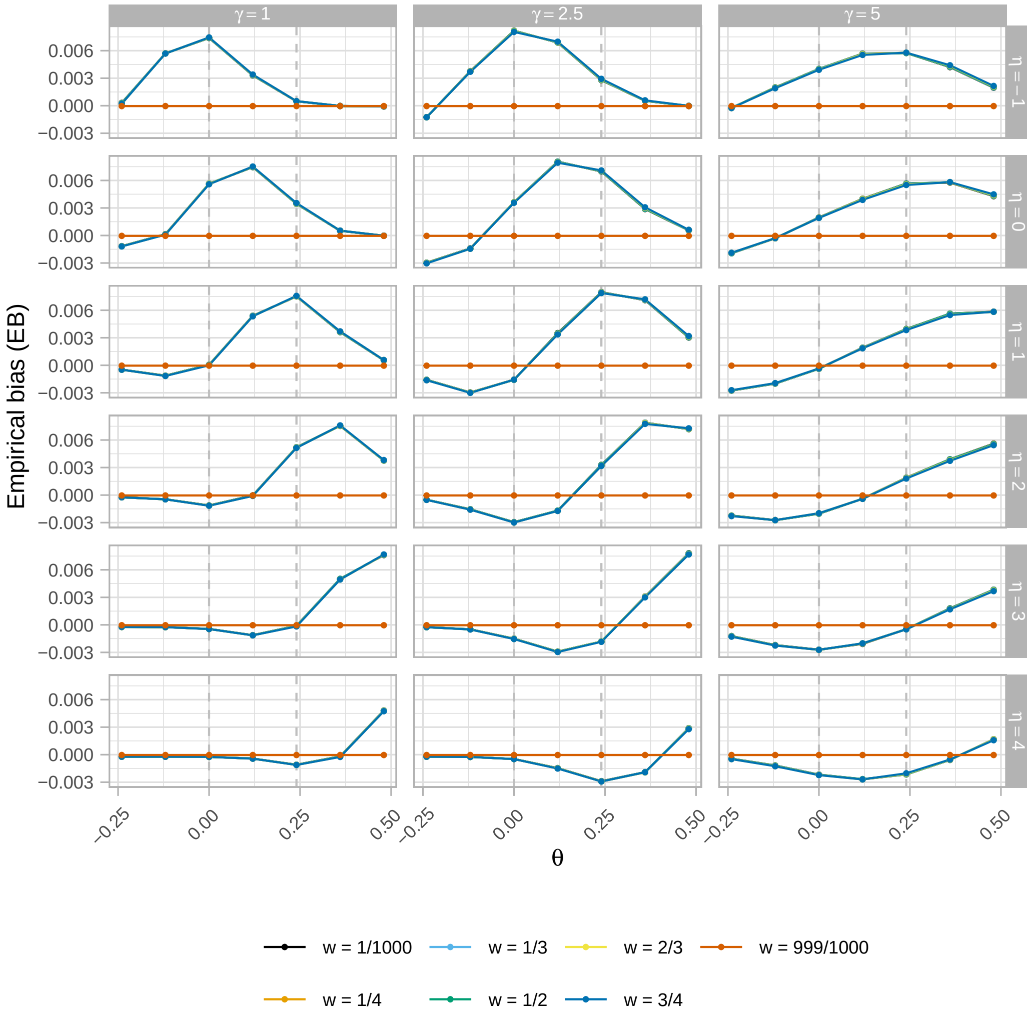

Figure S22: The empirical bias (EB), as a function of w and θ, of the response adaptive (RA) stepped-wedge cluster randomised trial (SW-CRT) designs with $\{p_1, \dots, p_L\} = \{5\}$ for different combinations of η and γ, in Trial Design Scenario 1 (TDS1).

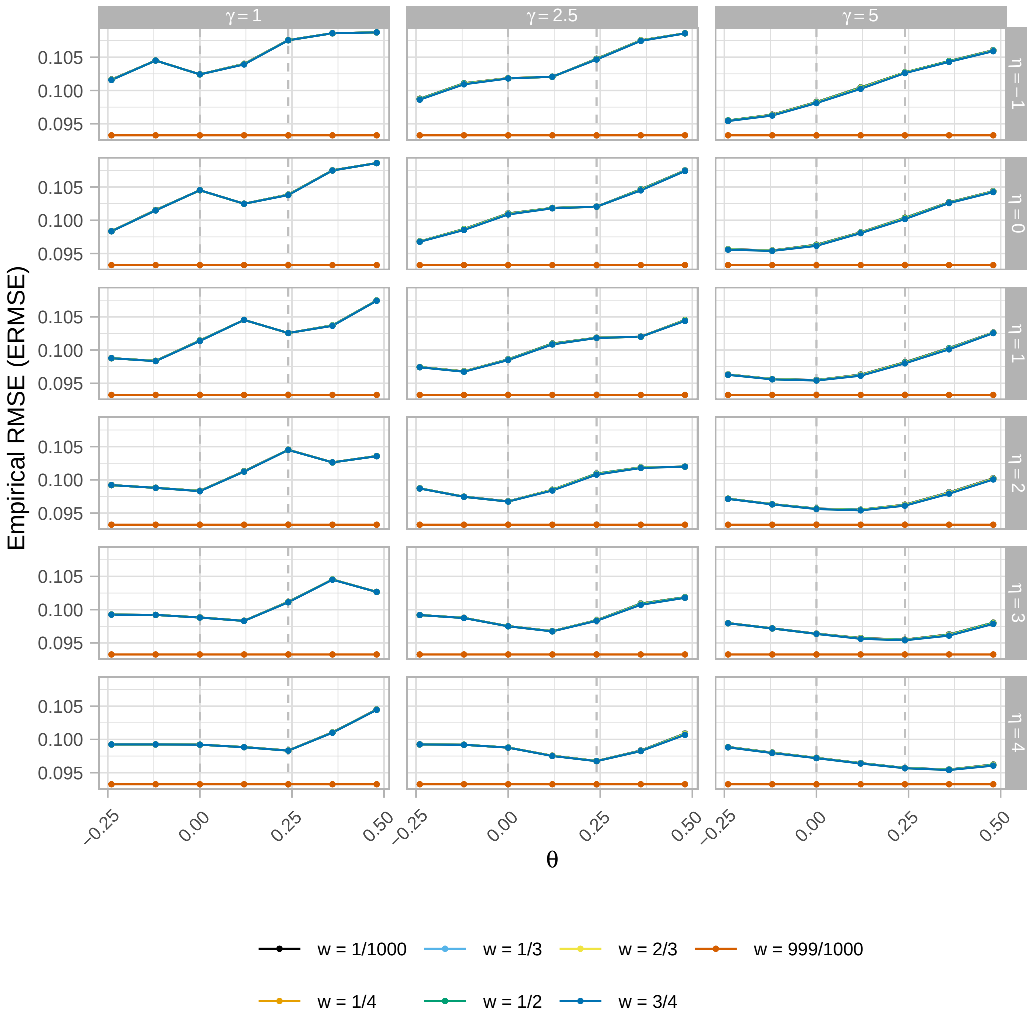

Figure S23: The empirical root-mean-square error (ERMSE), as a function of w and θ, of the response adaptive (RA) stepped-wedge cluster randomised trial (SW-CRT) designs with $\{p_1, \dots, p_L\} = \{5\}$ for different combinations of η and γ, in Trial Design Scenario 1 (TDS1).

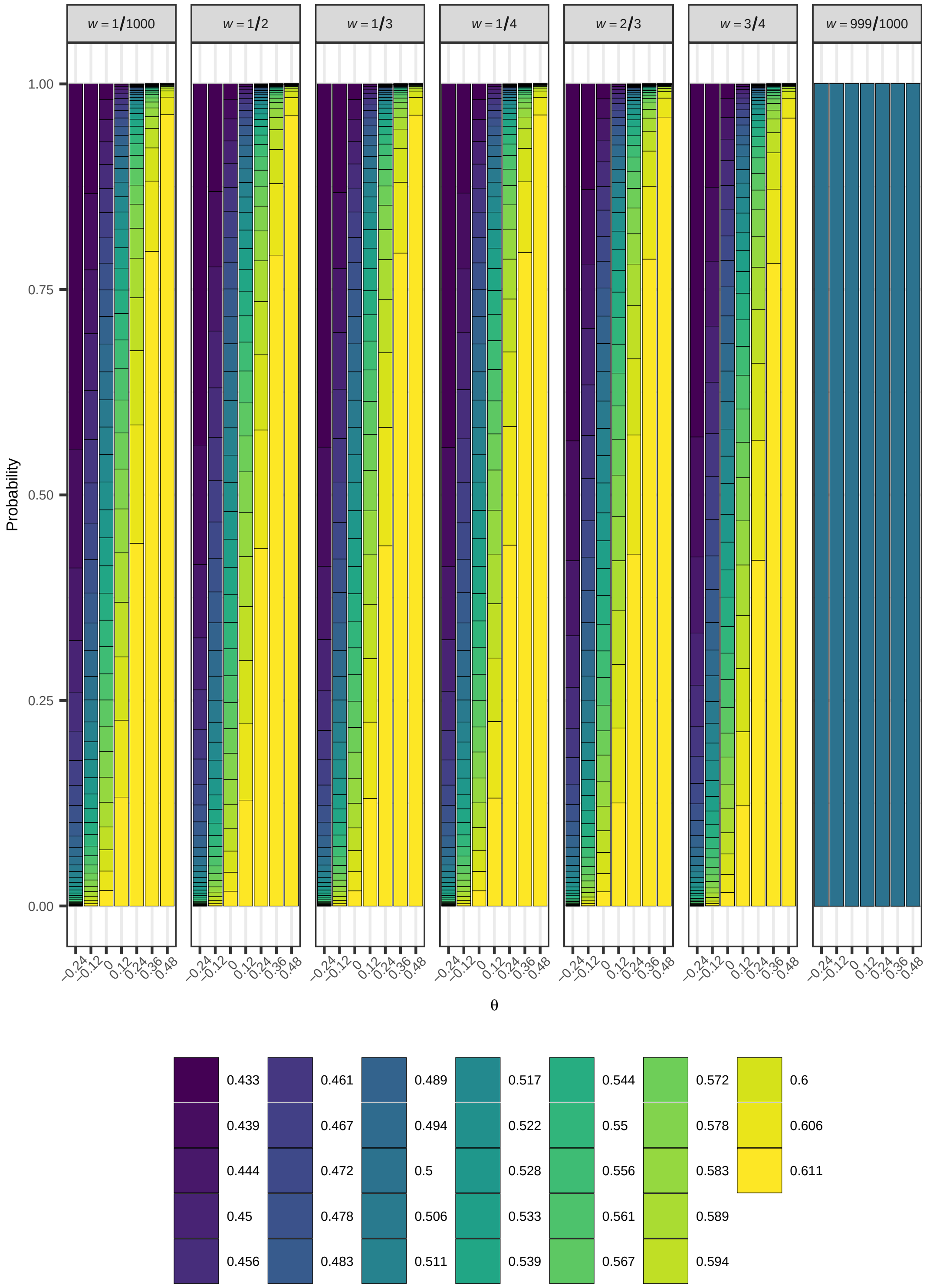

Figure S24: The empirical probability mass function of the proportion of cluster-periods spent in the intervention condition, as a function of θ and w, of the response adaptive (RA) stepped-wedge cluster randomised trial (SW-CRT) design with $\eta = 0$, $\gamma = 2.5$, and $\{p_1, \dots, p_L\} = \{5\}$, in Trial Design Scenario 1 (TDS1).

Trial Design Scenario 2

$\{p_1, \dots, p_L\} = \{3\}$

In this TDS, differentiation of the ERP and EACP is even less apparent with modification of w, η, and γ. The ERP curves in particular are little changed across all considered parameter combinations (Figure S26). This should not be surprising as placing the interim analysis after time period 3 means there are few possible modifications that can be made to the initially planned roll-out (Figures S25 and S31). Yet, importantly, there remains w such that the EACP is able to adaptively switch from low to high values as θ increases. For example, for $w = 1/2$, $\eta = 0$, and $\gamma = 2.5$, the EACP increases from 35.6% when $\theta = -\delta$ to 55.0% when $\theta = 2\delta$, attaining ERPs when $\theta = 0$ and $\theta = \delta$ of 5.6% and 86.2% respectively. The EB remains low across considered scenarios, while the ERMSE is arguably affected little by choosing $w \neq 999/1000$ relative to performance for $w = 999/1000$.

$\{p_1, \dots, p_L\} = \{2, 3, 4\}$

Increasing the number of interim analyses in this case has the effect of substantially widening the possible number of modified roll-outs. This is to the effect of a more pronounced differentiation of the ERP and EACP curves as a function of w, η, and γ (Figures S33-S34). Consequently, whilst there remains parameters combinations that attain approximately the desired error-rates whilst being able to increase the EACP in θ, fewer such parameter combinations exist.

Trial Design Scenario 3

$\{p_1, \dots, p_L\} = \{2\}$

Results in the considered closed-cohort setting echo these presented previously for cross-sectional designs. Clear transition of the EACP from small to larger values is apparent for numerous combinations of w, η, and γ. In this instance, it could be argued that a larger number of considered parameter combinations result in a notable power loss. However, there remains combinations for which the impact is small. For example, for $w = 1/2$, $\eta = 0$, and $\gamma = 2.5$, the EACP increases from 27.8% when $\theta = -\delta$ to 58.3% when $\theta = 2\delta$, attaining ERPs when $\theta = 0$ and $\theta = \delta$ of 3.2% and 85.6% respectively (Figures S41-S42).

$\{p_1, \dots, p_L\} = \{2, 3\}$

Increasing the number of interim analyses whilst retaining the same timing of the first interim analysis has limited effect, with the exception of the differentiation of performance across w for some η and γ parameter combinations (Figures S48-S52).

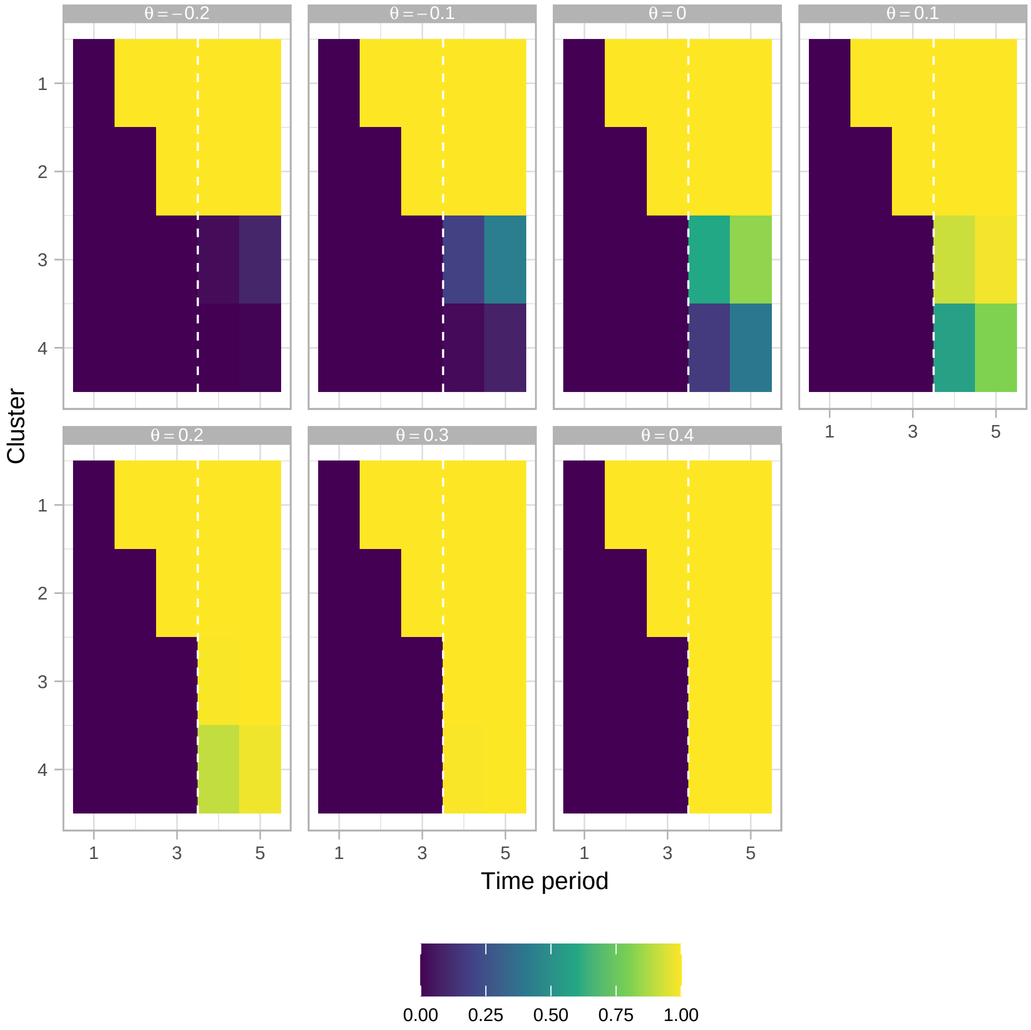

Figure S25: The empirical average final allocation matrix ($\bar{X}_P$), as a function of θ, of the response adaptive (RA) stepped-wedge cluster randomised trial (SW-CRT) design with $\eta = 0$, $\gamma = 2.5$, $w = 1/2$, and $\{p_1, \dots, p_L\} = \{3\}$, in Trial Design Scenario 2 (TDS2). The dashed lines indicate the timing of the interim analyses.

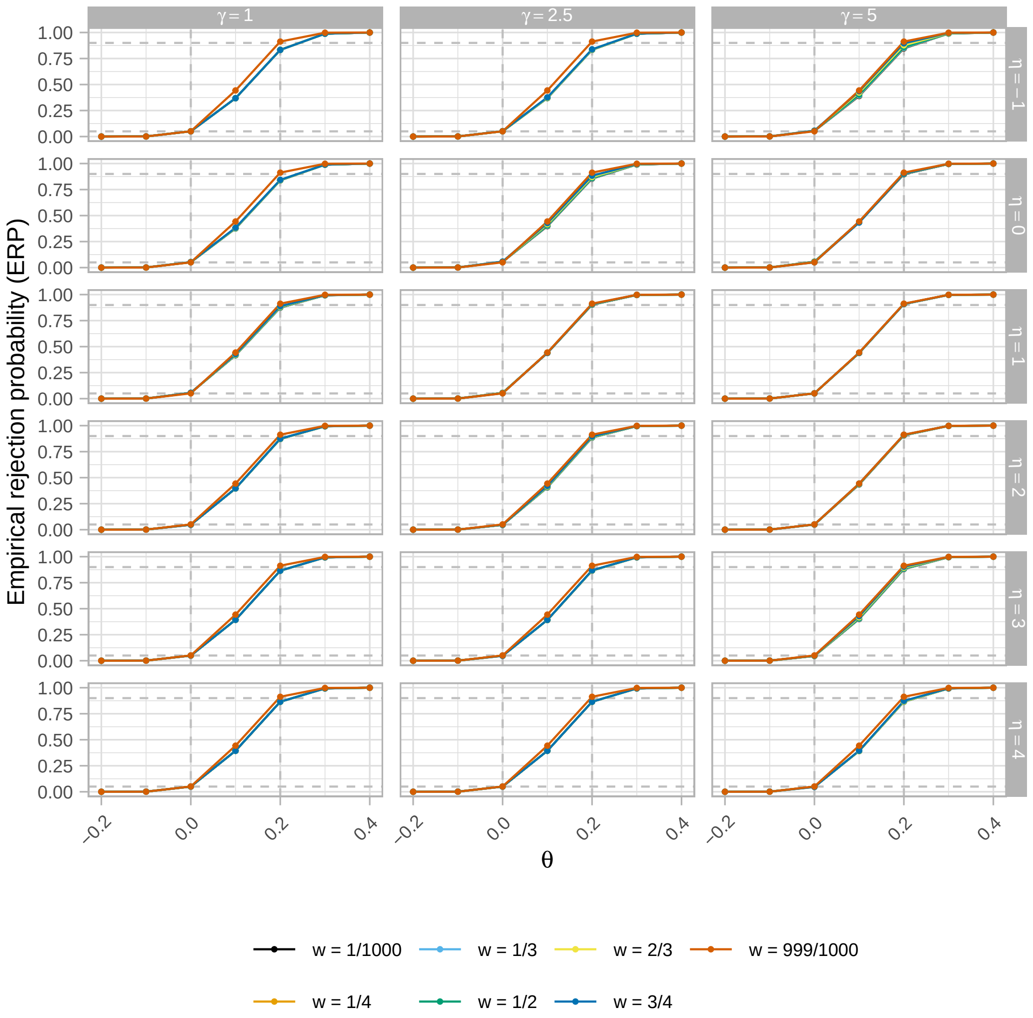

Figure S26: The empirical rejection probability (ERP), as a function of w and θ, of the response adaptive (RA) stepped-wedge cluster randomised trial (SW-CRT) designs with $\{p_1, \dots, p_L\} = \{3\}$ for different combinations of η and γ, in Trial Design Scenario 2 (TDS2). The dashed lines indicate the desired type-I and type-II error-rates.

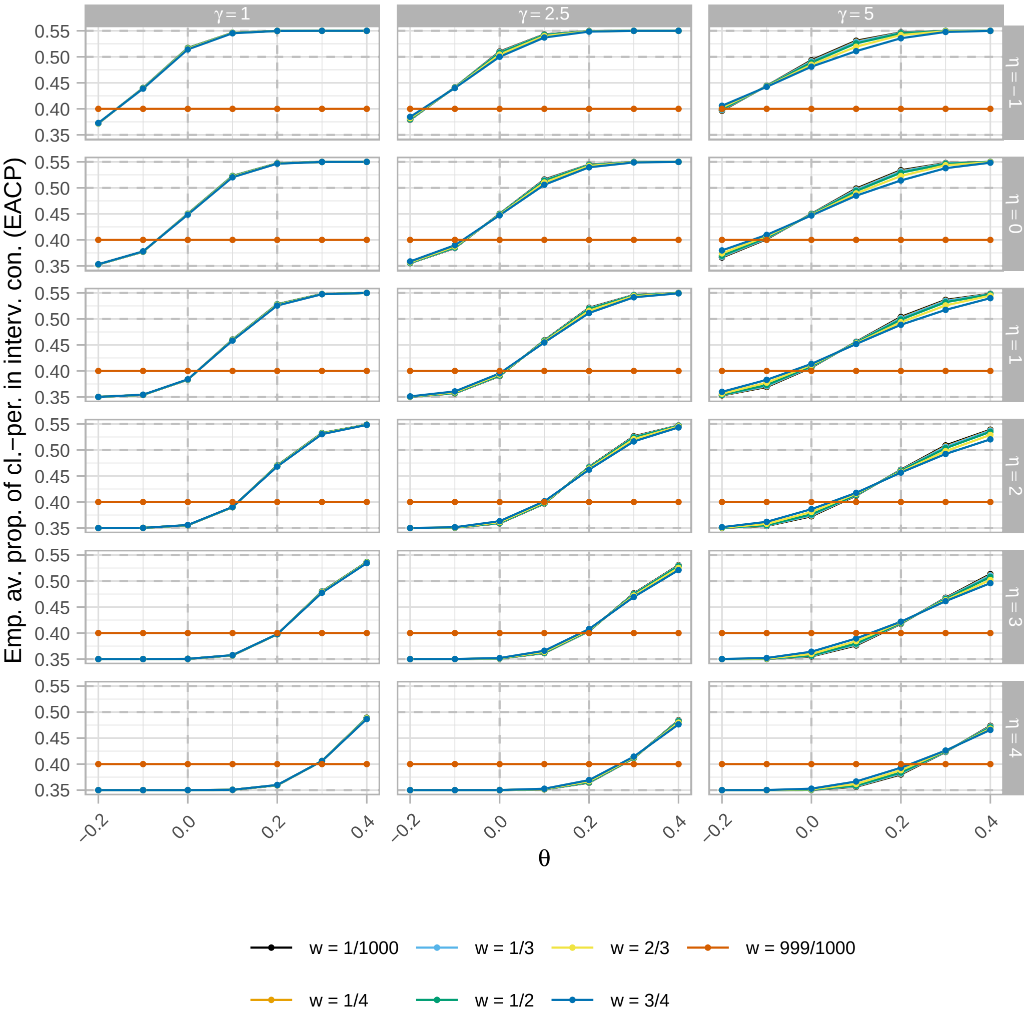

Figure S27: The empirical average proportion of cluster-periods spent in the intervention condition (EACP), as a function of w and θ, of the response adaptive (RA) stepped-wedge cluster randomised trial (SW-CRT) designs with $\{p_1, \ldots, p_L\} = \{3\}$ for different combinations of η and γ, in Trial Design Scenario 2 (TDS2). The dashed lines indicate the minimal, initially planned, and maximal values of the EACP based on $X = X_{p_1}$.

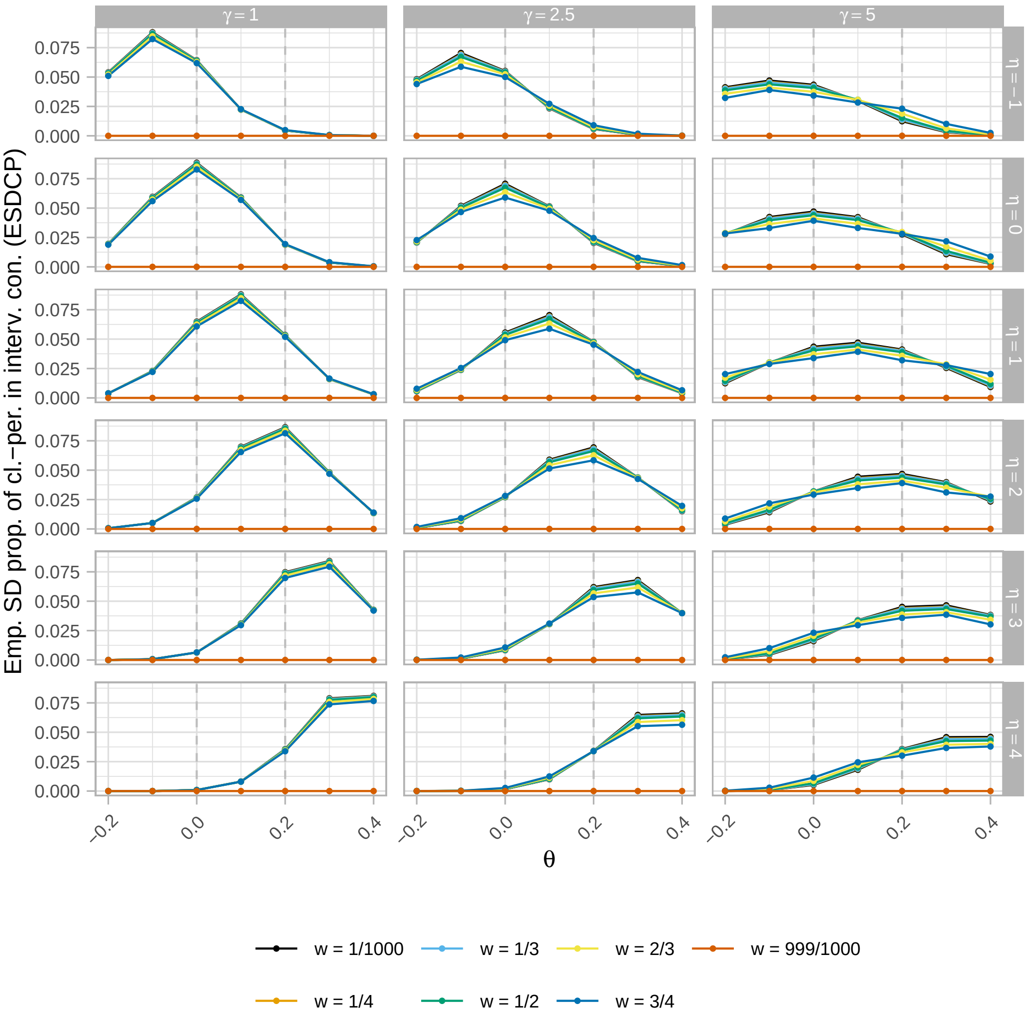

Figure S28: The empirical standard deviation of the proportion of cluster-periods spent in the intervention condition (ESDCP), as a function of w and θ, of the response adaptive (RA) stepped-wedge cluster randomised trial (SW-CRT) designs with $\{p_1, \dots, p_L\} = \{3\}$ for different combinations of η and γ, in Trial Design Scenario 2 (TDS2).

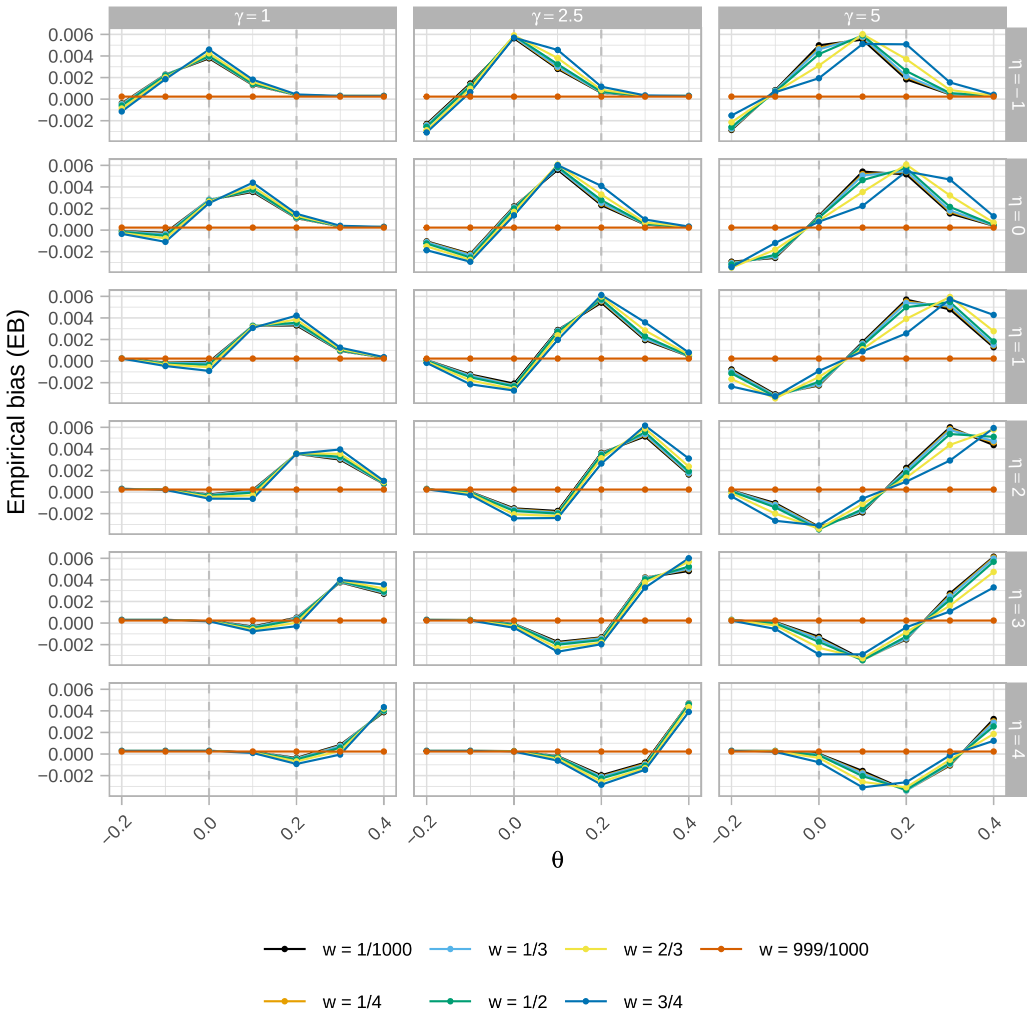

Figure S29: The empirical bias (EB), as a function of w and θ, of the response adaptive (RA) stepped-wedge cluster randomised trial (SW-CRT) designs with $\{p_1, \dots, p_L\} = \{3\}$ for different combinations of η and γ, in Trial Design Scenario 2 (TDS2).

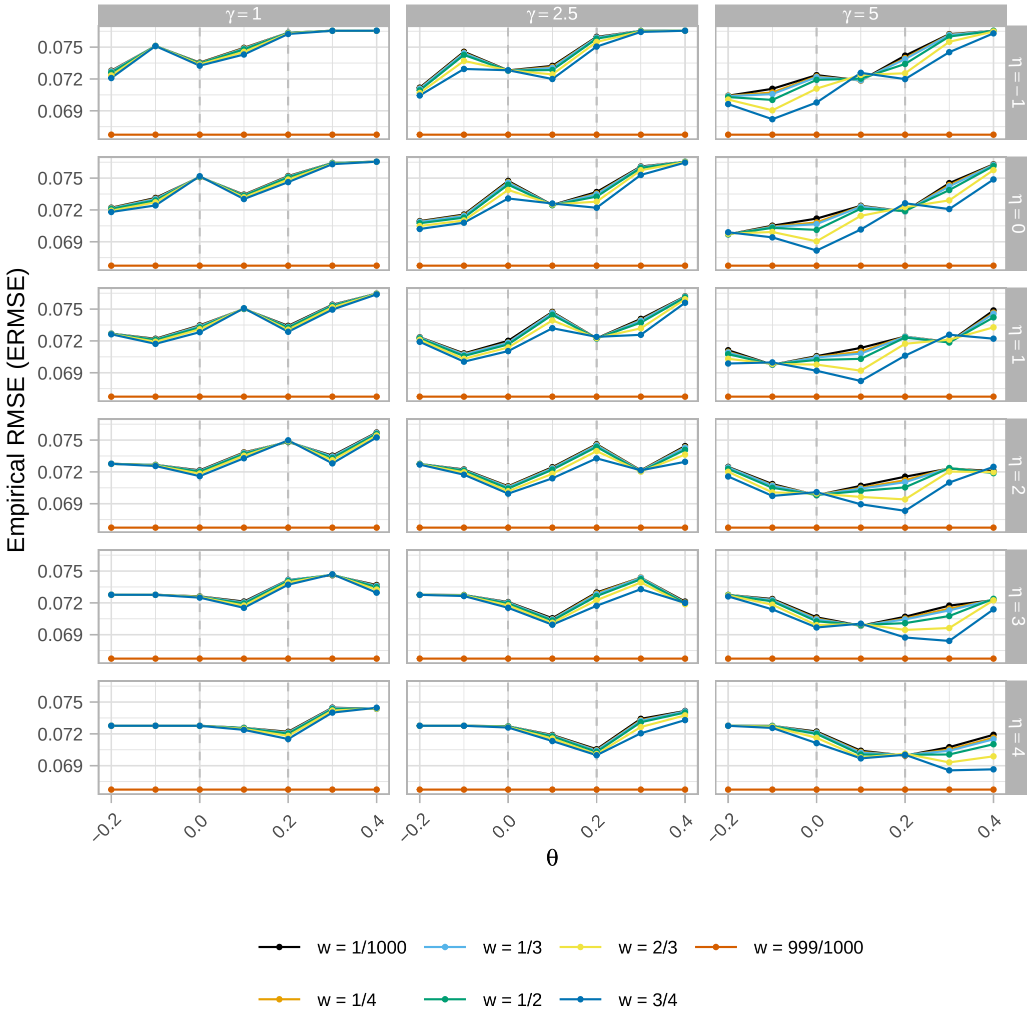

Figure S30: The empirical root-mean-square error (ERMSE), as a function of w and θ, of the response adaptive (RA) stepped-wedge cluster randomised trial (SW-CRT) designs with $\{p_1, \ldots, p_L\} = \{3\}$ for different combinations of η and γ, in Trial Design Scenario 2 (TDS2).

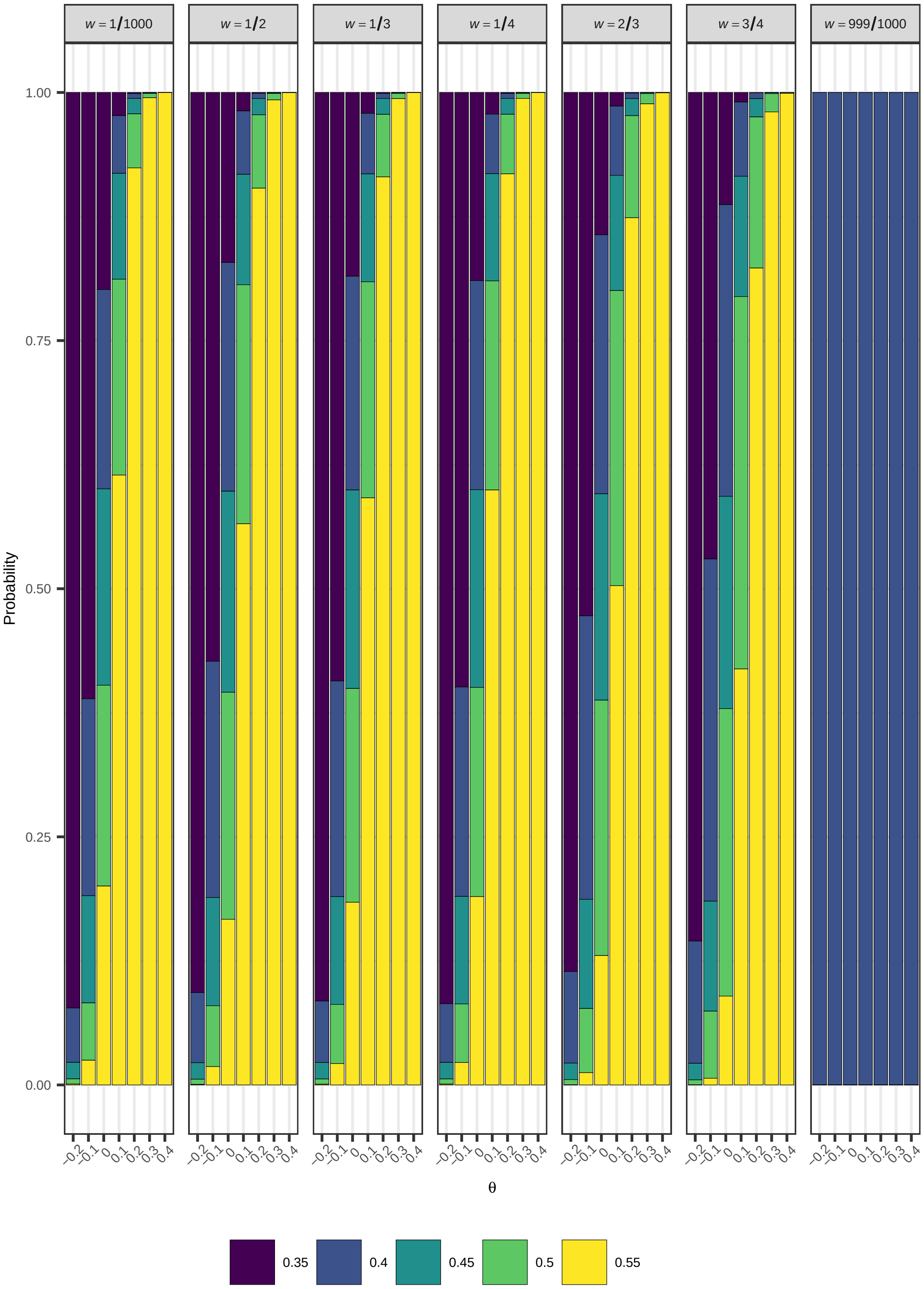

Figure S31: The empirical probability mass function of the proportion of cluster-periods spent in the intervention condition, as a function of θ and w, of the response adaptive (RA) stepped-wedge cluster randomised trial (SW-CRT) design with $\eta = 0$, $\gamma = 2.5$, and $\{p_1, \dots, p_L\} = \{3\}$, in Trial Design Scenario 2 (TDS2).

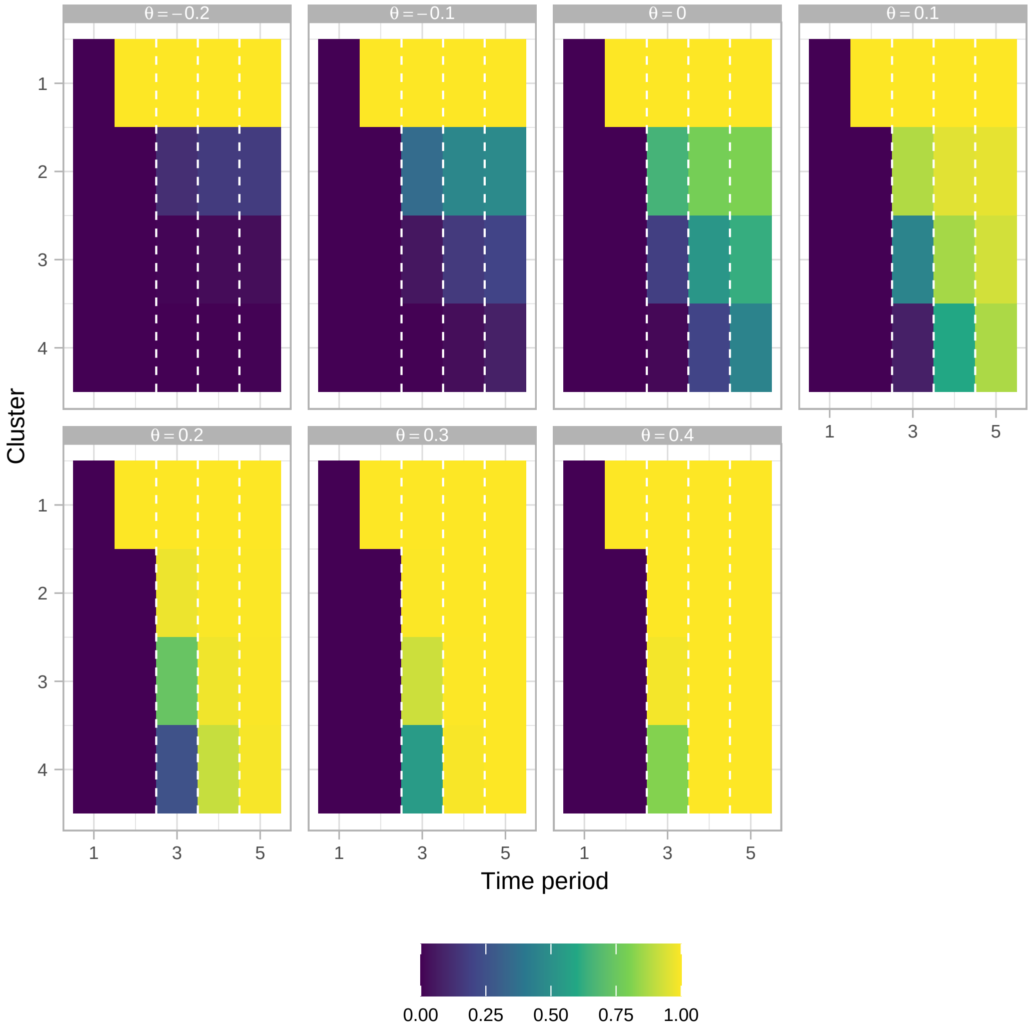

Figure S32: The empirical average final allocation matrix ($\bar{X}_P$), as a function of θ, of the response adaptive (RA) stepped-wedge cluster randomised trial (SW-CRT) design with $\eta = 0$, $\gamma = 2.5$, $w = 1/2$, and $\{p_1, \dots, p_L\} = \{2, 3, 4\}$, in Trial Design Scenario 2 (TDS2). The dashed lines indicate the timing of the interim analyses.

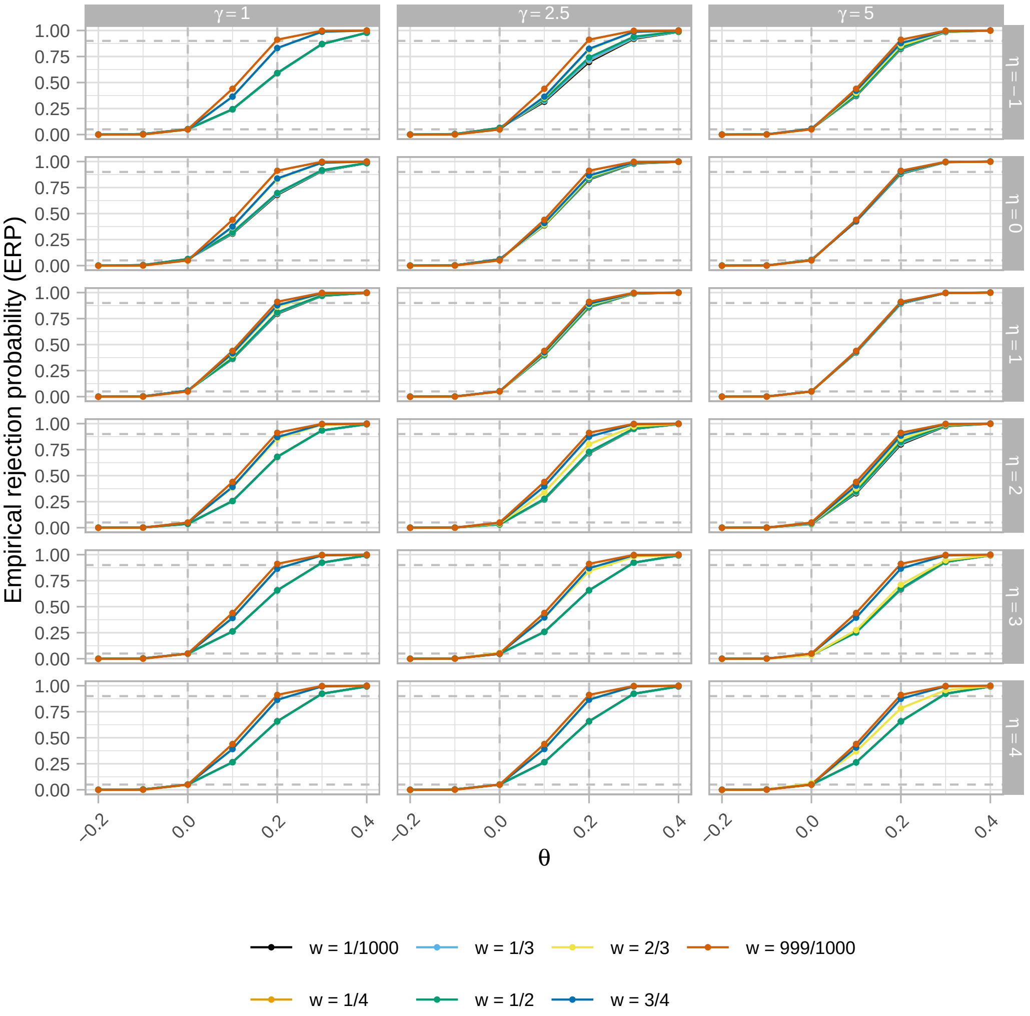

Figure S33: The empirical rejection probability (ERP), as a function of w and θ, of the response adaptive (RA) stepped-wedge cluster randomised trial (SW-CRT) designs with $\{p_1, \dots, p_L\} = \{2, 3, 4\}$ for different combinations of η and γ, in Trial Design Scenario 2 (TDS2). The dashed lines indicate the desired type-I and type-II error-rates.

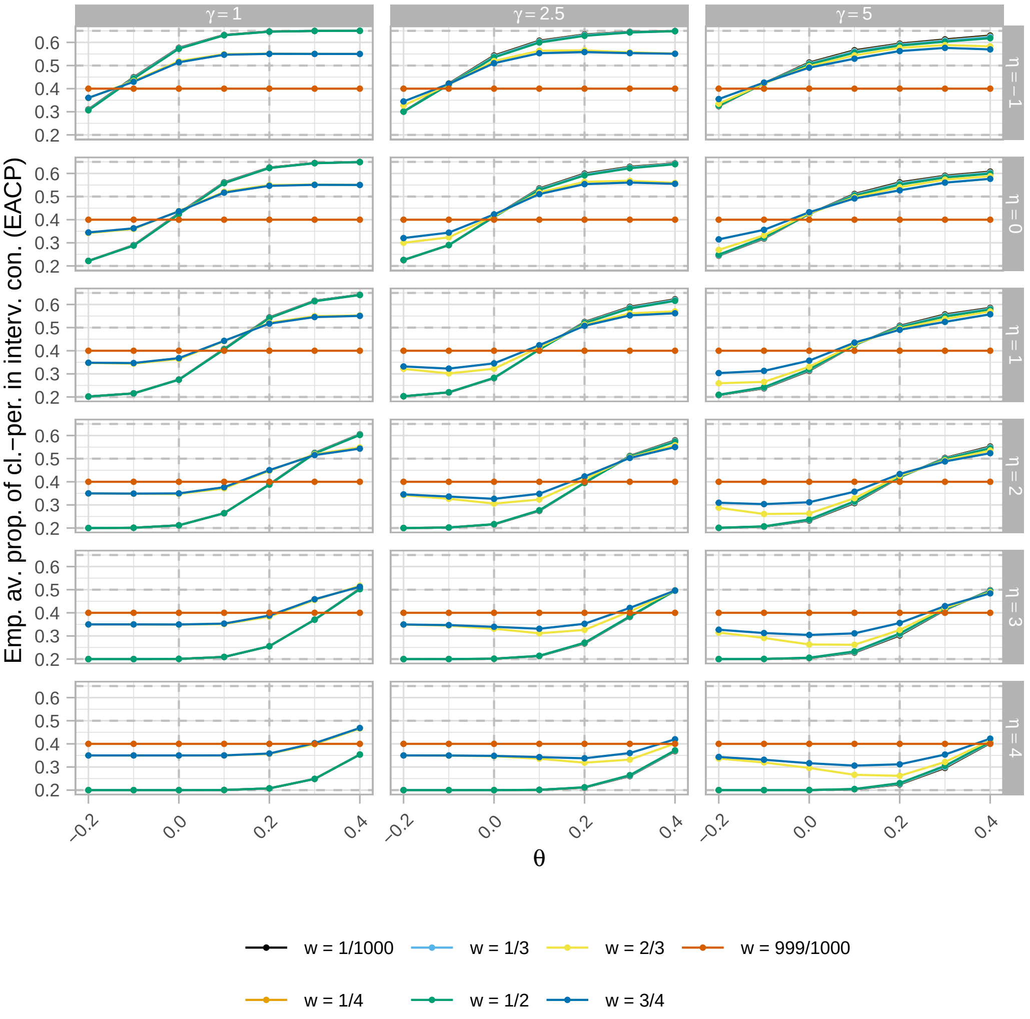

Figure S34: The empirical average proportion of cluster-periods spent in the intervention condition (EACP), as a function of w and θ, of the response adaptive (RA) stepped-wedge cluster randomised trial (SW-CRT) designs with $\{p_1, \ldots, p_L\} = \{3\}$ for different combinations of η and γ, in Trial Design Scenario 2 (TDS2). The dashed lines indicate the minimal, initially planned, and maximal values of the EACP based on $X = X_{p_1}$.

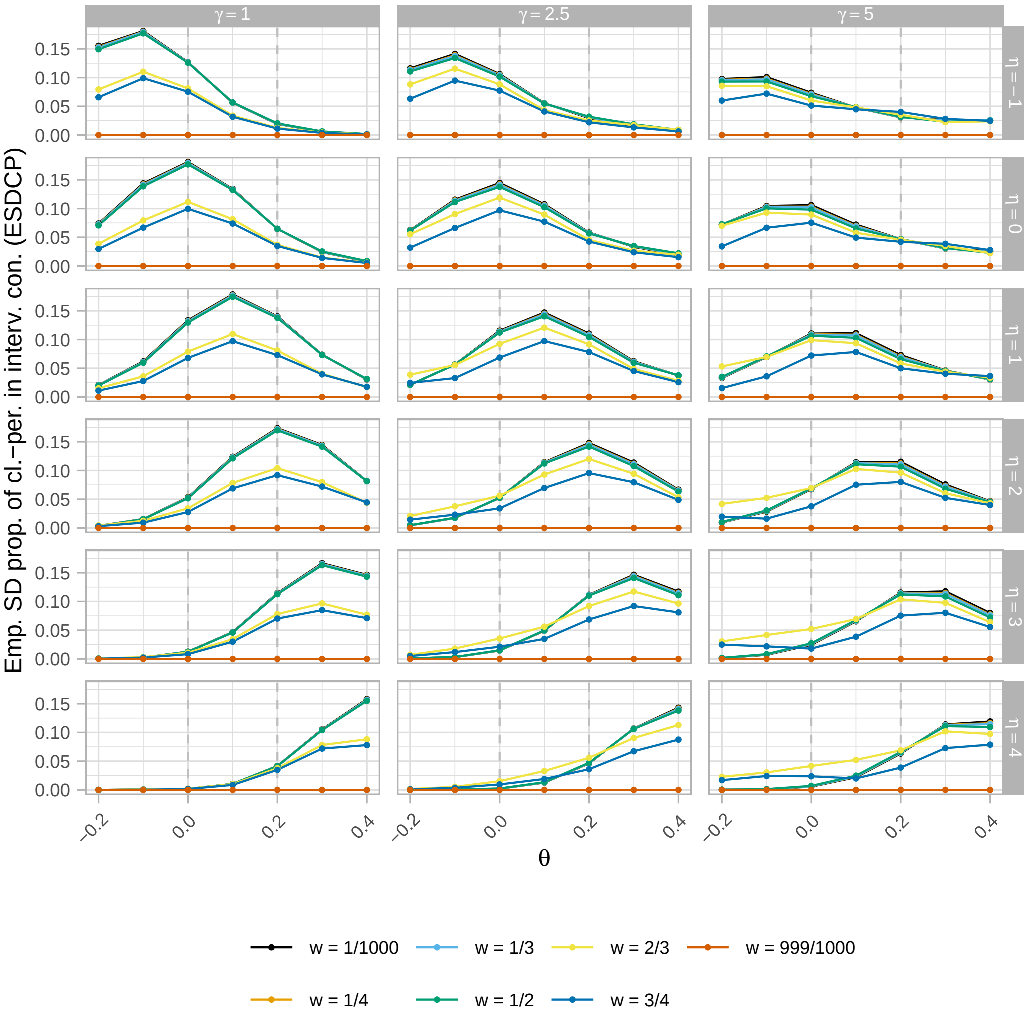

Figure S35: The empirical standard deviation of the proportion of cluster-periods spent in the intervention condition (ESDCP), as a function of w and θ, of the response adaptive (RA) stepped-wedge cluster randomised trial (SW-CRT) designs with $\{p_1, \ldots, p_L\} = \{2, 3, 4\}$ for different combinations of η and γ, in Trial Design Scenario 2 (TDS2).

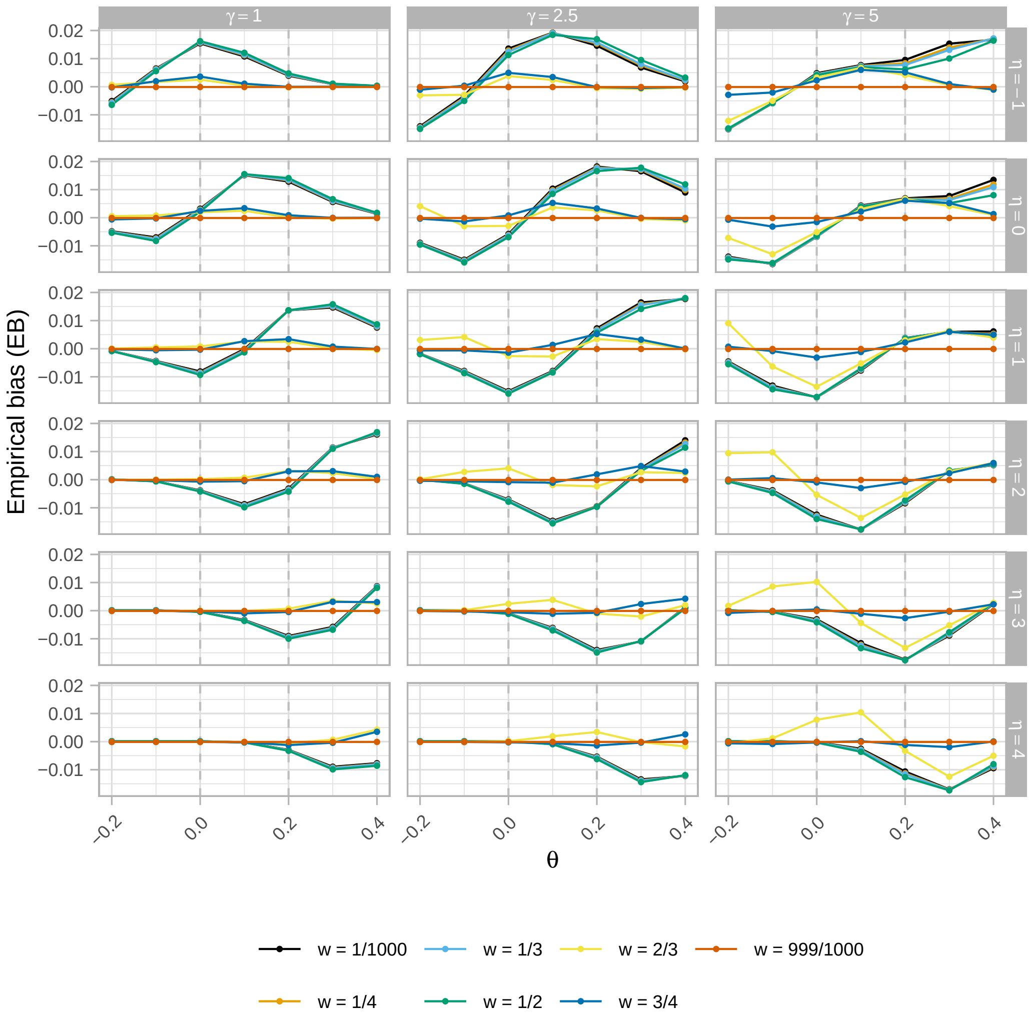

Figure S36: The empirical bias (EB), as a function of w and θ, of the response adaptive (RA) stepped-wedge cluster randomised trial (SW-CRT) designs with $\{p_1, \dots, p_L\} = \{2, 3, 4\}$ for different combinations of η and γ, in Trial Design Scenario 2 (TDS2).

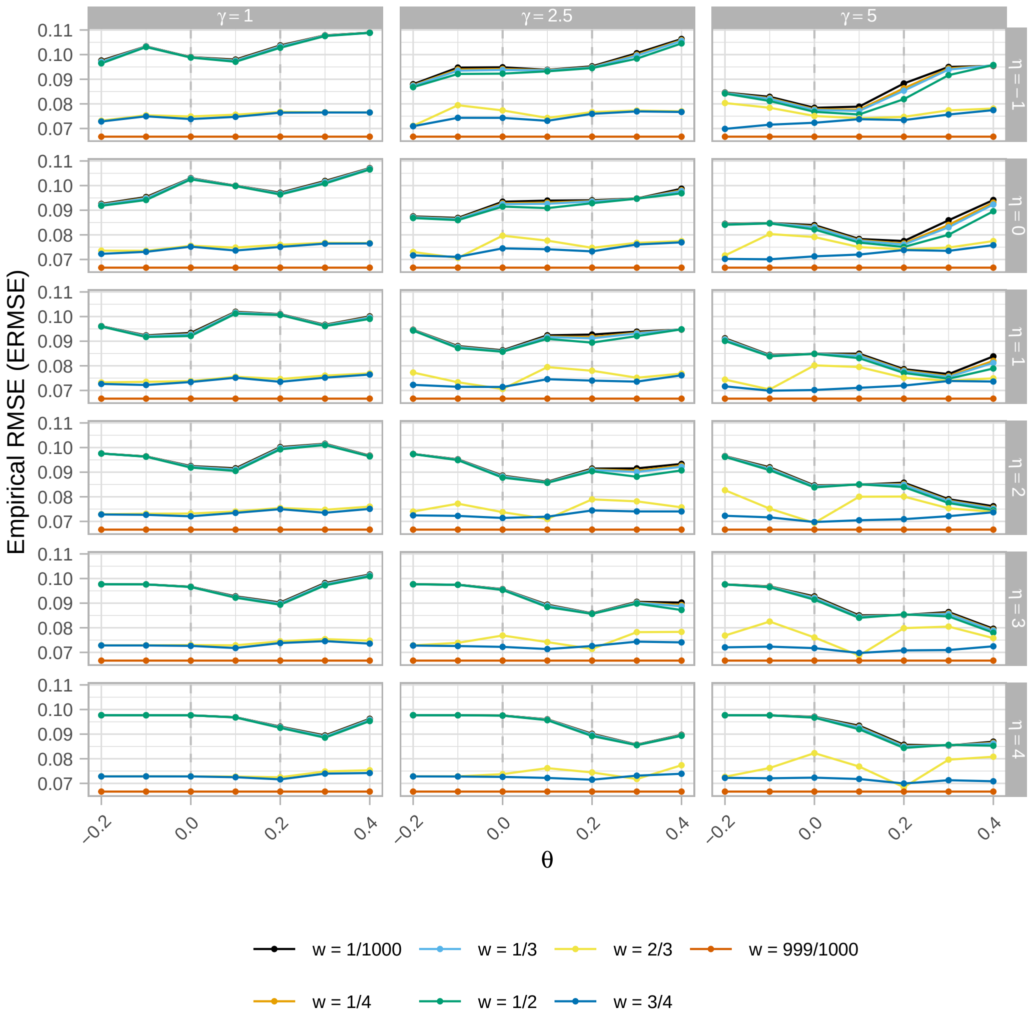

Figure S37: The empirical root-mean-square error (ERMSE), as a function of w and θ, of the response adaptive (RA) stepped-wedge cluster randomised trial (SW-CRT) designs with $\{p_1, \dots, p_L\} = \{2, 3, 4\}$ for different combinations of η and γ, in Trial Design Scenario 2 (TDS2).

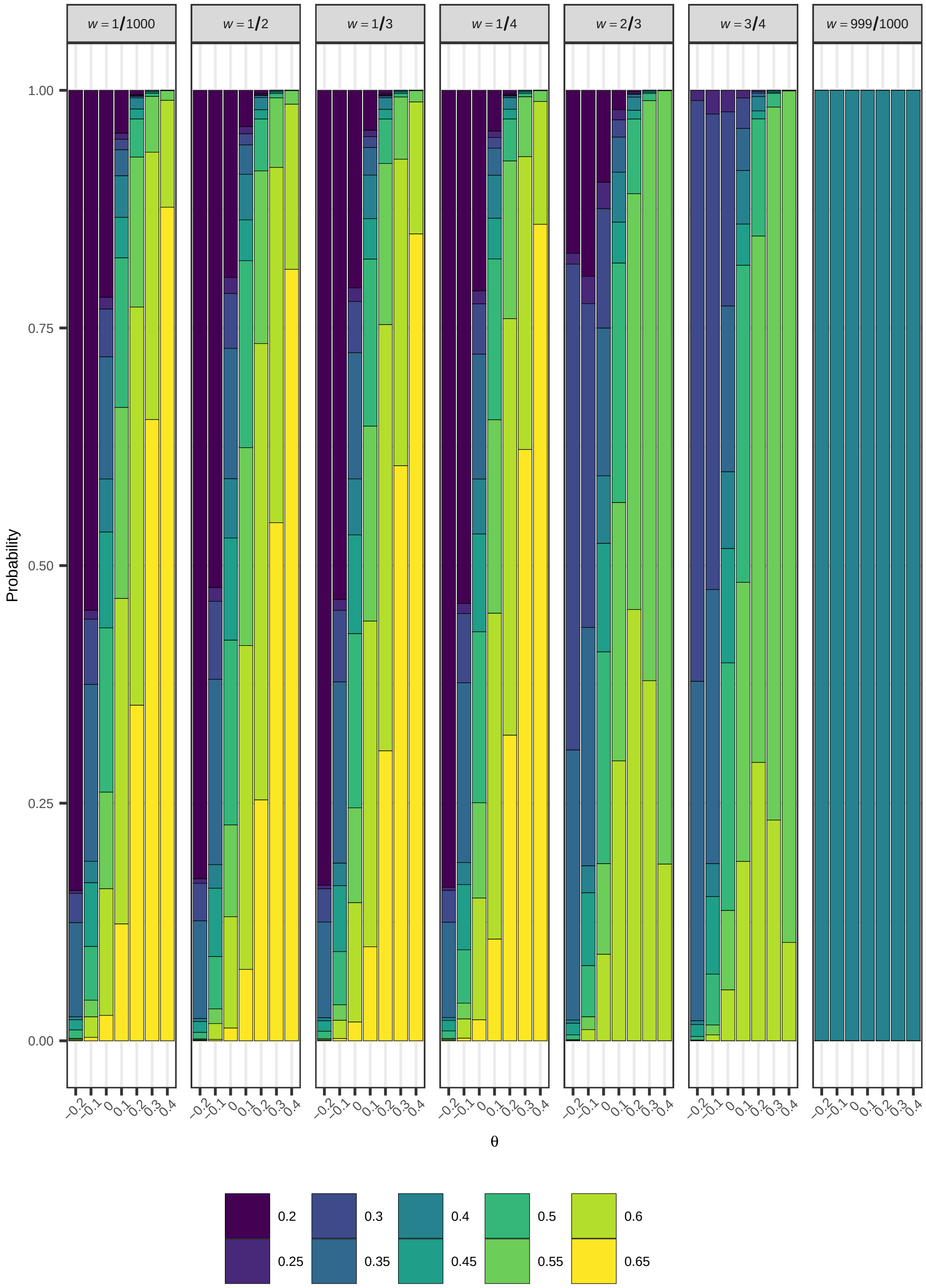

Figure S38: The empirical probability mass function of the proportion of cluster-periods spent in the intervention condition, as a function of θ and w, of the response adaptive (RA) stepped-wedge cluster randomised trial (SW-CRT) design with $\eta = 0$, $\gamma = 2.5$, and $\{p_1, \dots, p_L\} = \{2, 3, 4\}$, in Trial Design Scenario 2 (TDS2).

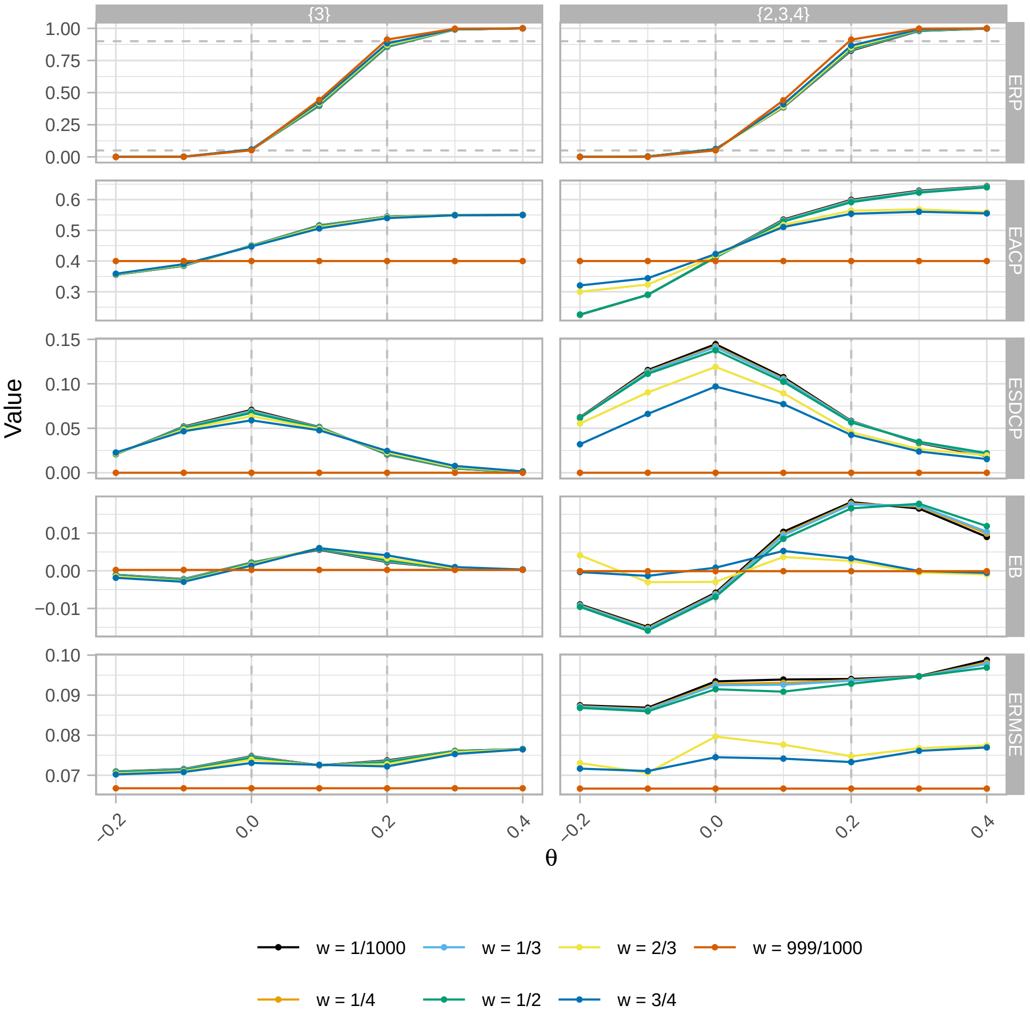

Figure S39: The empirical rejection probability (ERP), average proportion of cluster-periods spent in the intervention condition (EACP), standard deviation of cluster-periods spent in the intervention condition (EACP), bias (EB), and root-mean-square error (RMSE), as functions of w and θ, of the response adaptive (RA) stepped-wedge cluster randomised trial (SW-CRT) designs with $\eta = 0$ and $\gamma = 2.5$, for different values of $\{p_1, \dots, p_L\}$, in Trial Design Scenario 2 (TDS2). The dashed lines in the ERP plot indicate the desired type-I and type-II error-rates. In the EACP plot they indicate the minimal, initially planned, and maximal values of the EACP based on $X = X_{p_1}$.

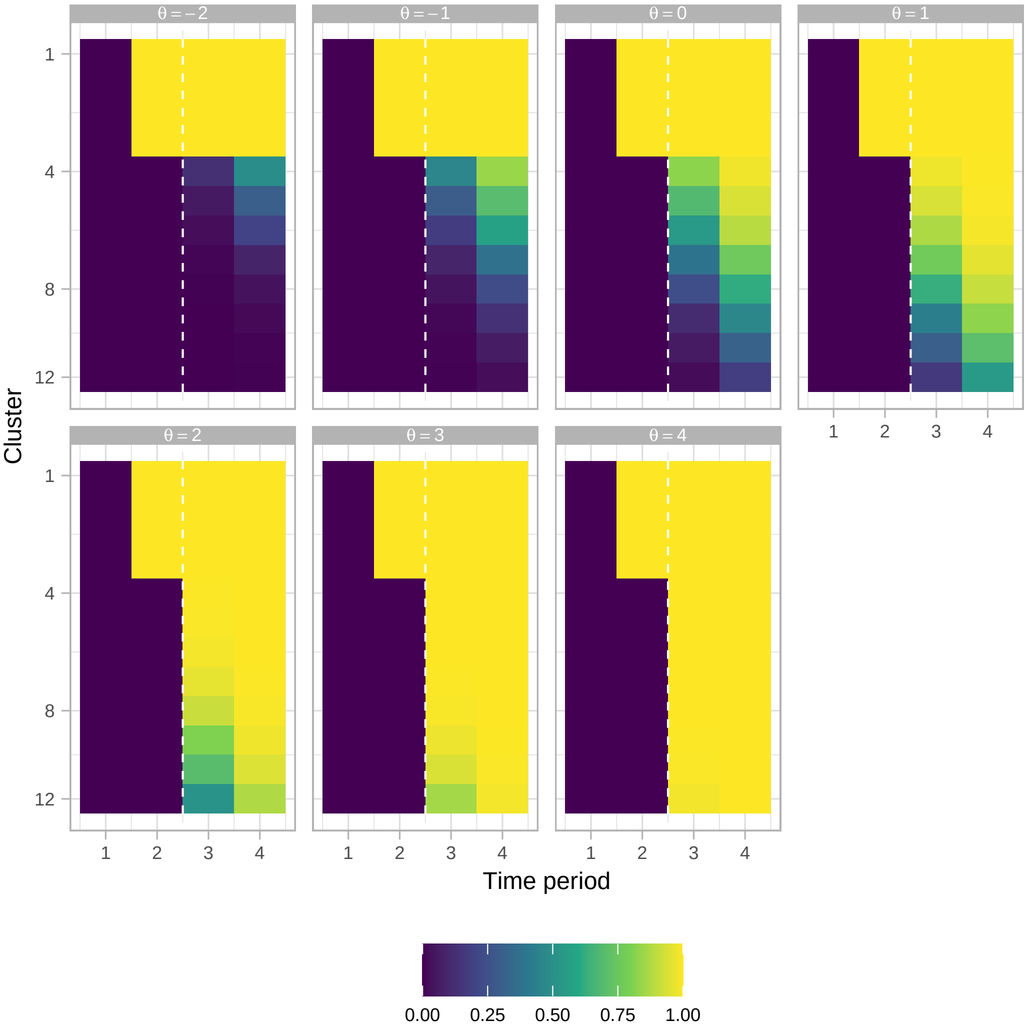

Figure S40: The empirical average final allocation matrix ($\bar{X}_P$), as a function of θ, of the response adaptive (RA) stepped-wedge cluster randomised trial (SW-CRT) design with $\eta = 0$, $\gamma = 2.5$, $w = 1/2$, and $\{p_1, \dots, p_L\} = \{2\}$, in Trial Design Scenario 3 (TDS3). The dashed lines indicate the timing of the interim analyses.

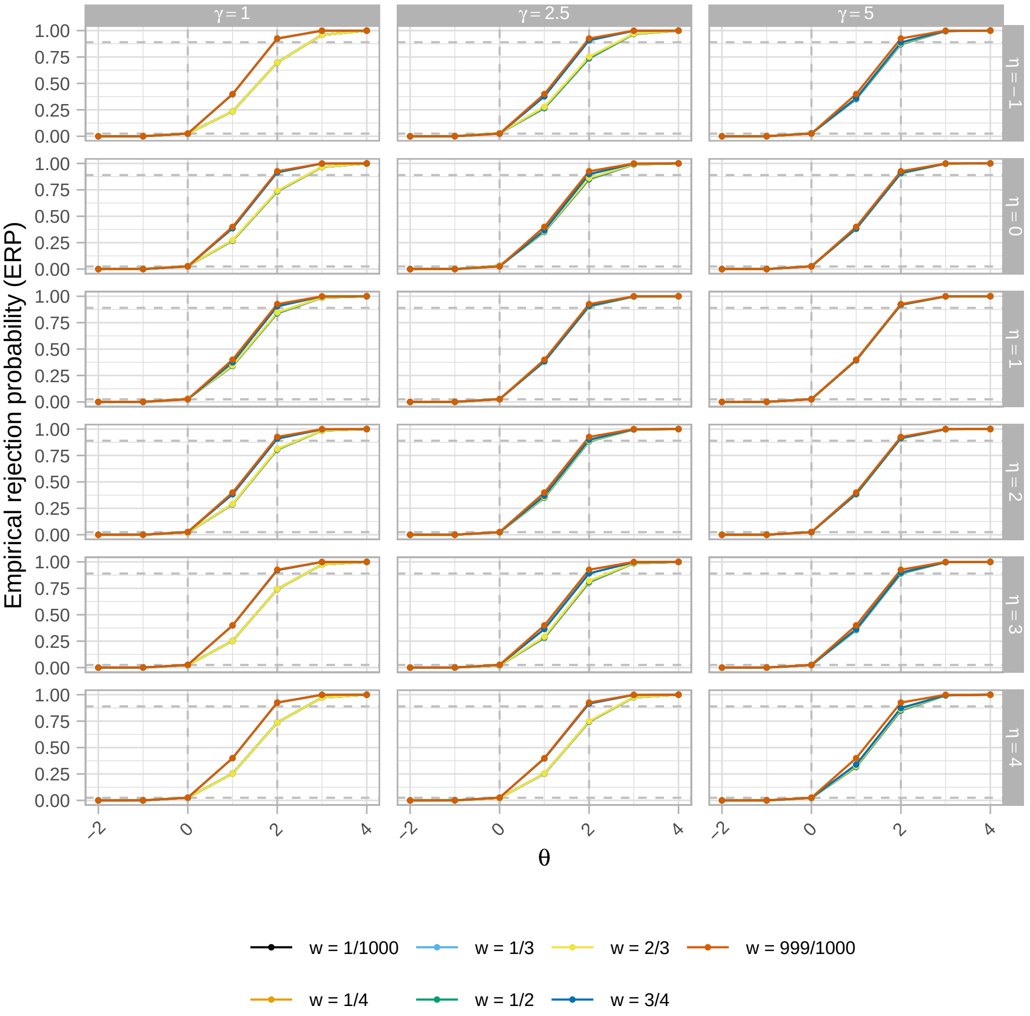

Figure S41: The empirical rejection probability (ERP), as a function of w and θ, of the response adaptive (RA) stepped-wedge cluster randomised trial (SW-CRT) designs with $\{p_1, \dots, p_L\} = \{2\}$ for different combinations of η and γ, in Trial Design Scenario 3 (TDS3). The dashed lines indicate the desired type-I and type-II error-rates.

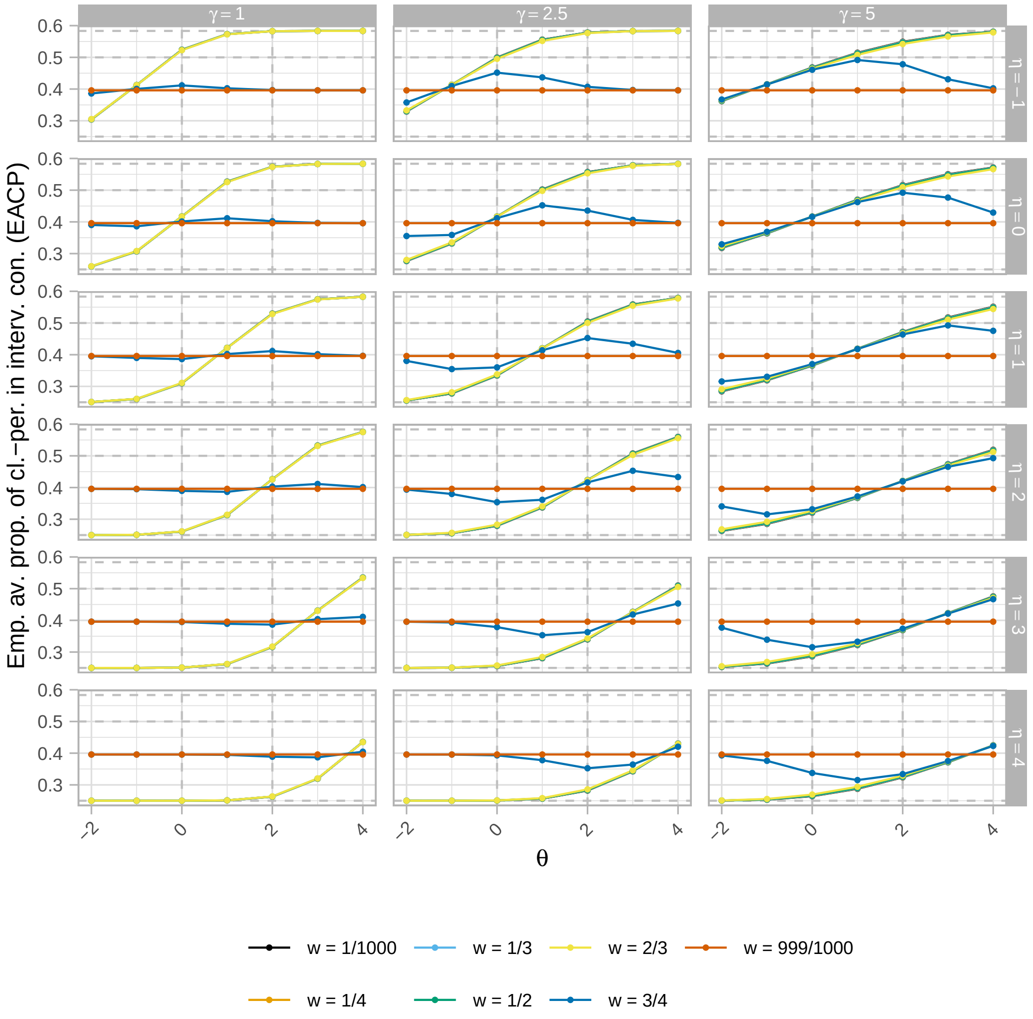

Figure S42: The empirical average proportion of cluster-periods spent in the intervention condition (EACP), as a function of w and θ, of the response adaptive (RA) stepped-wedge cluster randomised trial (SW-CRT) designs with $\{p_1, \ldots, p_L\} = \{2\}$ for different combinations of η and γ, in Trial Design Scenario 3 (TDS3). The dashed lines indicate the minimal, initially planned, and maximal values of the EACP based on $X = X_{p_1}$.

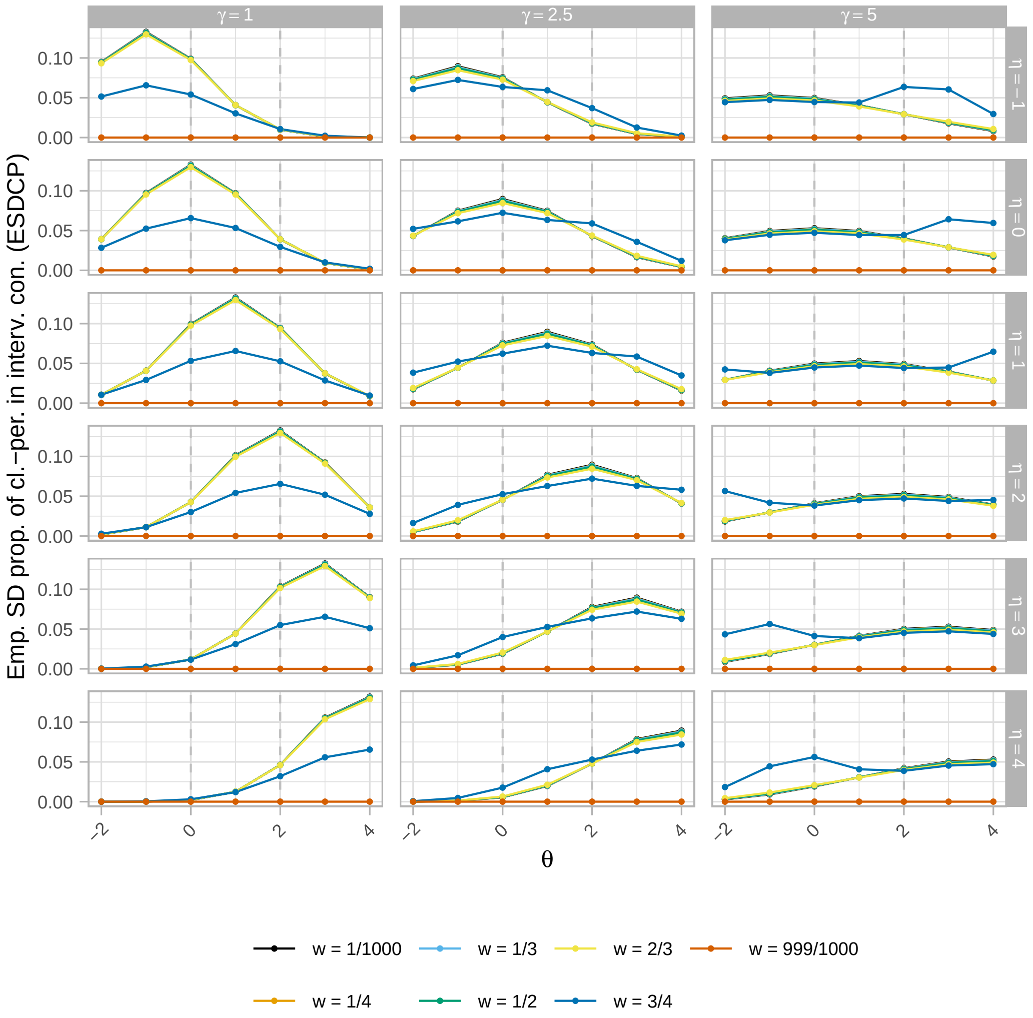

Figure S43: The empirical standard deviation of the proportion of cluster-periods spent in the intervention condition (ESDCP), as a function of w and θ, of the response adaptive (RA) stepped-wedge cluster randomised trial (SW-CRT) designs with $\{p_1, \ldots, p_L\} = \{2\}$ for different combinations of η and γ, in Trial Design Scenario 3 (TDS3).

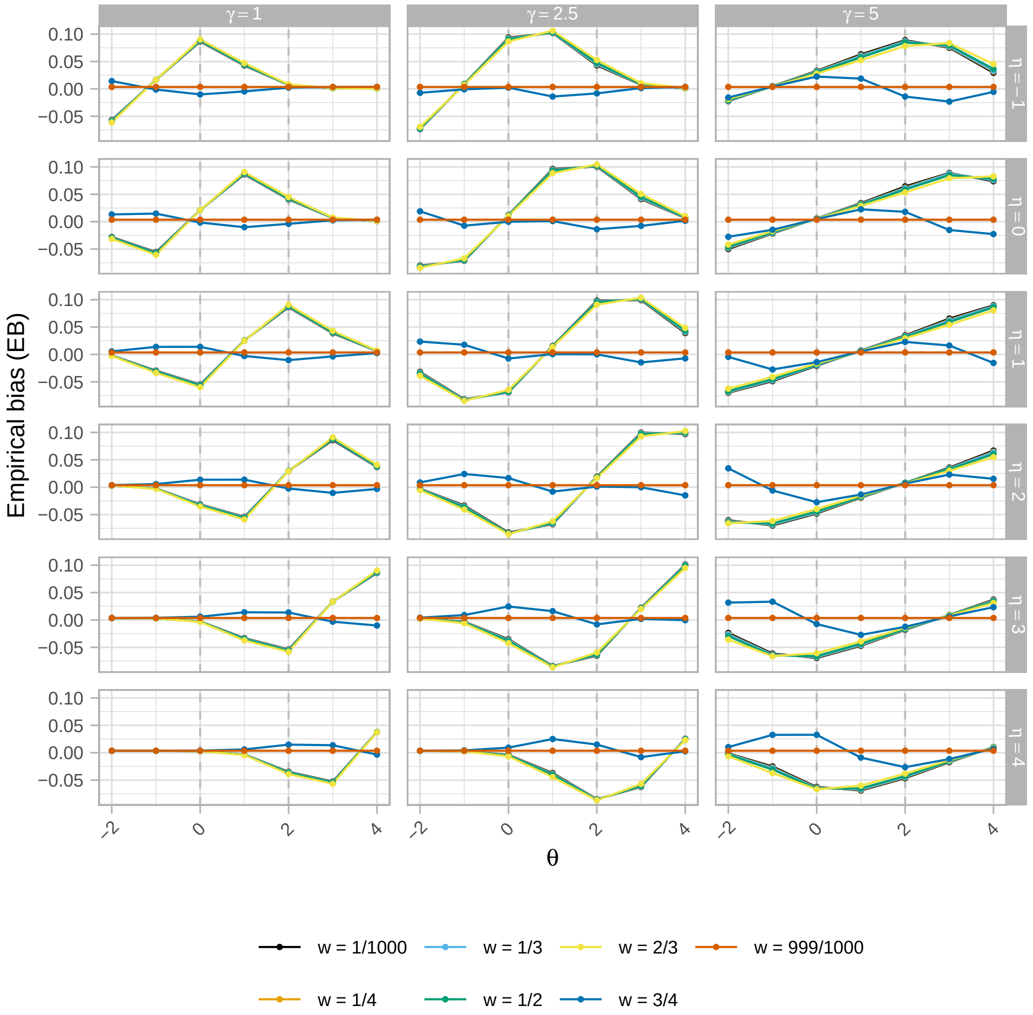

Figure S44: The empirical bias (EB), as a function of w and θ, of the response adaptive (RA) stepped-wedge cluster randomised trial (SW-CRT) designs with $\{p_1, \ldots, p_L\} = \{2\}$ for different combinations of η and γ, in Trial Design Scenario 3 (TDS3).

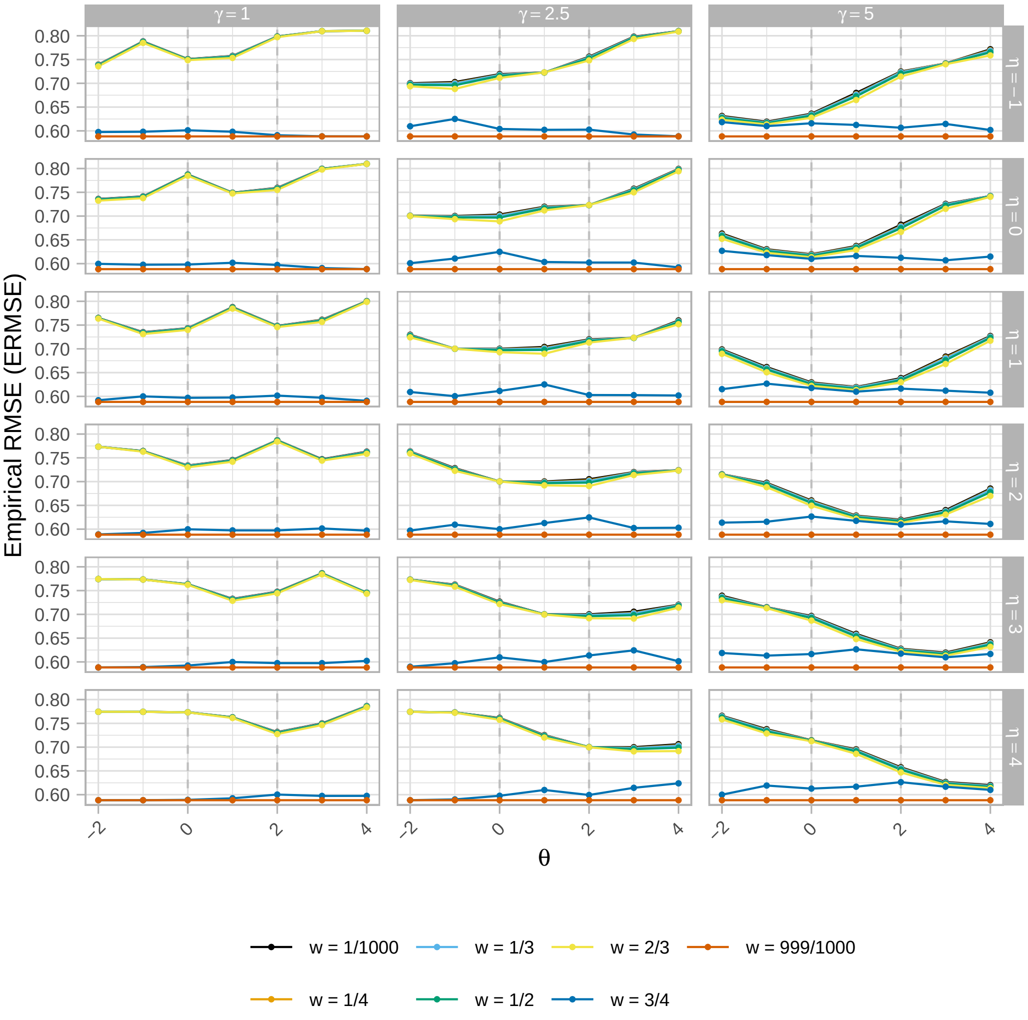

Figure S45: The empirical root-mean-square error (ERMSE), as a function of w and θ, of the response adaptive (RA) stepped-wedge cluster randomised trial (SW-CRT) designs with $\{p_1, \dots, p_L\} = \{2\}$ for different combinations of η and γ, in Trial Design Scenario 3 (TDS3).

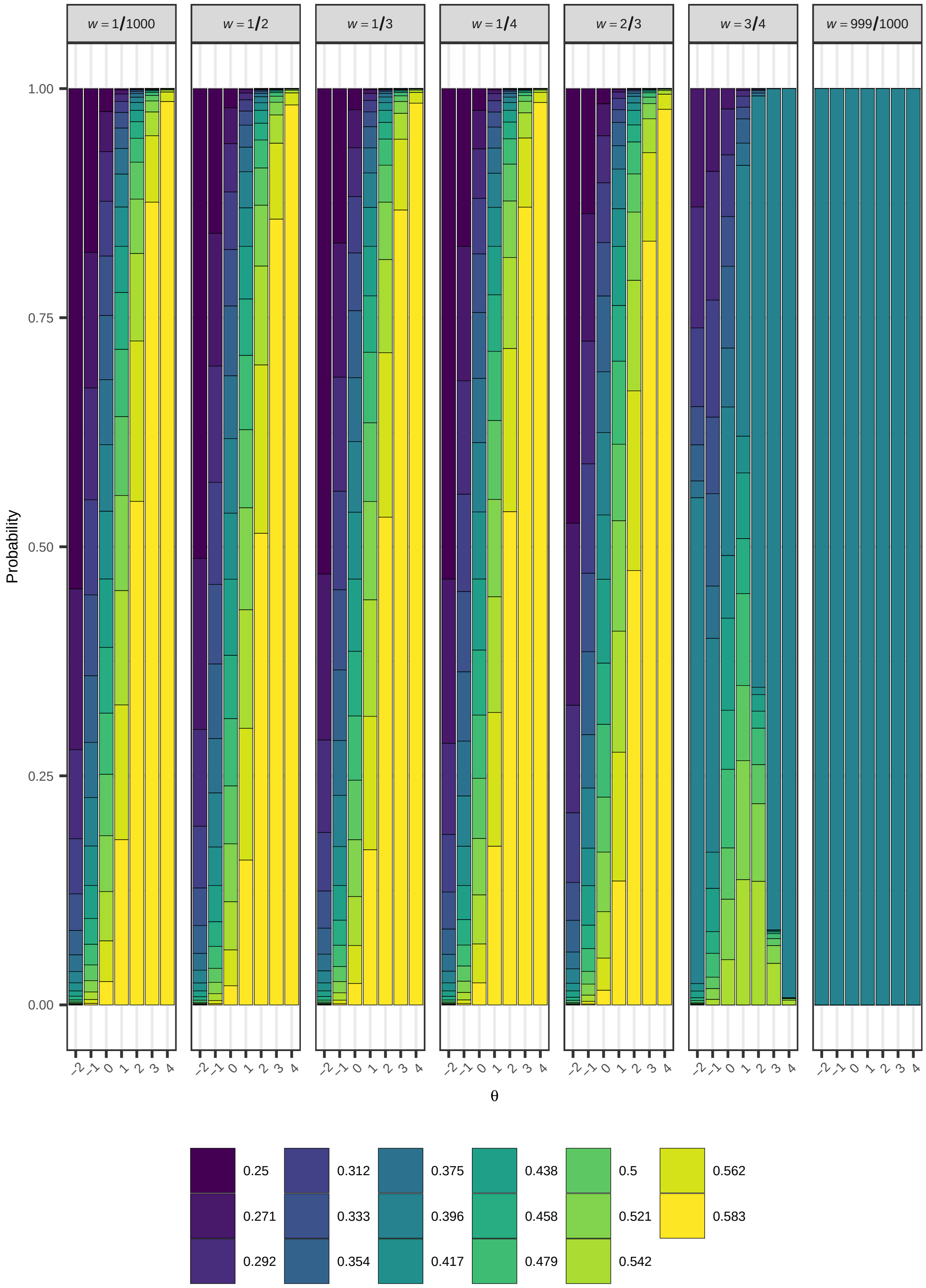

Figure S46: The empirical probability mass function of the proportion of cluster-periods spent in the intervention condition, as a function of θ and w, of the response adaptive (RA) stepped-wedge cluster randomised trial (SW-CRT) design with $\eta = 0$, $\gamma = 2.5$, and $\{p_1, \dots, p_L\} = \{2\}$, in Trial Design Scenario 3 (TDS3).

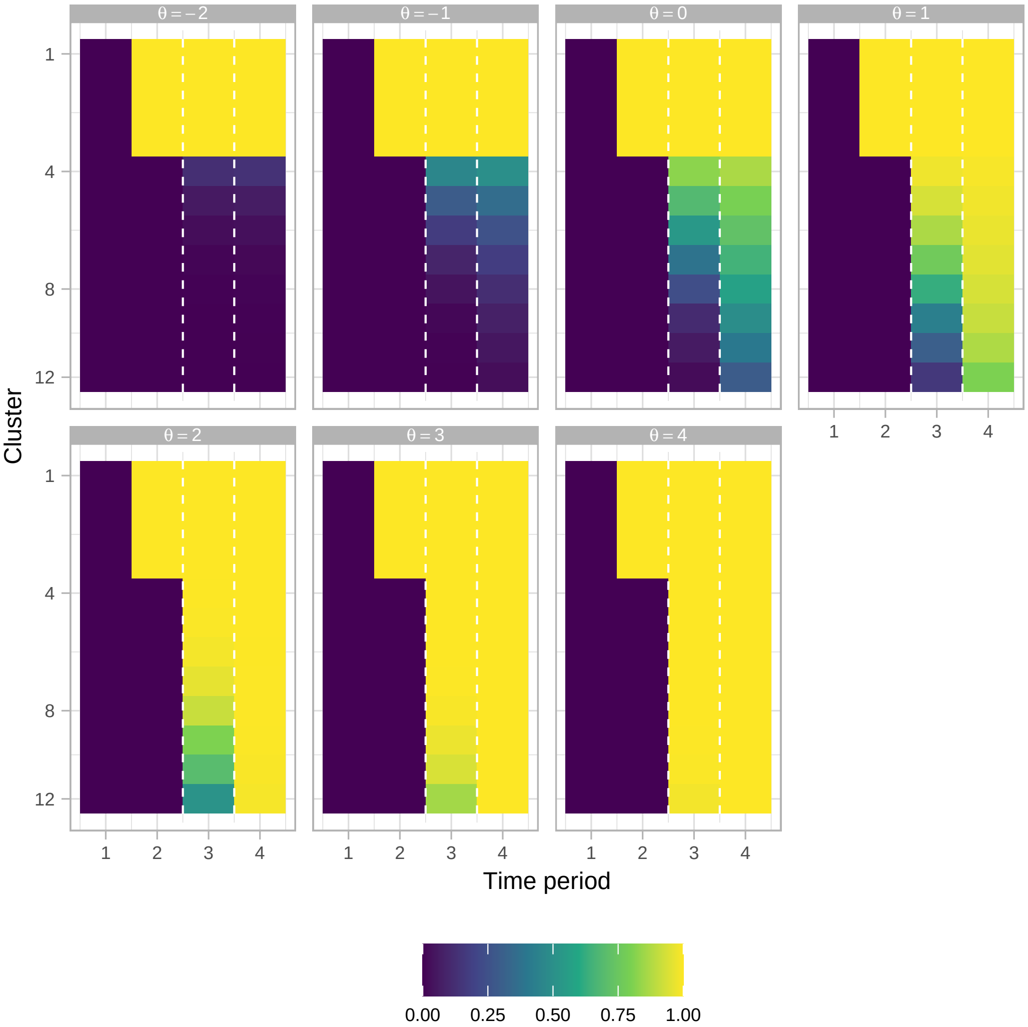

Figure S47: The empirical average final allocation matrix ($\bar{X}_P$), as a function of θ, of the response adaptive (RA) stepped-wedge cluster randomised trial (SW-CRT) design with $\eta = 0$, $\gamma = 2.5$, $w = 1/2$, and $\{p_1, \dots, p_L\} = \{2, 3\}$, in Trial Design Scenario 3 (TDS3). The dashed lines indicate the timing of the interim analyses.

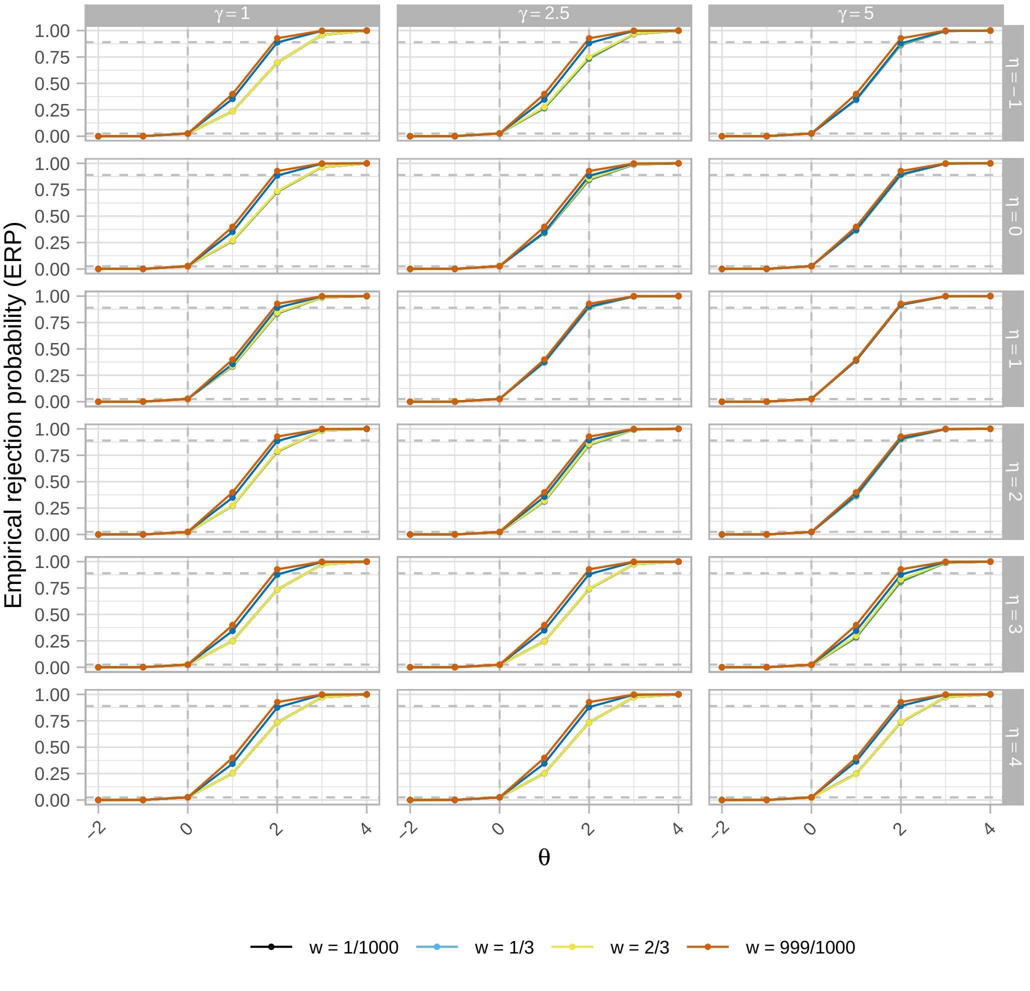

Figure S48: The empirical rejection probability (ERP), as a function of w and θ, of the response adaptive (RA) stepped-wedge cluster randomised trial (SW-CRT) designs with $\{p_1, \dots, p_L\} = \{2, 3\}$ for different combinations of η and γ, in Trial Design Scenario 3 (TDS3). The dashed lines indicate the desired type-I and type-II error-rates.

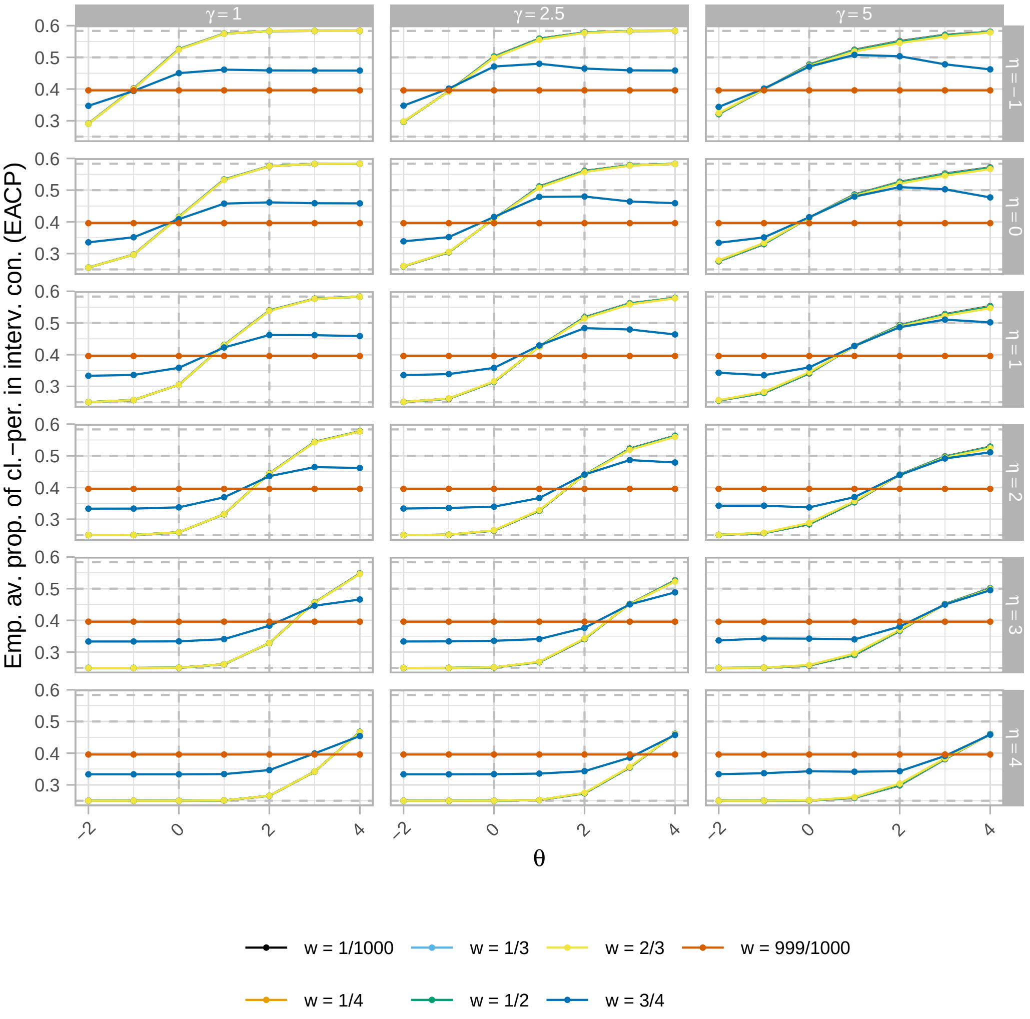

Figure S49: The empirical average proportion of cluster-periods spent in the intervention condition (EACP), as a function of w and θ, of the response adaptive (RA) stepped-wedge cluster randomised trial (SW-CRT) designs with $\{p_1, \dots, p_L\} = \{2, 3\}$ for different combinations of η and γ, in Trial Design Scenario 3 (TDS3). The dashed lines indicate the minimal, initially planned, and maximal values of the EACP based on $X = X_{p_1}$.

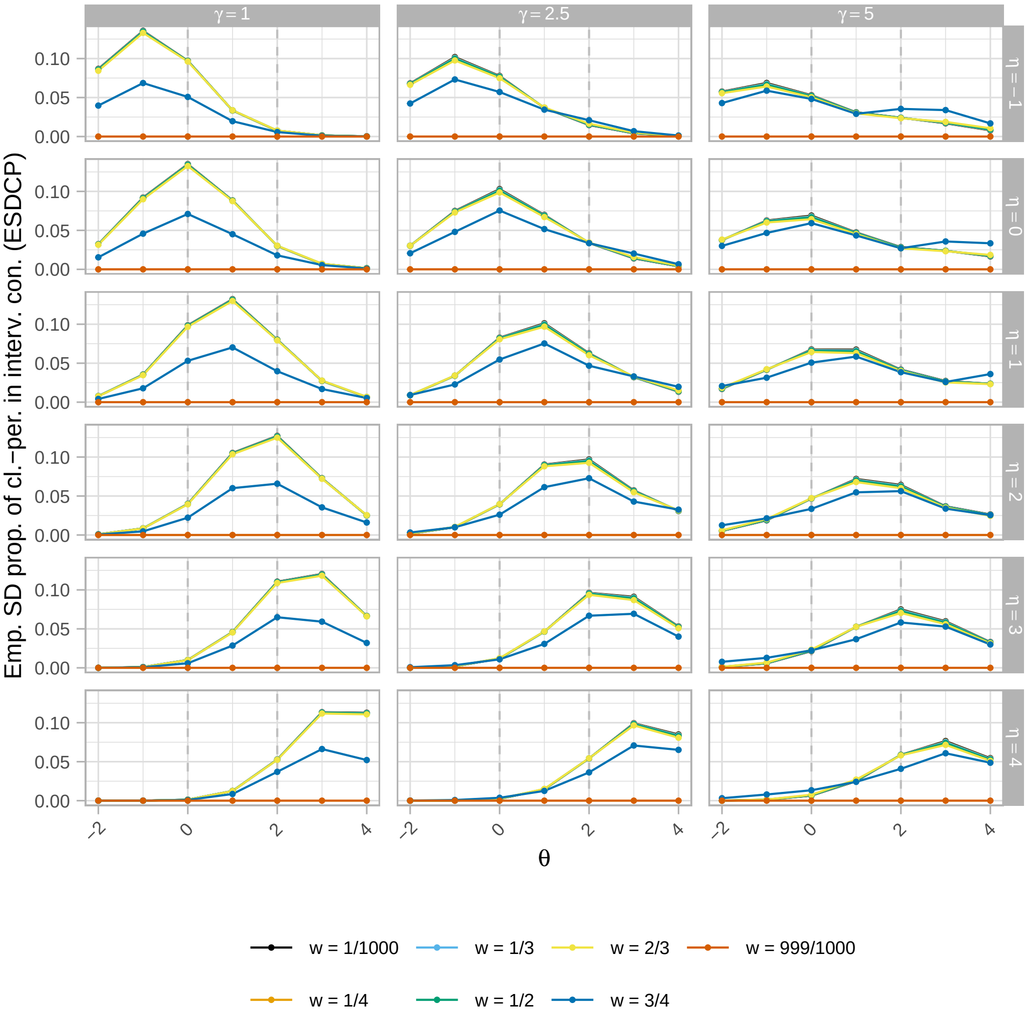

Figure S50: The empirical standard deviation of the proportion of cluster-periods spent in the intervention condition (ESDCP), as a function of w and θ, of the response adaptive (RA) stepped-wedge cluster randomised trial (SW-CRT) designs with $\{p_1, \ldots, p_L\} = \{2, 3\}$ for different combinations of η and γ, in Trial Design Scenario 3 (TDS3).

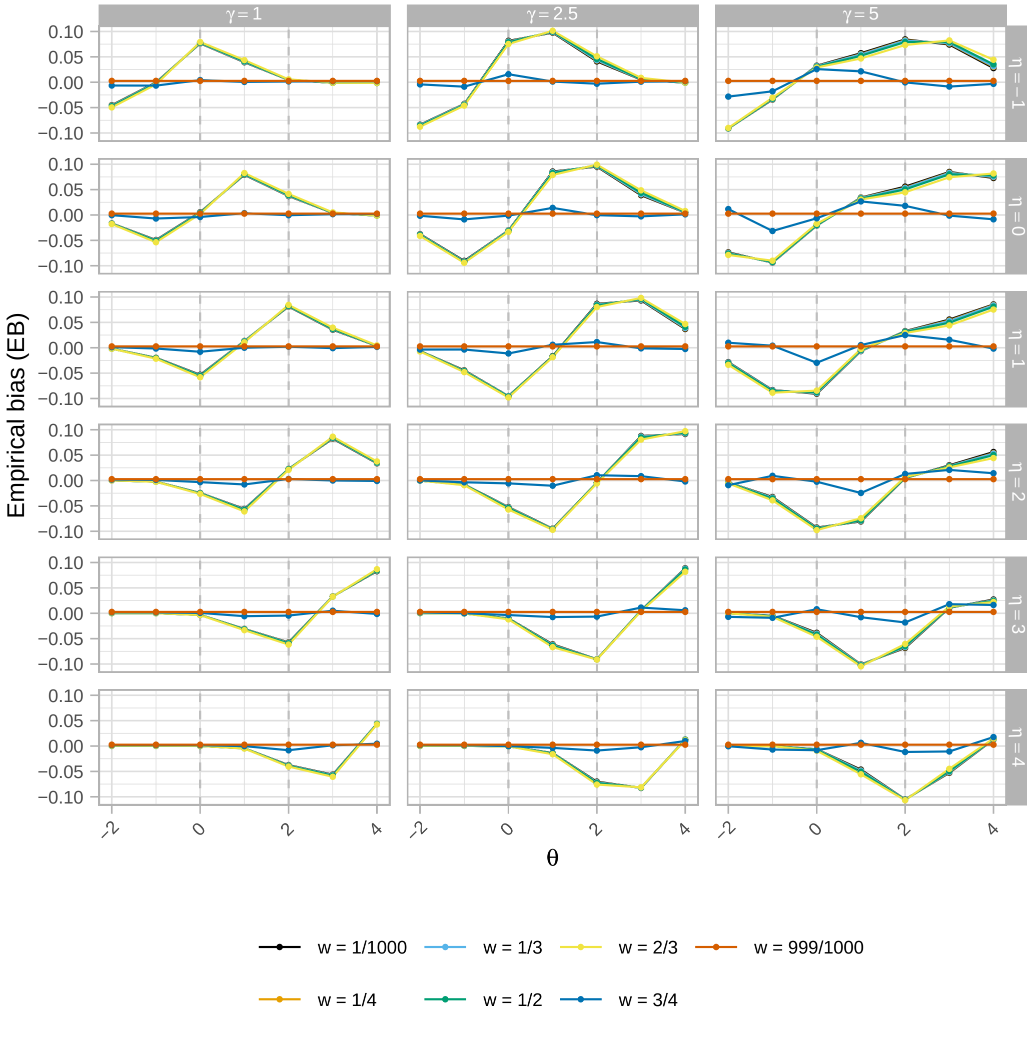

Figure S51: The empirical bias (EB), as a function of w and θ, of the response adaptive (RA) stepped-wedge cluster randomised trial (SW-CRT) designs with $\{p_1, \dots, p_L\} = \{2, 3\}$ for different combinations of η and γ, in Trial Design Scenario 3 (TDS3).

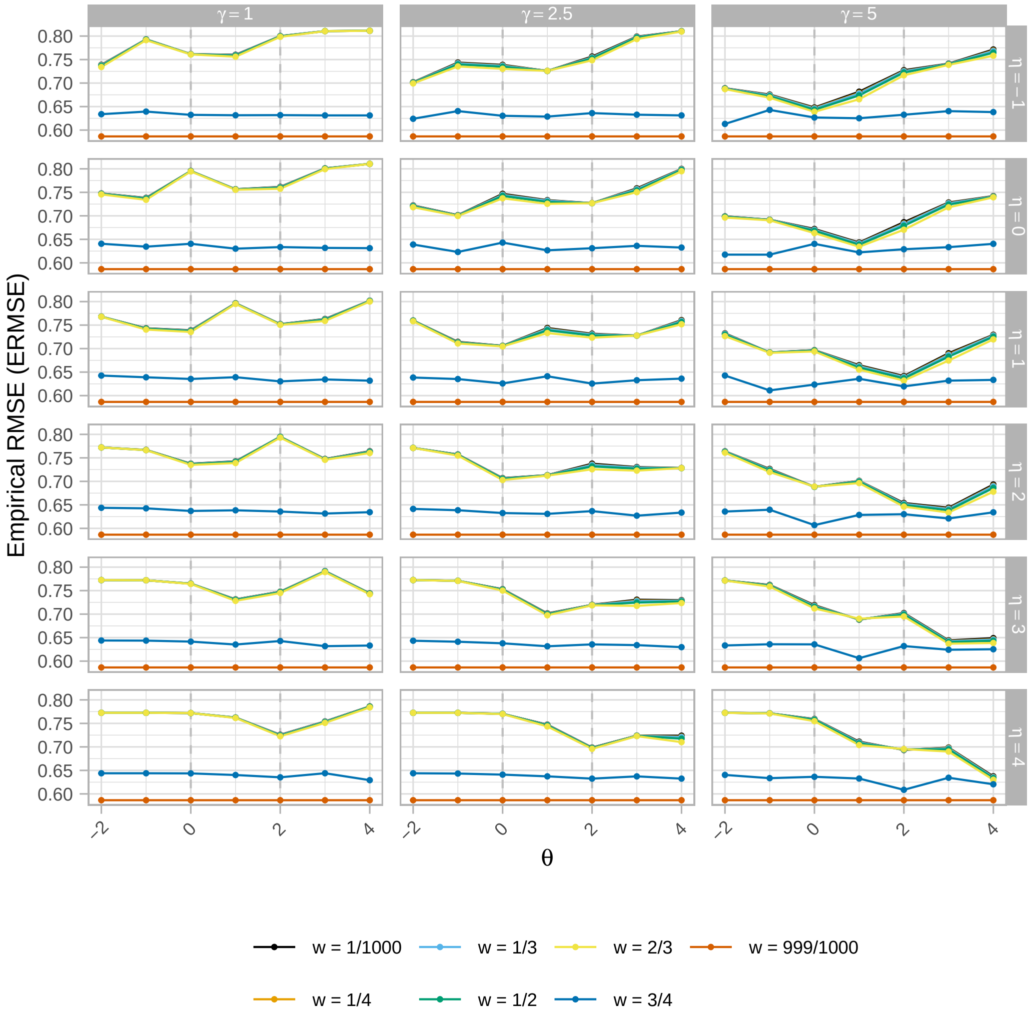

Figure S52: The empirical root-mean-square error (ERMSE), as a function of w and θ, of the response adaptive (RA) stepped-wedge cluster randomised trial (SW-CRT) designs with $\{p_1, \dots, p_L\} = \{2, 3\}$ for different combinations of η and γ, in Trial Design Scenario 3 (TDS3).

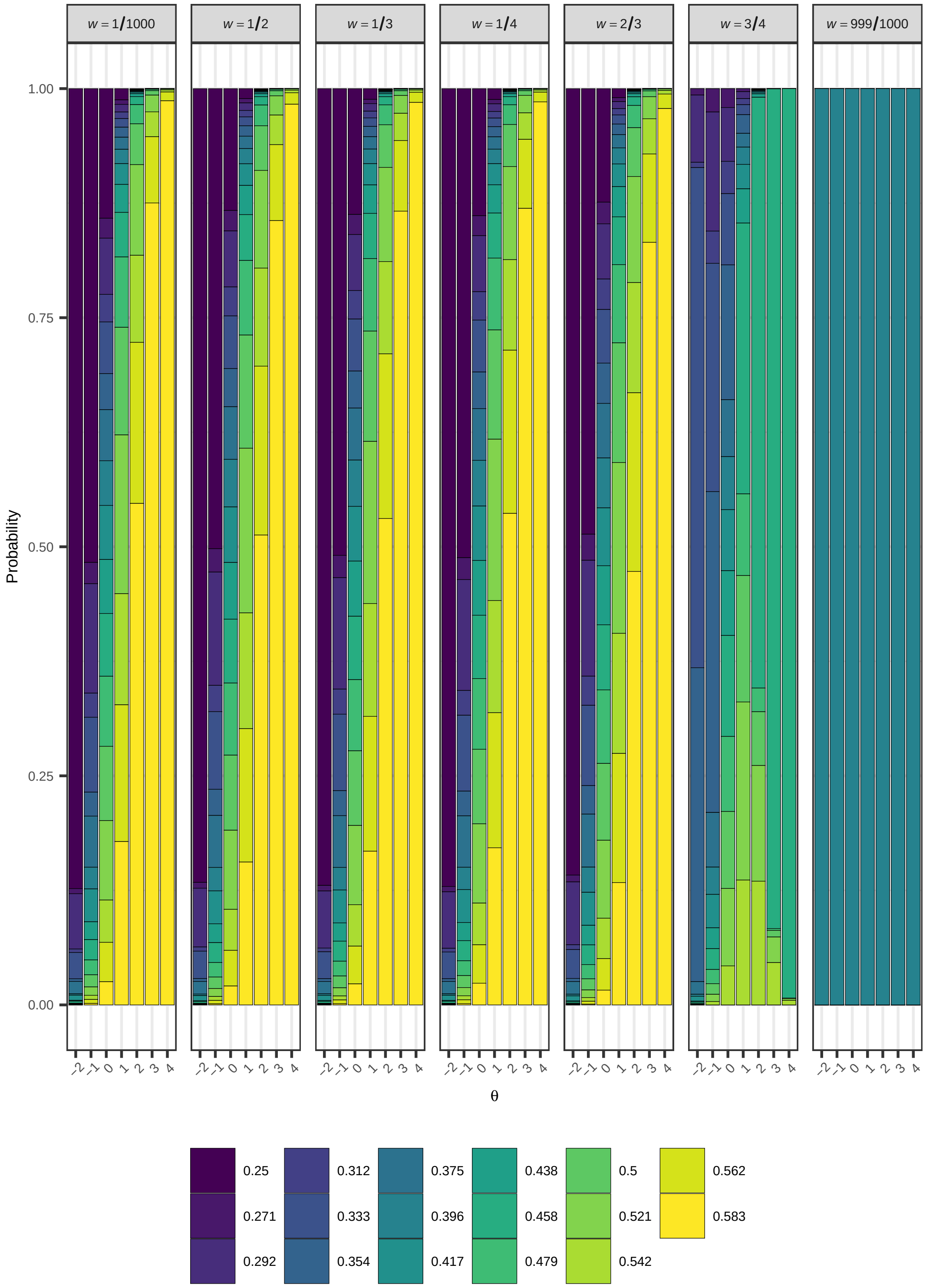

Figure S53: The empirical probability mass function of the proportion of cluster-periods spent in the intervention condition, as a function of θ and w, of the response adaptive (RA) stepped-wedge cluster randomised trial (SW-CRT) design with $\eta = 0$, $\gamma = 2.5$, and $\{p_1, \dots, p_L\} = \{2, 3\}$, in Trial Design Scenario 3 (TDS3).

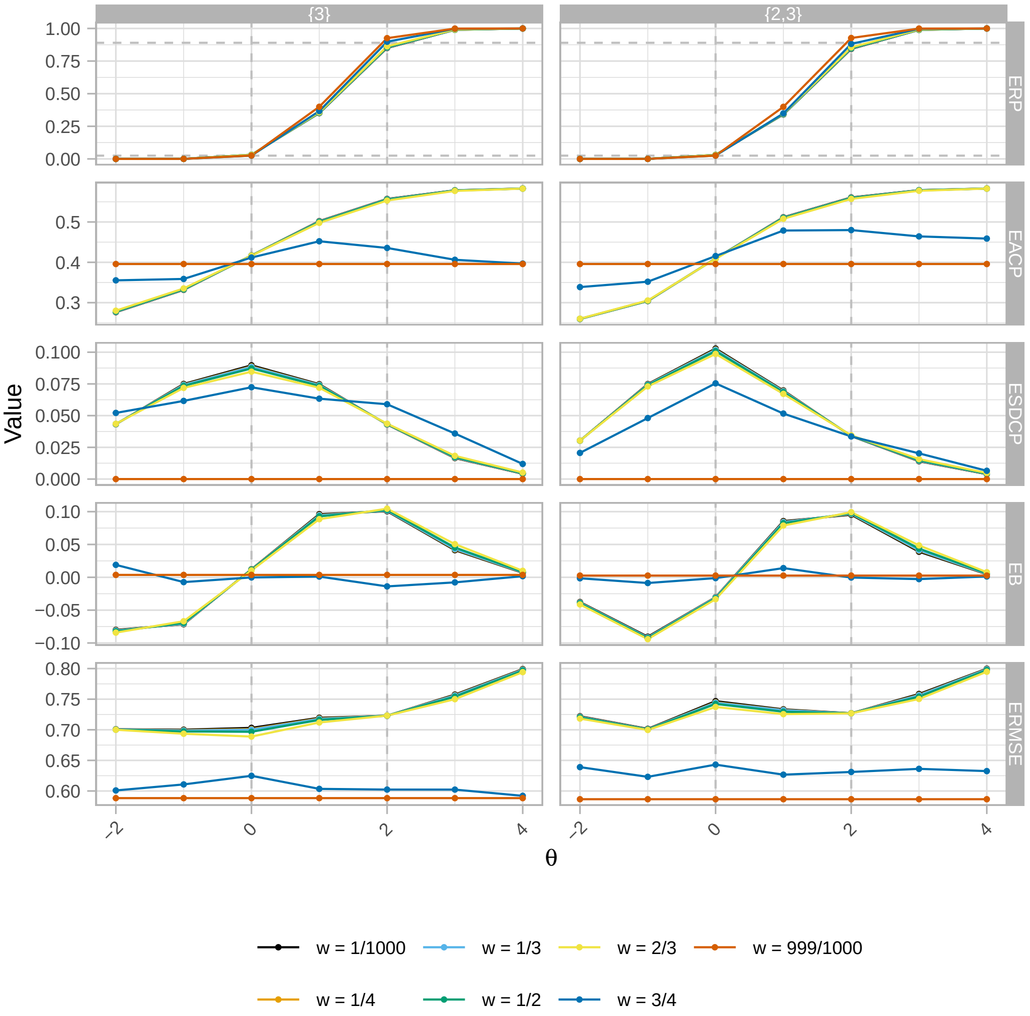

Figure S54: The empirical rejection probability (ERP), average proportion of cluster-periods spent in the intervention condition (EACP), standard deviation of cluster-periods spent in the intervention condition (EACP), bias (EB), and root-mean-square error (RMSE), as functions of w and θ, of the response adaptive (RA) stepped-wedge cluster randomised trial (SW-CRT) designs with $\eta = 0$ and $\gamma = 2.5$, for different values of $\{p_1, \dots, p_L\}$, in Trial Design Scenario 3 (TDS3). The dashed lines in the ERP plot indicate the desired type-I and type-II error-rates. In the EACP plot they indicate the minimal, initially planned, and maximal values of the EACP based on $X = X_{p_1}$.
